# Solid-State and Theoretical Investigations of Some Banister-Type Macrocycles with 2,2’-Aldoxime-1,1’-Biphenyl Units

**DOI:** 10.3389/fchem.2021.750418

**Published:** 2021-10-06

**Authors:** Ioan Stroia, Ionuţ -Tudor Moraru, Maria Miclăuş, Ion Grosu, Claudia Lar, Ioana Georgeta Grosu, Anamaria Terec

**Affiliations:** ^1^ Department of Chemistry and SOOMCC, Faculty of Chemistry and Chemical Engineering, Babes-Bolyai University, Cluj-Napoca, Romania; ^2^ Faculty of Chemistry and Chemical Engineering, Metalomica Research Centre, Babes-Bolyai University, Cluj-Napoca, Romania; ^3^ National Institute for Research and Development of Isotopic and Molecular Technologies, Cluj-Napoca, Romania

**Keywords:** chirality, atropisomers, geländer/banister molecules, molecular modeling, DFT mechanistic investigations, racemization barriers

## Abstract

In the context of helical chirality, bridging of biphenyl units leads to banister-type compounds and the stability of the resulted atropisomers may increase dramatically if suitable changes are performed in the linker unit that coils around the biphenyl moiety. A rigorous density functional theory (DFT) study was conducted for macrocycles containing rigid oxime ether segments connected to the biphenyl backbone in order to determine how the rotation barriers are influenced by the presence of either a flexible oligoethyleneoxide or a more rigid *m*–xylylene component in the macrocycle. The calculated values for the racemization barrier were in good agreement with those obtained experimentally and confirm the benefit of introducing a more rigid unit in the macrocycle on the stability of atropisomers. Solid-state data were obtained and computed data were used to assess the contribution brought by supramolecular associations observed in the lattice to the stabilization of the crystal structure. Beside introducing rigidity in the linker, complexation of flexible macrocycles with alkali metal ions is also contributing to the stability of atropisomers, leading to values for the racemization barrier matching that of the rigid macrocycle. Using diethylammonium cation as guest for the macrocycle, a spectacular increase in the barrier to rotation was observed for the resulted pseudo[2]rotaxane.

## Introduction

Chirality is one of the most studied properties of molecules, mostly due to its importance in biological systems. Though, historically, chirality was first connected to tetrahedral carbon carrying four different groups (Eliel et al., 1994), other chiral stereogenic units beside asymmetric centers were recognized soon and a keen interest emerged for valorization of molecules exhibiting axial (Alkorta et al., 2012; Glunz, 2018; Jamieson et al., 2018; Mancinelli et al., 2020; Cheng et al., 2021), planar (Grimme et al., 1998; Hassan et al., 2018; Namba et al., 2020) or helical chirality (Urbano, 2003; Gingras et al., 2013; Rickhaus et al., 2016).

**Chart 1:**
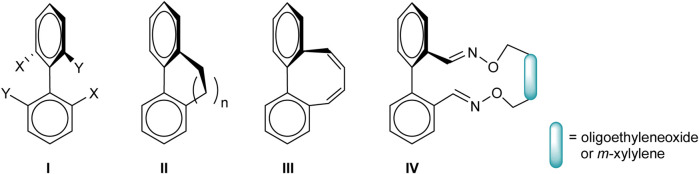


Classical examples of molecules with a chiral axis are biaryls (with biphenyls as particular case) and the first example of resolved enantiomers (*o,o’*-dinitro-*o,o’*-difenic acid) was described about a century ago (Christie and Kenner, 1922). The axial chirality of biphenyls appropriately substituted at positions 2, 2’, 6 and 6’ (I, Chart 1) along with other types of axially chiral molecules is well documented (Wencel-Delord et al., 2015; Renzi, 2017; Kanarasamy et al., 2015; Toşa et el., 2009; Piron et al., 2009; Terec et al., 2004; Grosu et al., 1998; Grosu et al., 1995). A key feature for the stereochemistry of biphenyls is the efficient hindrance of the rotation around the *sp* ^*2*^ *-sp* ^*2*^ bond and their optically active forms are named atropisomers. However, the racemization processes (due to the overcoming of the barrier for the rotation around the Ph-Ph bond) can occur in time and/or at high temperatures and Öki’s rather arbitrary definition of the condition of isomers to be labeled as atropisomers stipulates a half-life of at least 1000 s (around 22.3 kcal mol^−1^ at 300 K as free energy barrier) (Öki, 1983).

**Chart 2:**
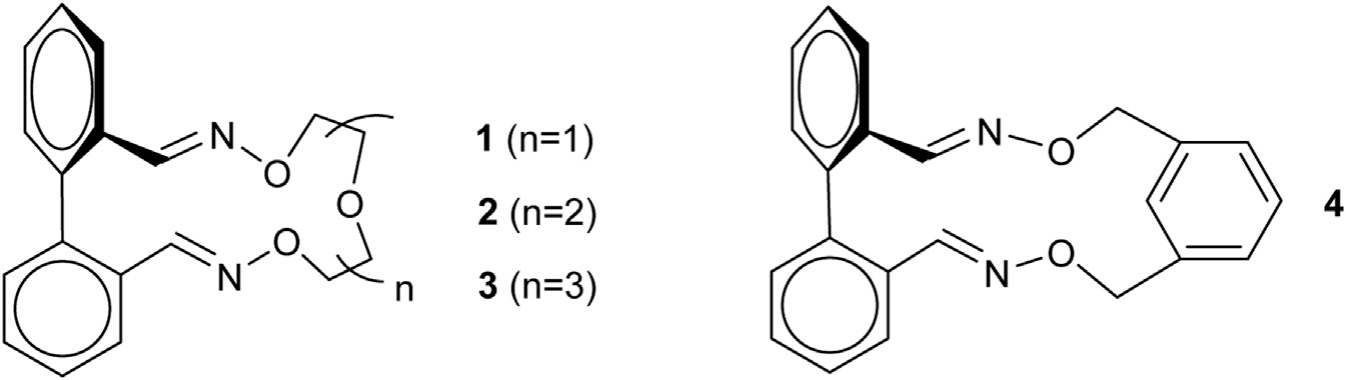


A step forward in the field was brought by the axial helical structures named *geländer* (the German word for *banister*) molecules (II-IV, Chart 1), a concept introduced by Vögtle referring to bridged 2,2’-biphenyls, leading to increase hindrance in the racemization process (Mislow et al., 1964; Kiupel et al., 1998; Eshdat et al., 1999; Wolf and Bentley, 2013; Zhang et al., 2016). These compounds reveal spectacular helical structures and high optical activity in a range close to that found for [n]helicenes (Modjewski et al., 2009; Takaishi et al., 2010; Rotzler et al., 2013; Rickhaus et al., 2014; Bannwart et al., 2017; Mannancherry et al., 2018; Bannwart et al., 2021).

The helical conformation appears by balancing the minimisation of the steric interactions that arise in the planar conformation of the biphenyl unit against the strain induced by the twisting of the molecule. So, the molecule has to be simultaneously rigid and flexible enough. Despite expectations, the racemisation process in the case of simple banister compounds (II, Chart 1) showed quite low barriers (12.5 kcal mol^−1^ at 262 K for *n* = 2 and 23.0 kcal mol^−1^ at 293 K for *n* = 3), while the hindrance of the rotation around the Ph-Ph bond increased in the derivative exhibiting two double bonds in the bridges (III, 1,3-butadien-1,4-diyl bridge; 29.6 kcal mol^−1^ at 350 K) (Müllen et al., 1990). In our previous work, we were able to separate the enantiomers of some bridged 2,2’-dioxime-1,1’-biphenyl of type IV with various bridges (Chart 2) and to measure their racemisation barriers (24–25 kcal mol^−1^ at 323 K for 1–3 with oligoethyleneoxide units and 31 kcal mol^−1^ at 393 K for 4 containing a *meta*-xylylene group) (Lar et al., 2019). A relation between these barriers to rotation and the structural features of the macrocycles was observed: the larger the size of the macrocycle, the more stable the banister and a higher barrier when the more rigid *m*-xylylene unit was employed.

In the context of our previous experience with correlating experimental solution and solid-state structural data with computational studies (Grosu et al., 2017; Grosu et al., 2020), we were interested in elaborating a computational work to access information about the behavior of both the ether oxime units and the bridge in the racemization process and how complexation of our ethyleneoxide bridged banisters (1–3) with different alkali metal ions (Li^+^, Na^+^, K^+^) and diethylammonium ion as organic cation would influence the stability of the enantiomers, aiming to increase the barrier to rotation of these atropisomers. Moreover, as we managed to grow single crystals of 4, a thorough investigation of the crystal molecular structure and corroboration of calculations based on the solid-state data are expected to offer insight into the role of various contacts to the stabilization of the supramolecular architecture.

## Materials and Methods

Density functional theory (DFT) studies were performed using the *Gaussian 09* package (Frisch et al., 2009). The PBE0 hybrid functional was chosen as a DFT method due to its widely spread applicability on a broad range of compounds, while the D3 Grimme’s dispersion corrections (Grimme et al., 2010) were also employed in order to estimate long range dispersion effects for the investigated species. Thus, employing PBE0-D3 along with the valence triple-zeta Def2-TZVP (Schäfer et al., 1994; Rappoport and Furche, 2010) basis set, all the structures were optimized in the gas phase, without any symmetry constraints. Optimization criteria were set to tight. In order to characterize the nature of the stationary points and to determine the zero-point energy and the thermal corrections, analytic second derivative calculations were performed on the optimized structures. Within all calculations, the integration grid used was of 99 radial shells and 950 points for each shell (99,950), representing the *ultrafine* grid in *Gaussian 09*. In order to reproduce as close as possible the experimental conditions, for the DFT calculations carried out on compounds 1, 2 and 3 the temperature was set to 323 K, whilst for macrocycle 4–393 K. In this way, computed racemization barriers were obtained at the same temperatures as those determined experimentally. In addition, intrinsic reaction coordinate (IRC) analyses were performed at the PBE0-D3/Def2-SVP (Grimme et al., 2010) level of theory on each transition state (TS), in order to confirm that suggested TSs connect the considered minima.

X-Ray crystallography data for compound 4 are presented in the Supplementary Material. The ring centroids and intermolecular interactions were calculated using PLATON (Spek, 2009). The representation of the crystallographic data and calculations of the angles between the planes were executed using Diamond software (Diamond software, 2021).

## Results and Discussions

### Structural Features and Strain Energies

**FIGURE 1:**
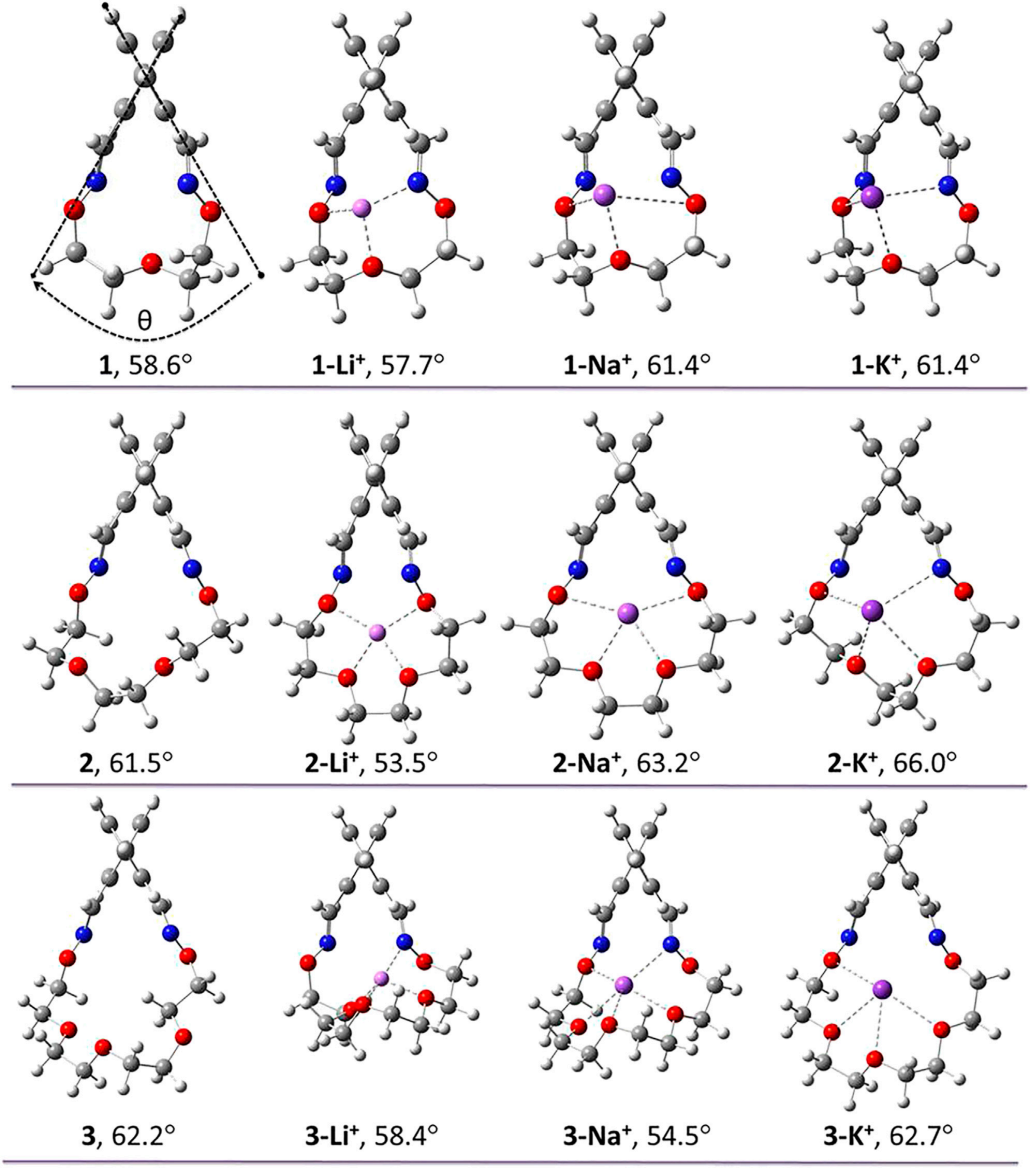
Side view and torsion angle for the computed molecular structures of macrocycles 1–3 and their alkali metal ion (Li^+^, Na^+^, K^+^) complexes. The arrow indicates the sense of the helix.

While the biphenyl backbone is a self-contained moiety of biphenyl-based banisters, the crucial part in geometry modulation is played by the linker. From a structural viewpoint, the torsion angle of the biphenyl unit is adjusted by the bridge in order to minimize all the strain energies of macrocycles. The optimized ground state (GS) geometries of our series of banisters indicate an increase in the dihedral angle (θ) from 58.6° for 1 to 62.2° for **3**, while an intermediate value of *ca*. 61.5° was computed in the case of derivative 2. This behavior is however expected, due to an increasing number of atoms in the linker from 1 to 3. The oligoethyleneoxide bridge is responsible for the chelatization of each cation (Li^+^, Na^+^, K^+^) and it adopts a proper conformation in which the macrocycle–cation interactions are maximized (Figure 1). Hence, the Li^+^ cation constricts all the dihedral angles, compared to the uncomplexed macrocycles, while the K^+^ one, with a higher ionic radius, expands the angles. Featuring the smallest bridge, macrocycle **1** is not able to increase the torsion angle to a value suitable for hosting the Na^+^ and K^+^ ions in the center of the cavity, resulting thus into 1-Na^+^ and 1-K^+^ species displaying the same torsion angle, with the complexed metal cation positioned outside the cavity. The *a priori* trend of torsion angles is broken down by the complexed macrocycle 3-Na^+^, in which the sodium cation interacts with the oxygen of one of the oxime groups (2.6 Å) and with the nitrogen of the other (2.5 Å) and, as a consequence, the torsion angle is constricted. In the case of 3-Li^+^, the cation does not interact with both ether oxime groups, namely the nitrogen atom of one ether oxime group coordinates to Li^+^ (2.0 Å), while the distance between the oxygen of the second ether oxime unit and Li^+^ is too large (3.8 Å) for an interaction. In other words, the Li^+^ is secluded in one part of the cavity and the relaxation of dihedral angle, relative to 3-Na^+^, is allowed.

**FIGURE 2:**
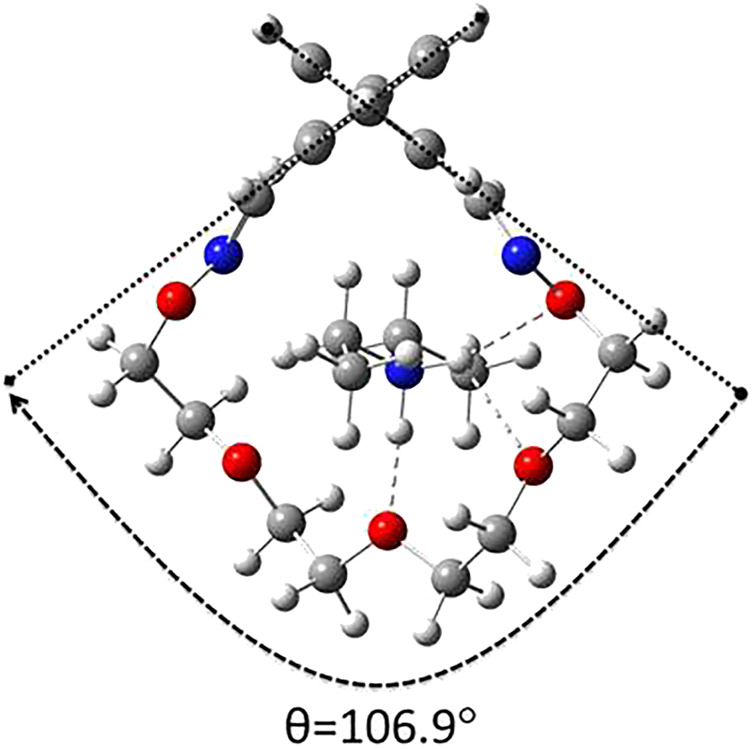
Side view and torsion angle for the DFT optimized molecular geometry of the pseudo[2]rotaxane 3-Et_2_NH_2_^+^.

We have also investigated the impact of an organic cation, namely diethylammonium ion (Et_2_NH_2_^+^), on the structural features of the investigated macrocycles. Among the ether oxime macrocycles 1–4, only macrocycle 3 is suitable in size for the complexation of Et_2_NH_2_^+^. In the pseudo[2]rotaxane 3-Et_2_NH_2_^+^ model the cavity opening is maximized in order to accommodate the diethyl ammonium axle (Figure 2), whereas the torsion angle is comparable to those found in the cycloparaphenylene-ethynylene bridged biphenyls (Wang et al., 2020).

**FIGURE 3:**
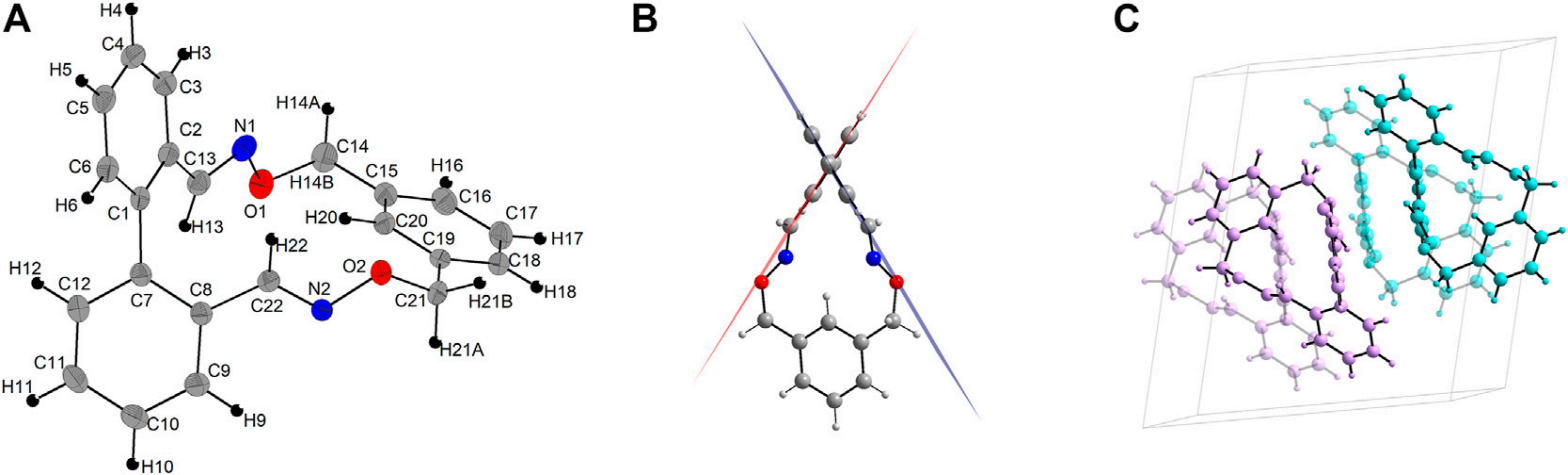
**(A)** Thermal ellipsoid representation (25% probability) of the molecular structure of 4. **(B)** Top view showing the torsion angle between the planes of phenyl groups in biphenyl backbone. **(C)** Packing view showing the four molecules present in the unit cell. The pair of enantiomers are depicted in two different colors for clarity (aqua for the *P* enantiomer and pink for the *M* enantiomer).

The-* single crystal molecular structure for 4 was obtained by X-Ray diffractometry of suitable crystals obtained by room temperature slow evaporation of a toluene solution (Figure 3A). Inspection of the molecular solid-state structure revealed a torsion angle of 62.7° in the biphenyl backbone as determined by measuring the angle between the planes determined by each benzene ring in this unit (Figure 3B). This value is in good agreement with that determined by means of DFT investigations, for which a computed value of *ca.* 65.5° was obtained.

**FIGURE 4:**
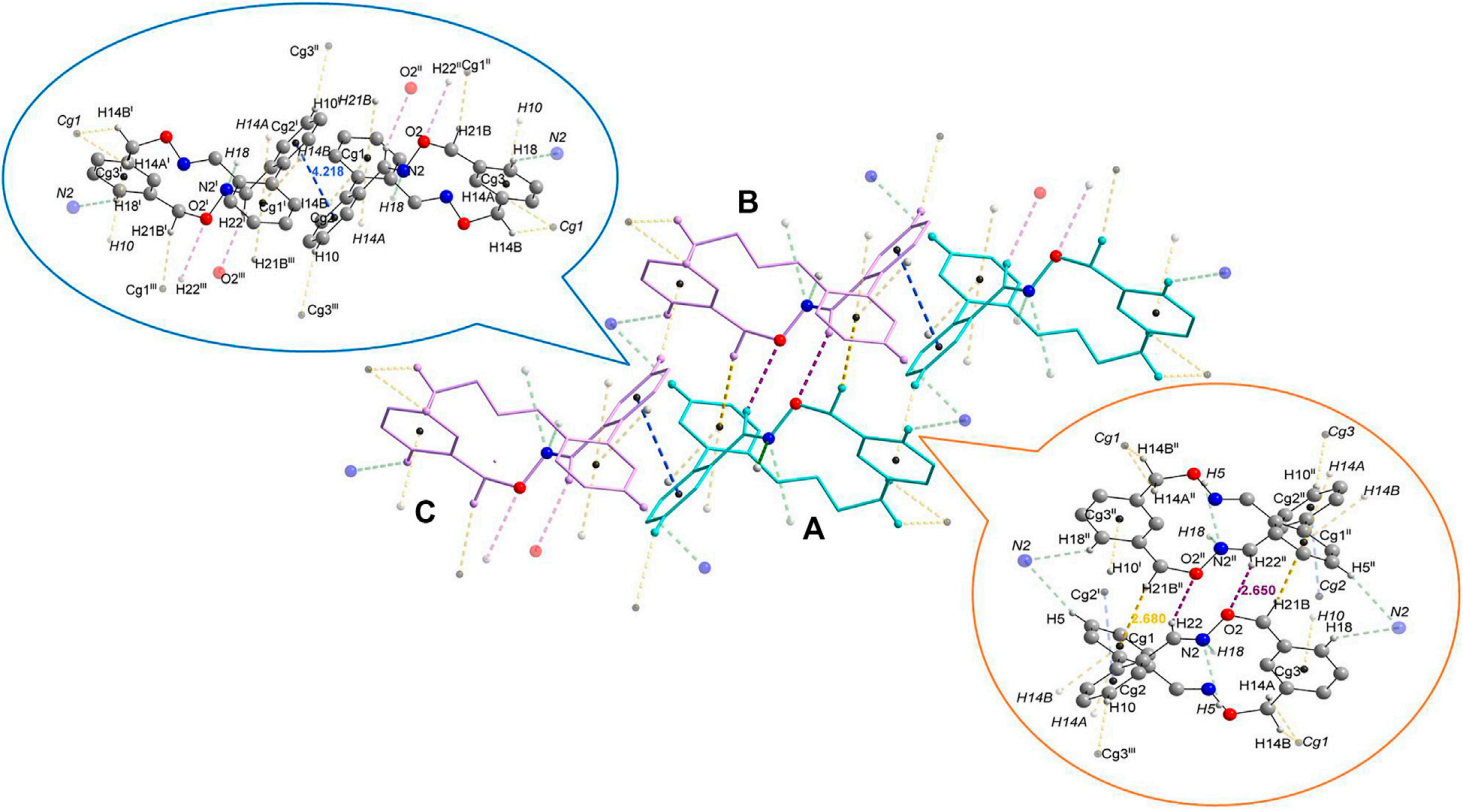
**Middle:** Capped stick representation of a fragment of the lattice presenting the identified intermolecular contacts. The two enantiomers were represented in different colors for clarity (aqua for the *P* enantiomer and pink for the *M* enantiomer). **Left inset:** Ball and stick representation of a fragment of the lattice displaying the **A-B** dimer formed by C–H···centroid and C–H···O contacts. **Right inset:** Ball and stick representation of a fragment of the lattice displaying the** A-C** dimer formed by π···π interactions. For clarity, in all representations the main contacts are drawn in full colors while the rest of the interactions are shown in more transparent colors (violet for C–H···O, blue for centroid···centroid, green for C–H···N, yellow for C–H···centroid contacts), the atoms involved in intermolecular interactions were highlighted/labelled and H atoms not involved in contacts were omitted.

Compound 4 crystalized in a monoclinic crystal system with the space group *P*21/n. The crystals contain both *M* and *P* enantiomers and there are four molecules in the unit cell (Figure 3C). There are two main association patterns in the lattice connecting molecules of opposite configuration (Figure 4, middle). A first dimer is formed by double contacts between the H in one oxime unit of one enantiomer (molecule **A**, *P* configuration) and the oxygen atom of the corresponding oxime moiety in the other enantiomer (molecule B, *M* configuration) (d_C–H22”···O2_ = 2.650 Å) and is reinforced by supplemental C–H···π interactions, again doubled, connecting one of the hydrogen atoms in the methylene of the *m*-xylylene group to the nearby centroid of one phenyl ring of the biphenyl moiety in the enantiomer (d_C–H21B”···Cg1_ = 2.680 Å) (Figure 4, right inset). The second, also heteromeric, association rely on *π···π* interactions between the centroid of the phenyl ring in the biphenyl scaffold (molecule **A**, *P* configuration) not involved in the previously described interactions and its counterpart in a third molecule (molecule **C**, *M* configuration) (d_Cg2–Cg2’_ = 4.218 Å) (Figure 4, left inset). The two phenyl rings involved in this *π···π* stacking are parallel to each other (a dihedral angle of 0.02°) and the perpendicular distance of centroid Cg2 (of the ring C1–…C6) on the ring C7’–···C12’ is 3.620 Å, with a 2.164 Å slippage. Beside these main interactions, there is a plethora of various intermolecular interactions to other adjacent molecules, most of them heteromeric (d_C–H18’···N2_ = 2.719 Å, d_C–H5’···N2_ = 2.759 Å, d_C–H10···Cg3_ = 2.752 Å), but few also homomeric contacts (d_C–H14A···Cg1_ = 2.988 Å and d_C–H14A···Cg1_ = 2.986 Å) which lead to supramolecular assemblies.

In order to gain further insight into the main heterodimer supramolecular associations of species 4, DFT analyses were additionally performed in this respect (for further details concerning the computational methods employed in estimating the interaction energies *see* Supplementary Figure S1 and the related discussion herein). Thus, computed data indicate that in the case of the A–B dimer, a stabilization energy of *ca.* 10 kcal mol^−1^ is achieved with respect to the separated species (Δ*E*°/dioxime molecule = 5 kcal mol^−1^). This energy gap is mainly related to reciprocal C–H···O and C–H···*π* contacts. By contrast, the energy gain resulting from the association of A and C in the A–C dimer by *π···π* interactions is lower, with a calculated valued of only 6.4 kcal mol^−1^ (Δ*E*°/dioxime molecule = 3.2 kcal mol^−1^).

Strain energies were also computed for macrocycles 1–4 by performing homodesmotic reactions (Supplementary Scheme S1). Somehow expected, increasing the number of atoms in the oligoethyleneoxide moiety does not enhance the steric repulsion in the equilibrium geometries of investigated macrocycles 1–3 due to greater flexibility. Even introducing a more rigid bridging group of similar size like 1,3-bis(methylene)benzene in 4 does not increase the steric repulsion above 1 kcal mol^−1^ (Supplementary Table S2).

### Racemization Processes

**FIGURE 5:**
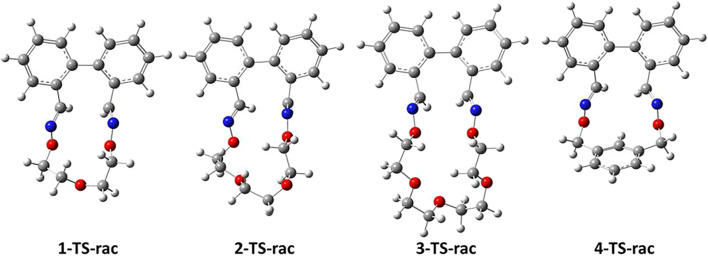
TS geometries corresponding to the racemization processes of macrocycles 1–4. Calculations were carried out optimized at the PBE0-D3/Def2-TZVP level of theory.

In order to understand whether the complexation of Li^+^, Na^+^ and K^+^ cations impact the racemization barriers of macrocycles 1–3 (banister 4 has no affinity for alkali ions because the bridge is lacking electronegative atoms able to sustain complexation), it is firstly vital to validate the computational methodology. In this regard, we compared the DFT racemization barriers for the uncomplexed banisters with the ones determined experimentally in solution using chiral HPLC or chiral Dynamic HPLC techniques as described earlier (Lar et al., 2019). The highest-energy TSs geometries corresponding to the racemization mechanism of macrocycles 1–4 are illustrated in Figure 5. In addition, IRC investigations further confirm that the proposed TSs connect the desired minima.

Comparisons between the computed and the experimentally determined barriers are illustrated in Table 1. Overall, the agreement between theoretical and experimental data is satisfactory. Yet, slight differences between theory and experiment may arise from *1*) the fact that DFT calculations were performed in gaseous phase and *2*) also because throughout the DFT study, racemization barriers were determined using the computed enthalpies (*i.e.* activation enthalpies of racemization - $\Delta H_{\mathrm{rac}}^{\neq }$ ) instead of the Gibbs-free energies of racemization ( $\Delta G_{\mathrm{rac}}^{\neq }$ ) employed in the experimental investigations. However, the gaps between theory and experiment are small enough (lower than 2.8 kcal mol^−1^ in all cases, Table 1) to ensure a good correlation between the DFT and the experimental studies. In order to confirm these theoretical findings, we have performed some extra DFT calculations employing the B3LYP hybrid functional (Supplementary Table S3). Thus, theoretical data obtained at the B3LYP-D3/Def2-TZVP level of theory reveal for macrocycle 1 a barrier of racemization of 26.4 kcal mol^−1^, for macrocycle 2 an activation energy of 26.8 kcal mol^−1^, while for derivative 3 a computed value of 27.5 kcal mol^−1^ is achieved. Thus, calculated energy gaps (PBE0-D3 *vs.* B3LYP-D3 functionals) for the racemization barrier of macrocycles 1–3 are low in all cases (the maximum gap is of *ca.* 0.4 kcal mol^−1^). As for banister 4, the racemization barrier obtained at the B3LYP-D3/Def2-TZVP level of theory is *ca.* 1.0 kcal mol^−1^ higher than the one determined using PBE0 hybrid functional.

**TABLE 1:** DFT (PBE0-D3/Def2-TZVP) and experimentally determined barrier for the racemization of banisters 1–4.

Compound	Racemization barrier (kcal mol^−1^)	
	Theoretical	Experimental
1	26.4	25.0
2	26.8	24.8
3	27.5	24.7
4	33.0	31.7

**FIGURE 6:**
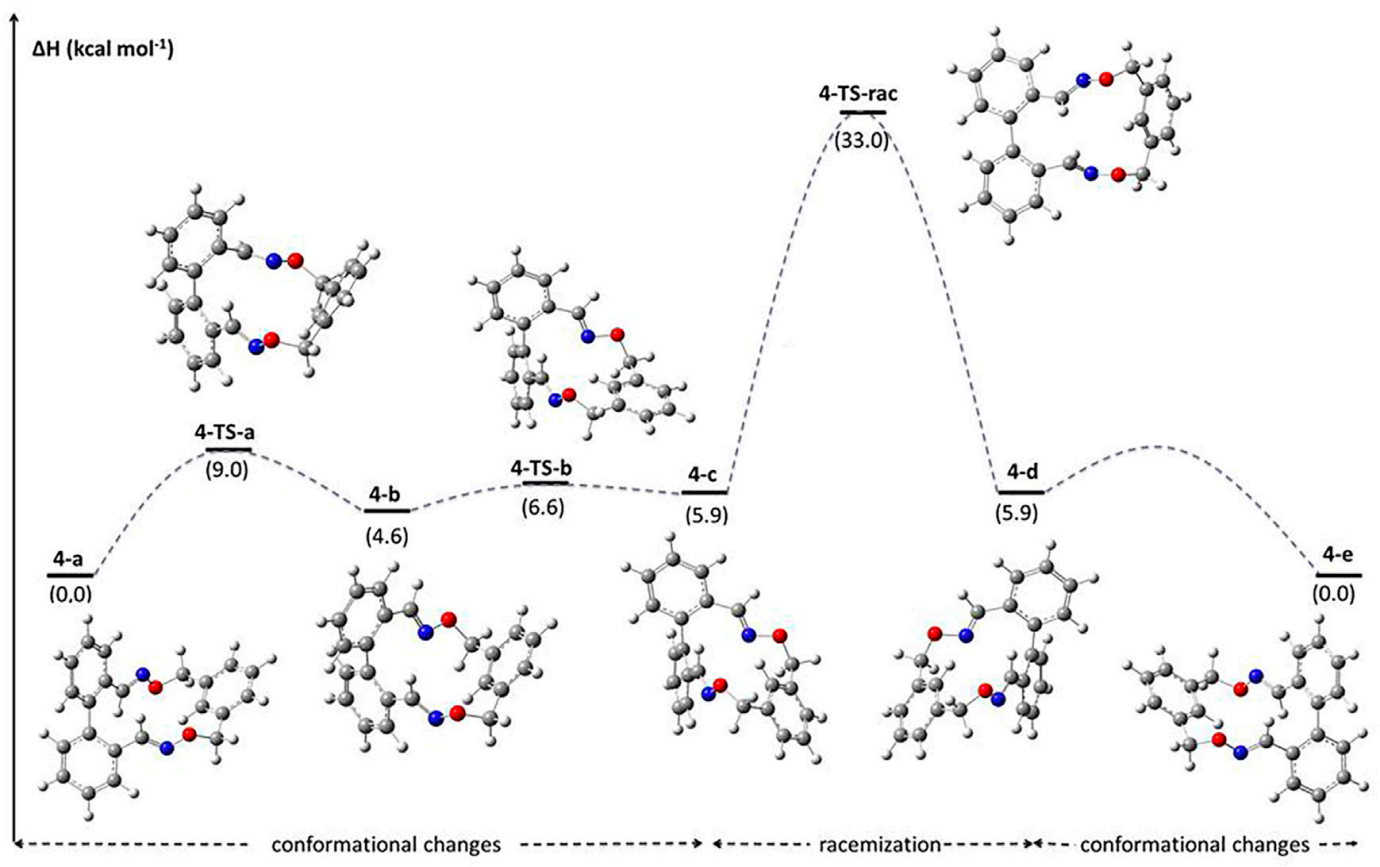
Calculated relative enthalpies and barrier values (393 K, 1 atm) for the conformers and the TSs involved in the racemization mechanism of derivative **4.**

According to the computed data, in the series of investigated oligoethyleneoxide-based banisters a slight increasing of racemization barriers is observed with the elongation of the bridge and both experimental and theoretical results point out towards a higher stability of atropisomers of macrocycle 4. The detailed racemization pathway for derivative 4 is illustrated in Figure 6, indicating the way the etheroxime units and the 1,3-bis(methylene)benzene core behave in racemization process of 4. Concerning the mechanism, firstly some conformational changes occur in the bridging moiety. Precisely, conformer 4-a transforms into 4-b through the rotation of one of the etheroxime groups around C(Ar)–C(oxime) single bond. The TS corresponding to this transformation displays a reasonable kinetic cost of *ca.* 9.0 kcal mol^−1^. Subsequently, the flipping of the phenylene ring in 4-b leads to conformer 4-c and involves a TS with a barrier of only 2.0 kcal mol^−1^, 4-TS-b. The next step involves the highest-energy TS of the whole mechanism, *i.e.* 4-TS-rac. It consists in the inversion of the helix of 4-c and the subsequent formation of species 4-d. Regarding the structural features of 4-TS-rac, it displays a biphenyl backbone which is almost planar (θ = 3.1°). Finally, 4-e is obtained from 4-d *via* several conformational changes, like those described for the 4-a to 4-c transformations.

We have shown above that the rigidity of the bridge increases the racemization barrier, but this is not the only way to achieve more stable atropisomers. Complexation of metal cations into the cavity of the macrocycles represents another alternative. Thus, binding a Li^+^ ion in the cavity of 1 increases the racemization barrier with 4.4 kcal mol^−1^ as compared to the bare macrocycle. On the other hand, Na^+^ further enhances this barrier (with 1.3 kcal mol^−1^ with respect to the 1-Li^+^ species), the calculated $\Delta H_{\mathrm{rac}}^{\neq }$ value in the case of 1-Na^+^ being 32.0 kcal mol^−1^ (Table 2).

**TABLE 2:** Calculated (PBE0-D3/Def2-TZVP) racemization enthalpies for the banisters 1–3 and their complexes.

Species	$\Delta H_{rac}^{\neq }$ (kcal mol^−1^)	Relative $\Delta H_{rac}^{\neq }$ (kcal mol^−1^)
1	26.4	0.0
1-Li^+^	30.7	4.3
1-Na^+^	32.0	5.6
1-K^+^	31.7	5.3
2	26.8	0.0
2-Li^+^	26.6	−0.2
2-Na^+^	32.8	6.0
2-K^+^	34.8	8.0
3	27.5	0.0
3-Li^+^	30.9	3.4
3-Na^+^	29.7	2.2
3-K^+^	32.9	5.4
3-Et_2_NH_2_^+^	47.1	19.6

Despite expectations, 1-K^+^ has almost the same racemization barrier as 1-Na^+^ (*i.e.* 0.3 kcal mol^−1^ lower according to the DFT data, Table 3). This behavior can be related to the contribution of the binding energies of each metal cation to the equilibrium geometry macrocycle 1 and to its’ corresponding TSs (Table 3, left side).

**TABLE 3:** Computed binding energies for complexed banisters (left) and for the complexes of their corresponding crown ethers (right).

Complexed banisters			Complexed crown ethers	
Species	Binding enthalpy (kcal mol^−1^)	Relative binding enthalpy (kcal mol^−1^)	Species	Binding enthalpy (kcal mol^−1^)
1-Li^+^-rac	−77.0	0.0		
1-Li^+^-TS-rac	−72.7	4.3	9-crown-3-Li^+^	−71.2
1-Na^+^-rac	−56.2	0.0		
1-Na^+^-TS-rac	−0.5	5.7	9-crown-3-Na^+^	−50.0
1-K^+^-rac	−41.3	0.0		
1-K^+^-TS-rac	−35.9	5.4	9-crown-3-K^+^	−37.0
2-Li^+^-rac	−87.7	0.0	12-crown-4-Li^+^	−86.0
2-Li^+^-TS-rac	−87.9	−0.2		
2-Na^+^-rac	−68.1	0.0		
2-Na^+^-TS-rac	−62.2	5.9	12-crown-4-Na^+^	−61.1
2-K^+^-rac	−52.7	0.0		
2-K^+^-TS-rac	−44.7	8.0	12-crown-4-K^+^	−45.2
3-Li^+^-rac	−96.1	0.0	15-crown-5-Li^+^	−99.5
3-Li^+^-TS-rac	−92.7	3.4		
3-Na^+^-rac	−76.0	0.0	15-crown-5-Na^+^	−78.2
3-Na^+^-TS-rac	−73.8	2.2		
3-K^+^-rac	−60.2	0.0	15-crown-5-K^+^	−59.7
3-K^+^-TS-rac	−54.8	5.4		

Firstly, the binding energy in TSs displays lower values than the one computed for the equilibrium geometries. This can stand as an explanation for the increasing of the racemization barrier of macrocycle 1 upon complexation with the metal ions. Moreover, the difference between calculated $\Delta H_{\mathrm{rac}}^{\neq }$ values of complexed and uncomplexed macrocycle (from Table 2) matches the cation-induced destabilization energy of TS relative to GS of macrocycle-cation complex (relative binding enthalpy values in Table 3).

**FIGURE 7:**
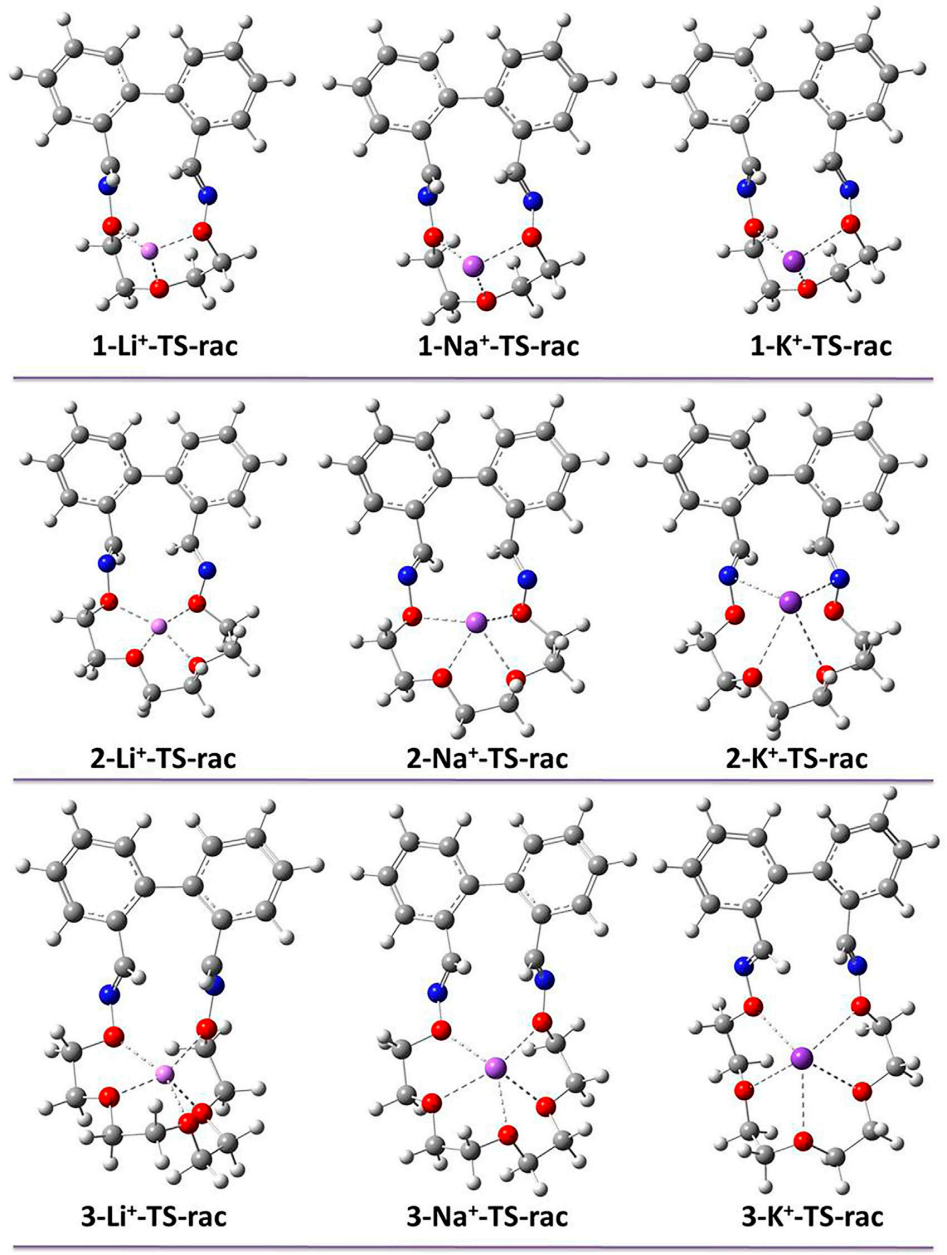
Optimized TS geometries of complexed macrocycles 1–3.

Secondly, binding energies decrease with increasing ionic radius of cations. Thus, taking a closer look at the optimized TS geometries (Figure 7), Li^+^ is most suitable for the small cavity of 1, as shown by GS geometries (Figure 7 and Supplementary Figure S2). By the contrary, the bigger Na^+^ and K^+^ are placed above the cavity of the macrocycle and not inside, thus the complexed species behave similarly in the racemization process.

In the case of the complexed macrocycle 2, the racemization barriers increase with the increasing of the ionic radius of the hosted cation, from 26.6 kcal mol^−1^ (in the case of 2-Li^+^) to 34.8 kcal mol^−1^ (for 2-K^+^) (Table 2). The racemization barrier of 2-Li^+^ is unexpected, as it displays 0.2 kcal mol^−1^ lower $\Delta H_{\mathrm{rac}}^{\neq }$ value than that of the bare macrocycle 2. In this case, DFT calculations suggest that the TS and the GS have almost the same affinity for the Li^+^ ion. In other words, both GS and TS are stabilized by Li^+^ with almost the same energy and, consequently, the racemization barrier does not surpass that of the uncomplexed macrocycle, as in the case of all the other complexes.

Concerning the metal complexes of macrocycle 3, a 3.4 kcal mol^−1^ extra stabilization of the ground state relative to transition state, brought by the lithium ion leads to a higher racemization barrier by additional 3.4 kcal mol^−1^, while Na^+^ increases the $\Delta H_{\mathrm{rac}}^{\neq }$ value with 2.1 kcal mol^−1^. Potassium is a more suitable cation for the cavity of macrocycle 3 than for that of macrocycle 2, so 3-K^+^ has with almost 2.0 kcal mol^−1^ a lower racemization barrier than 2-K^+^.

**FIGURE 8:**
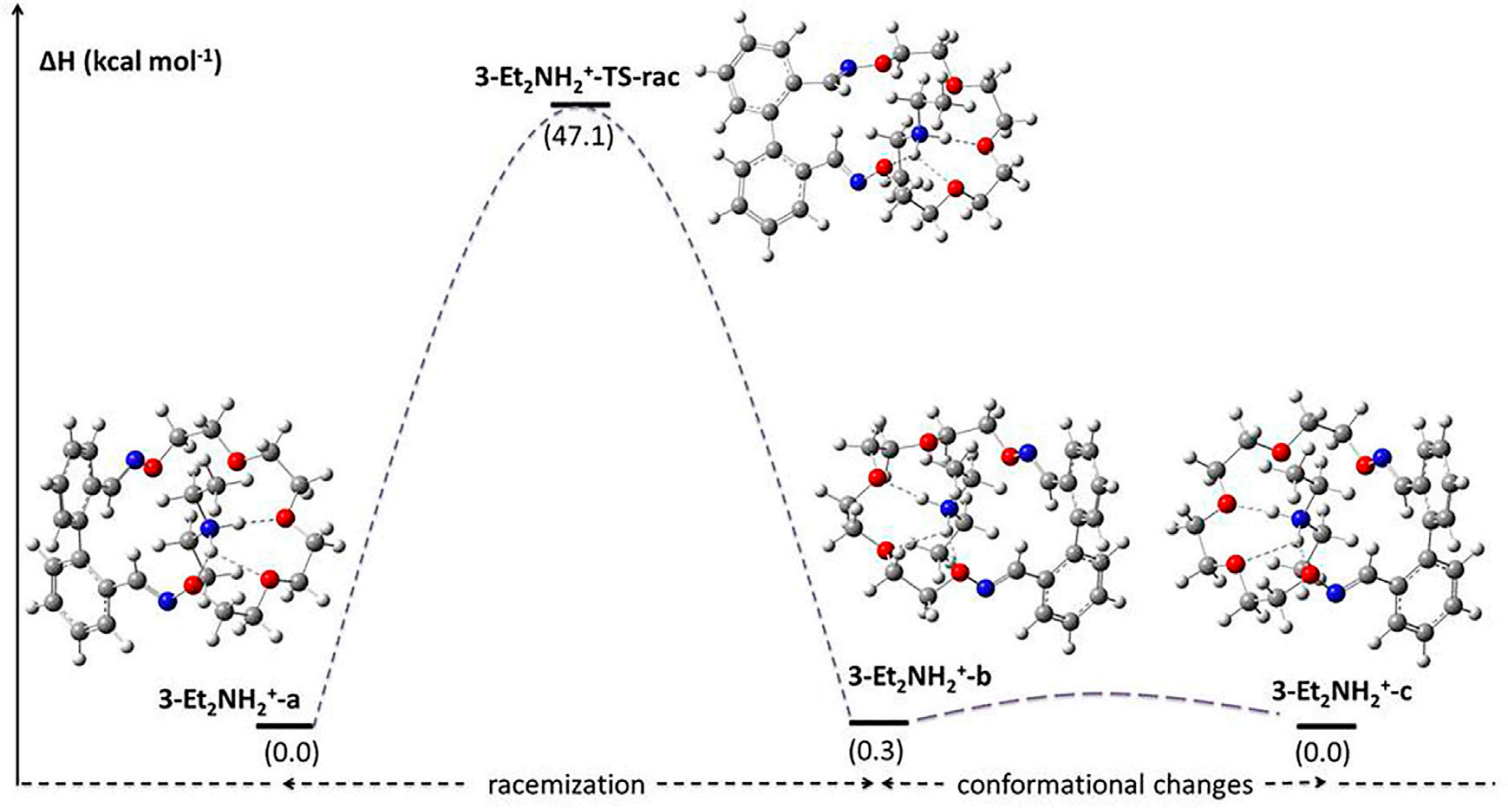
DFT calculated relative enthalpies and barrier values (353 K, 1 atm) for the racemization mechanism of 3-Et_2_NH_2_^+^.

A much higher value of the racemization barrier was obtained for the pseudo[2]rotaxane 3-Et_2_NH_2_^+^, namely, 47.1 kcal mol^−1^. This value is higher even than the one reported by Kimura *et al* for urea-rotaxane (45 kcal mol^−1^ in *o*-dichlorobenzene, Kimura et al., 2020). A proposed racemization mechanism for the 3-Et_2_NH_2_^+^ species is depicted in Figure 8. Due to the rigidity of ether oxime groups, the presence of the rod-like diethylammonium ion in the cavity enhances the strain energy of the TS in which the biphenyl backbone is almost planar.

An attractive idea was to compare the binding enthalpies obtained for complexed macrocycles 1–3 with those found for the corresponding complexed crown ethers having a similar number of oligoethyleneoxide units (Table 3, right). In this respect, the interaction energies were computed for the 9-crown-3, 12-crown-4 and 15-crown-5 species upon complexation with Li^+^, Na^+^ and K^+^. In addition, the theoretical method employed herein (*i.e.* PBE0-D3/Def2-TZVP) reveals satisfactory agreement with previous experimental and theoretical binding energy studies performed on complexed 12-crown-4 species (experimental data (in kcal mol^−1^): −90 ± 12 for 12-crown-4-Li^+^, −61 ± 3 for 12-crown-4-Na^+^, −46 ± 3 for 12-crown-4-K^+^; Hill et al., 1998). In the TS, the torsion angles are almost zero and the ethyleneoxide bridge of 1 hosts cations like a 9-crown-3 does, while in the GS the cavity enlarges and the banister binds cations more efficiently. In the case of complexed macrocycle 2, binding energies in both 2-Li^+^ and 2-Li^+^-TS-rac are comparable with that for 12-crown-4-Li^+^ complex, in all cases Li^+^ being placed in the middle of the cavity (Figures 1, 7 and Supplementary Figures S15, S16 for complexed crown ethers). Instead, sodium and potassium cations are hosted more efficiently by the GS of banister 2 than by 12-crown-4. By contrast with previous correlations, the binding energies in complexed 15-crown-5 correlate with those found in the GSs, not in the TSs, of complexed macrocycle 3.

## Conclusion

In conclusion, we have conducted a thorough computational study on a series of axially chiral oxime ether-containing banister-type macrocycles 1–4. The calculated values for the barrier to rotation around the *sp* ^*2*^ *–sp* ^*2*^ bond in the *o,o’*-bridged biphenyl unit in the bare macrocycles are in good agreement with the experimental ones, highlighting the higher stability of the atropisomers of the more rigid macrocycle 4 (∼6 kcal mol^−1^) over those with more flexible oligoethylene oxide bridges 1–3. Solid-state data collected by single crystal X-Ray diffractometry of 4 were corroborated with computed data and the contribution of main supramolecular associations in the lattice to the stabilization of the crystal structure could be estimated. Following the hypothesis that complexation of macrocycles with cations would further augment the stability of atropisomers, our computational results showed an increase in the racemization barrier of up to 8 kcal mol^−1^ in 1–3 complexed with alkali metal ions and an impressive 19.6 kcal mol^−1^ in the case of the pseudo[2]rotaxane resulted from complexation of macrocyle 3 with the rod-like diethylammonium cation. A correlation between the size of the macrocycle and the preferred metal ion was observed and compared to the well-documented host-guest behavior of crown ethers of similar oligoethyleneoxy bridge size. Further studies on applying these results in the design and synthesis of structurally more sophisticated separable banister atropisomers and their complexes are in progress.

## Data Availability

The datasets presented in this study can be found in online repositories. The names of the repository/repositories and accession number(s) can be found below: The Cambridge Crystallographic Data Centre (CCDC) under the number CCDC 2100390.
