# Supplementary material for: Solid-State and Theoretical Investigations of Some Banister-Type Macrocycles with 2,2’-Aldoxime-1,1’-Biphenyl Units
Source: Front Chem. 2021 Oct 6;9:750418. doi: 10.3389/fchem.2021.750418 (PMC8527037; doi:10.3389/fchem.2021.750418)
Supplement: Supplementary file 1 [file DataSheet1.pdf]

Supplementary Material

Solid-State and Theoretical Investigations of some Banister-Type Macrocycles with 2,2'-Aldoxime-1,1'-Biphenyl Units

Ioan Stroia, Ionuț-Tudor Moraru, Maria Miclăuş, Ion Grosu, Claudia Lar, Ioana Georgeta Grosu, Anamaria Terec

Contents

Single-crystal X-ray diffraction measurement........1

Supplementary Figure S1: Supramolecular associations occurring within species **4** and their DFT estimated interaction energies.. 2

Supplementary Scheme S1: General homodesmotic reactions used to compute the strain energies in macrocycles **1-4**........3

Supplementary Table S2: Strain energies for macrocycles **1-4** based on homodesmotic reactions....3

Supplementary Table S3: DFT (PBE0-D3/Def2-TZVP and B3LYP-D3/Def2-TZVP) and experimental racemization barriers determined for banisters **1-4**. 3

Supplementary Figure S2: Top view of the optimized molecular structures of macrocycles **1-3** and their alkali metal ion (Li^+, Na^+, K^+) complexes........4

Supplementary Figure 3: Theoretical relative enthalpies profile (353 K, 1 atm) for the racemization mechanism of **1**........5

Supplementary Figure 4: Theoretical relative enthalpies profile (353 K, 1 atm) for the racemization mechanism of **2**........5

Supplementary Figure 5: Theoretical relative enthalpies profile (353 K, 1 atm) for the racemization mechanism of **3**........6

Supplementary Figure 6: Theoretical relative enthalpies profile (353 K, 1 atm) for the racemization mechanism of **1-Li$^+$**. 6

Supplementary Figure 7: Theoretical relative enthalpies profile (353 K, 1 atm) for the racemization mechanism of **1-Na$^+$**........7

Supplementary Figure 8: Theoretical relative enthalpies profile (353 K, 1 atm) for the racemization mechanism of **1-K$^+$**........7

Supplementary Figure 9: Theoretical relative enthalpies profile (353 K, 1 atm) for the racemization mechanism of **2-Li**$^+$. 8

Supplementary Figure 10: Theoretical relative enthalpies profile (353 K, 1 atm) for the racemization mechanism of **2-Na**$^+$. 8

Supplementary Figure 11: Theoretical relative enthalpies profile (353 K, 1 atm) for the racemization mechanism of **2-K**$^+$. 9

Supplementary Figure 12: Theoretical relative enthalpies profile (353 K, 1 atm) for the racemization mechanism of **3-Li**$^+$**.** 9

Supplementary Figure 13: Theoretical relative enthalpies profile (353 K, 1 atm) for the racemization mechanism of **3-Na**$^+$. 10

Supplementary Figure 14: Theoretical relative enthalpies profile (353 K, 1 atm) for the racemization mechanism of **3-K**$^+$. 10

Supplementary Figure 15: Top view of optimized ground states geometries of complexed **9-crown-3**, **12-crown-4** and **15-crown-5**. 11

Supplementary Figure 16: Side view of optimized ground states geometries of complexed **9-crown-3**, **12-crown-4** and **15-crown-5**. 11

DFT coordinates 12

Single-crystal X-ray diffraction measurement

Single-crystal diffraction data for **4** were collected on Oxford Diffraction SuperNova dual wavelength diffractometer exhibiting an operating mirror monochromated MoKα radiation mode (λ = 0.71073Å). X-ray data collection was monitored and all the data were corrected for Lorentzian, polarization and absorption effects using CrysAlisPro program [Agilent Technologies, CrysAlis PRO, Yarnton Oxfordshire, England: Agilent Technologies, 2010]. Olex2 program was used for the crystal structure solution and refinement [Dolomanov, O.V., Bourhis, L.J., Gildea, R.J, Howard, J.A.K. &Puschmann, H. (2009), J. Appl. Cryst. 42, 339-341], SHELXS [Sheldrick, G.M. (2008). A64, 112-122] were used for structure solutions. SHELXL was used for full matrix least-squares refinement on F2 [Sheldrick, G.M. (2015). Acta Cryst. C71, 3-8].

CCDC 2100390 contains the supplementary crystallographic data for this paper. The data can be obtained free of charge from The Cambridge Crystallographic Data Centre via www.ccdc.cam.ac.uk/structures.

Identification code	**4**
CCDC number	2100390
Empirical formula	$C_{22}H_{18}N_2O_2$
Formula weight	342.38
μ (Mo Kα)/mm^{-1}	0.71073
Temperature/K	293(2)
Crystal system	monoclinic
Space group	P 2_1/n
a/Å	13.0101(6)
b/Å	9.8134(4)
c/Å	14.5485(7)
α/°	90.00
β/°	104.166(5)
γ/°	90.00
Volume/Å^3	1800.97(15)
Z	4
Density (calculated) /g cm^{-3}	1.263
Absorption coefficient /mm^{-1}	0.082
F(000)	720

Reflections collected	8753
Data/restraints/parameters	4103/0/235
Goodness-of-fit on F^2	1.059
Final R indexes [I>=2σ (I)]	R_1= 0.0548, wR_2= 0.1568
Final R indexes [all data]	R_1= 0.0843, wR_2= 0.1871
Largest diff. peak/hole / e $Å^{-3}$	0.19/-0.20

Supplementary Table S1: Crystallographic data for **4**

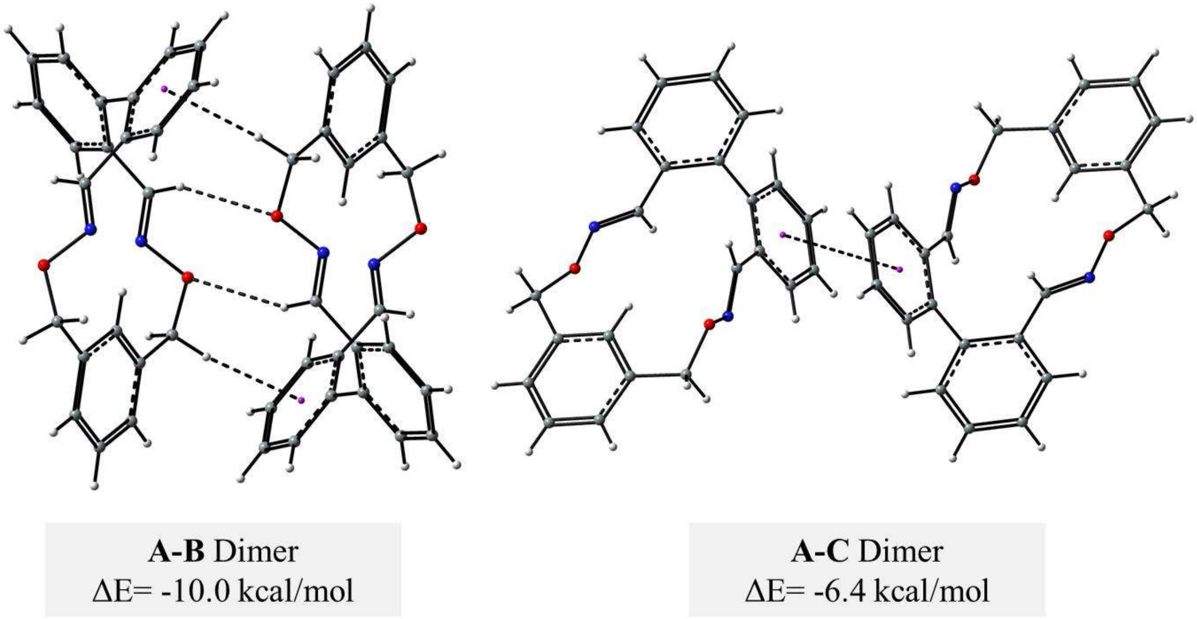

Supplementary Figure S1: Supramolecular associations occurring within species **4** and their DFT estimated interaction energies. **A–B** dimer case (left side), displaying reciprocal C–H•••O and C–H•••π interactions. Computed interaction energy is of ca. 10.0 kcal/mol (ΔE°/dioxime molecule = 5 kcal mol-1). **A–C** dimer case (right side), revealing π ••π interactions. Computed interaction energy is of ca. 6.4 kcal/mol (ΔE°/dioxime molecule = 3.2 kcal mol-1). Interaction energies were estimated using the formula: ΔE= E (Dimer) – 2 x E (Monomer). The coordinates of the atoms (for both dimers and monomers) were imported from crystallographic data without performing optimization of the molecular geometries (i.e. single-point calculations only). All these analyses were carried out within the framework of the DFT method, employing the D3 dispersion-corrected version of the PBE0 hybrid functional and the valence triple-zeta Def2-TZVP basis set.

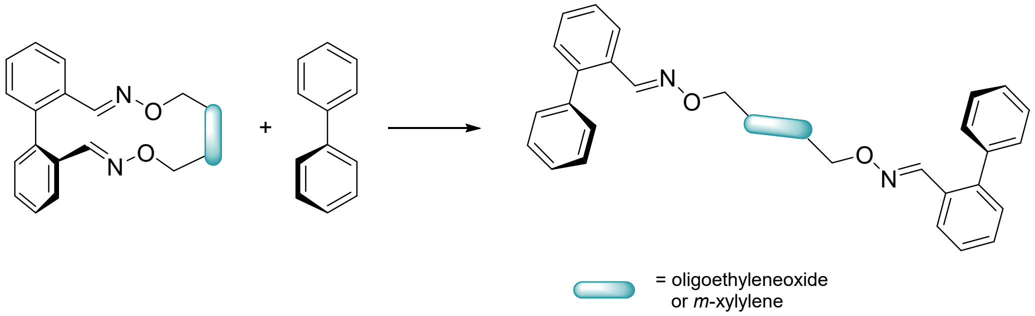

Supplementary Scheme S1: General homodesmotic reactions used to compute the strain energies in macrocycles **1-4.** All structures were optimized at the same level of theory (PBE0-D3/Def2-TZVP) and at default temperature (298 K)

Macrocycle	Strain energy (-ΔH, kcal mol^{-1})
**1**	0.5
**2**	-1.4
**3**	0.5
**4**	-0.6

Supplementary Table S2: Strain energies for macrocycles **1-4** based on homodesmotic reactions. Strain energies were calculated using the formula: ΔH= H (product) – H (macrocycle) – H (biphenyl).

Name	Racemization barrier (kcal mol^{-1})		
	Theoretical		Experimental
	PBE0-D3	B3LYP-D3	
**1**	26.4	26.8	25.0
**2**	26.8	26.4	24.8
**3**	27.5	27.6	24.7
**4**	33.0	33.7	31.7

Supplementary Table S3: DFT (PBE0-D3/Def2-TZVP and B3LYP-D3/Def2-TZVP) and experimental racemization barriers determined for banisters **1-4**.

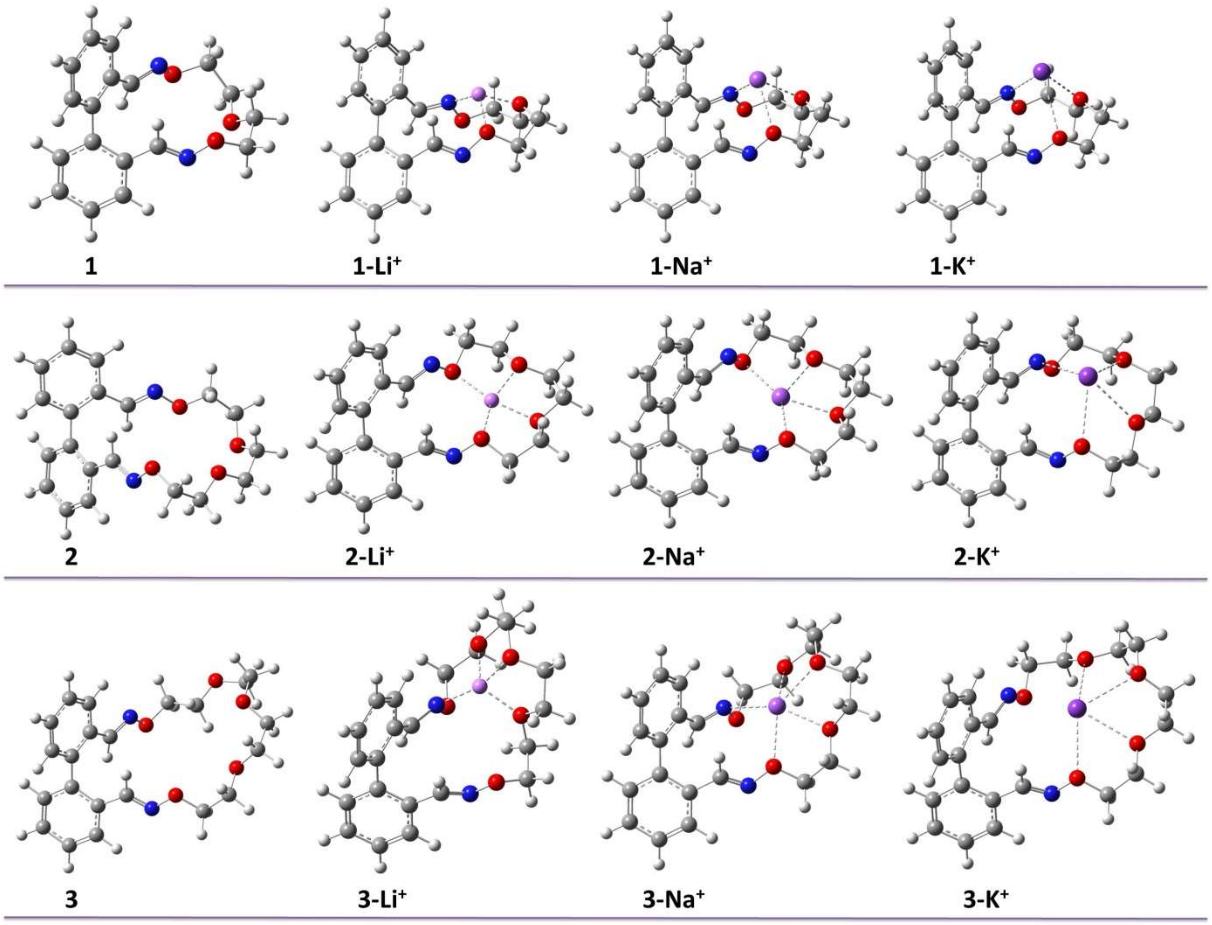

Supplementary Figure S2: Top view of the optimized molecular structures of macrocycles **1-3** and their alkali metal ion (Li^+, Na^+, K^+) complexes.

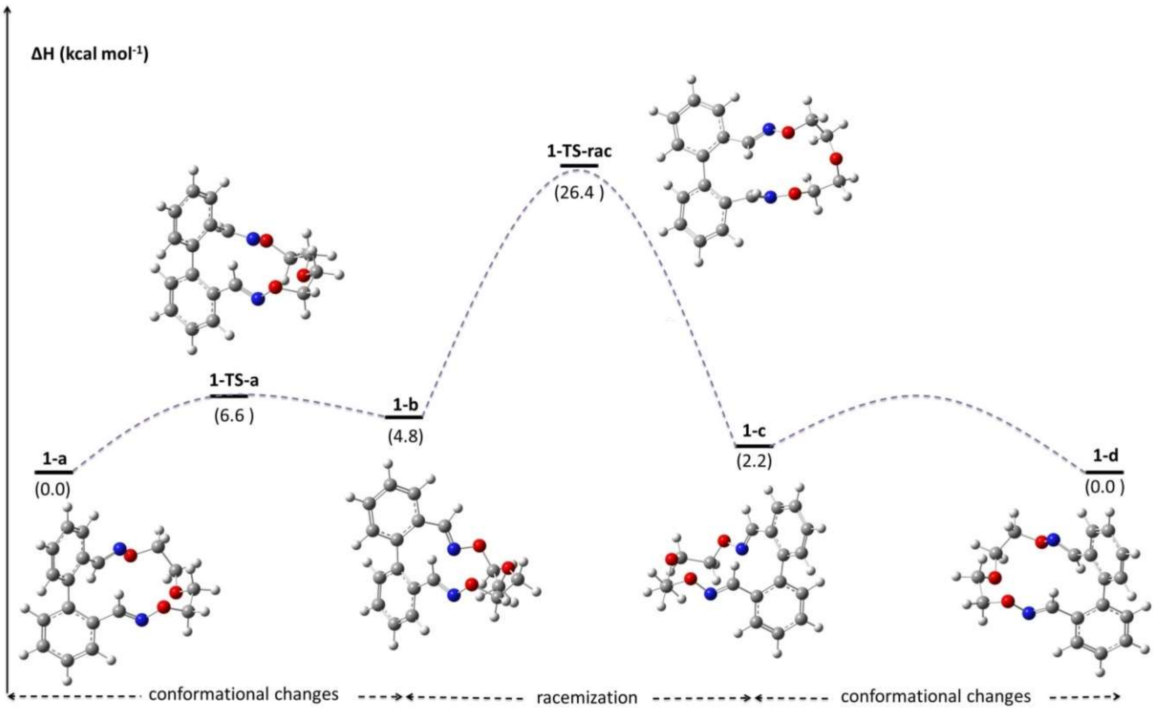

Supplementary Figure S3: Theoretical relative enthalpies profile (353 K, 1 atm) for the racemization mechanism of **1**.

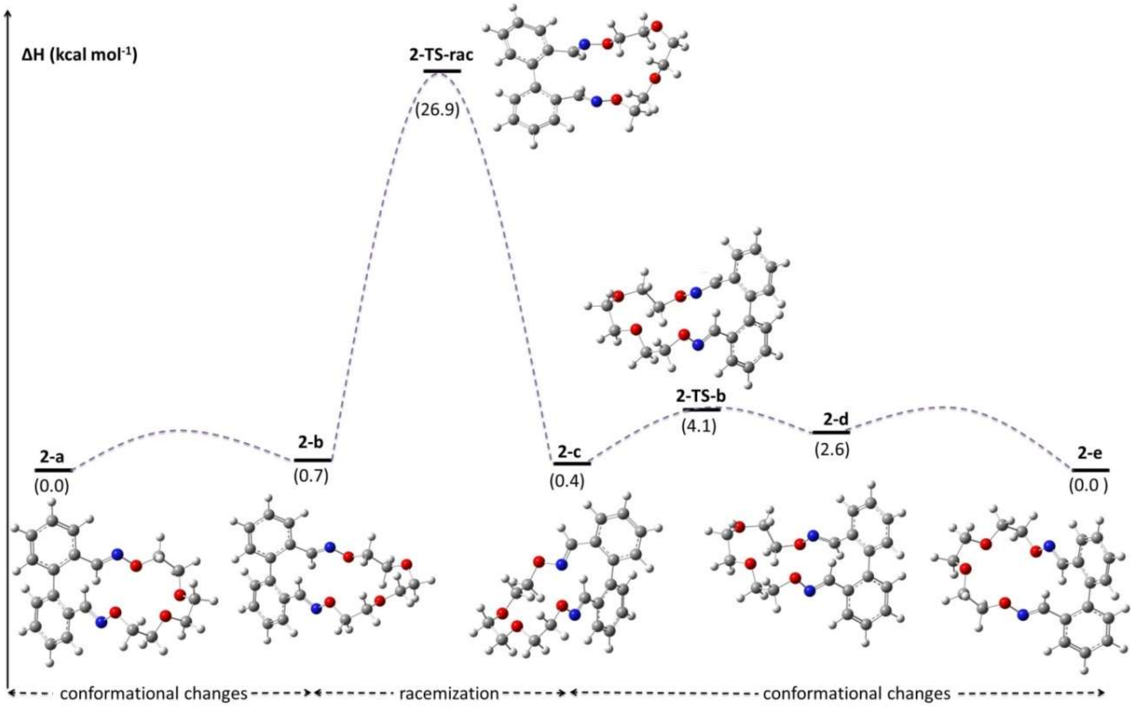

Supplementary Figure S4: Theoretical relative enthalpies profile (353 K, 1 atm) for the racemization mechanism of **2**.

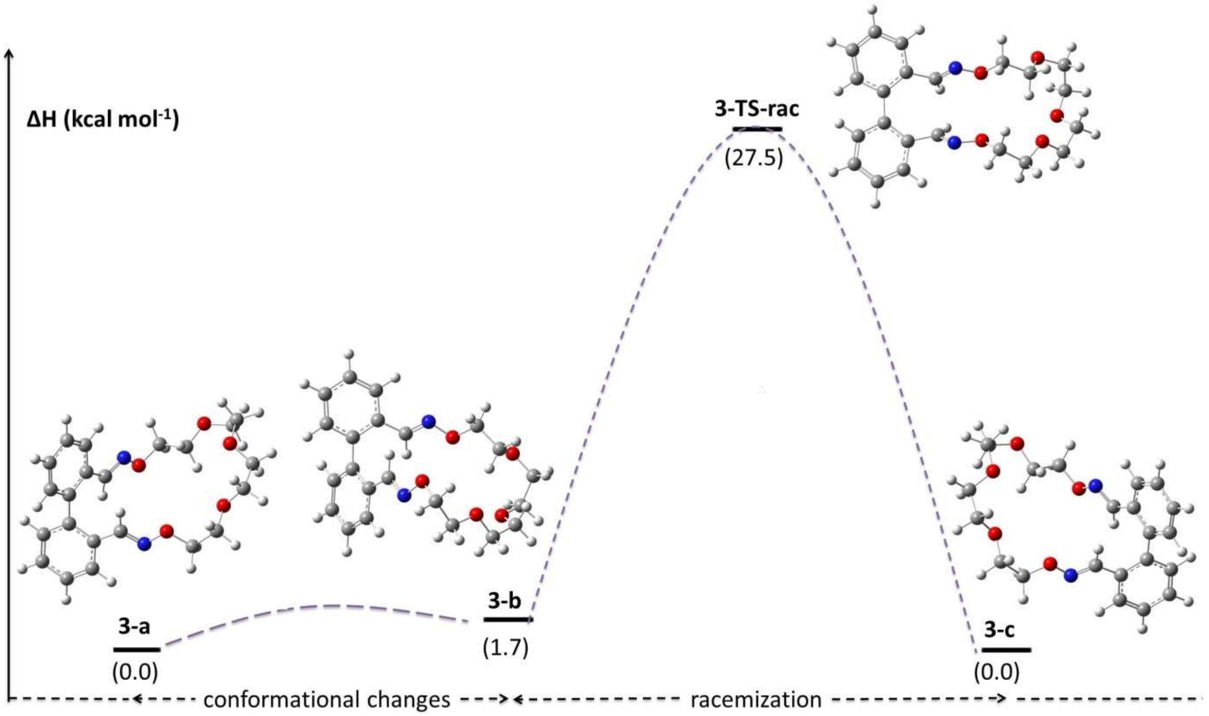

Supplementary Figure S5: Theoretical relative enthalpies profile (353 K, 1 atm) for the racemization mechanism of **3**.

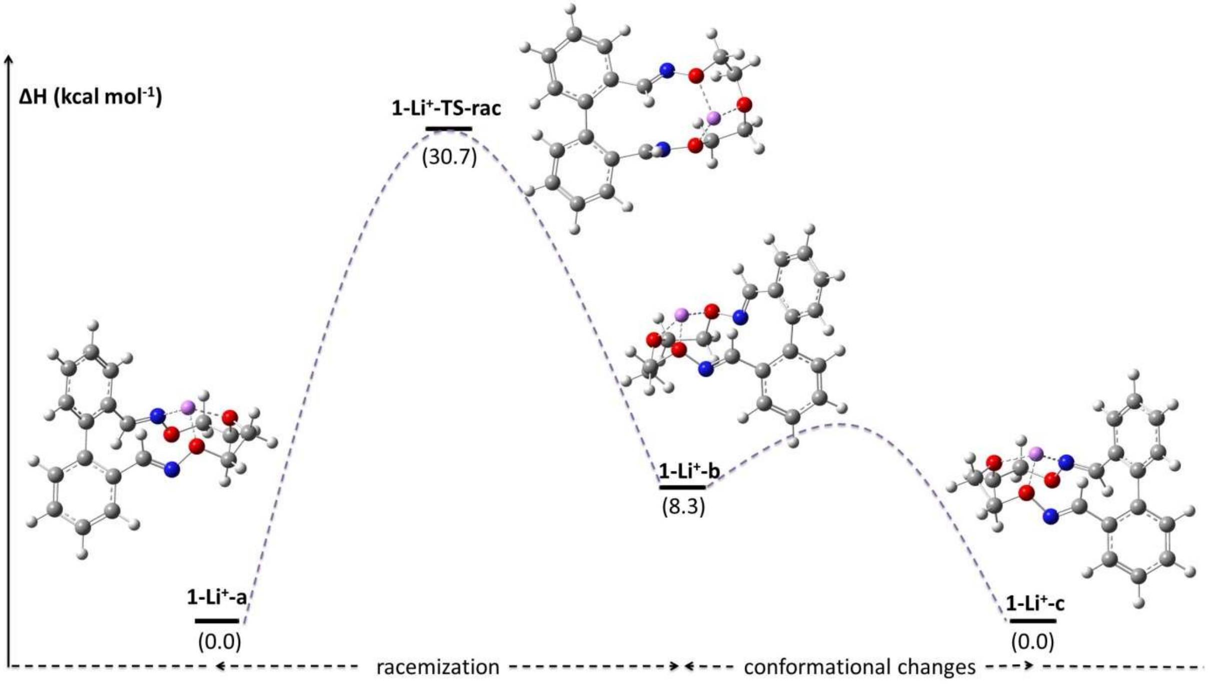

Supplementary Figure S6: Theoretical relative enthalpies profile (353 K, 1 atm) for the racemization mechanism of **1-Li**$^+$.

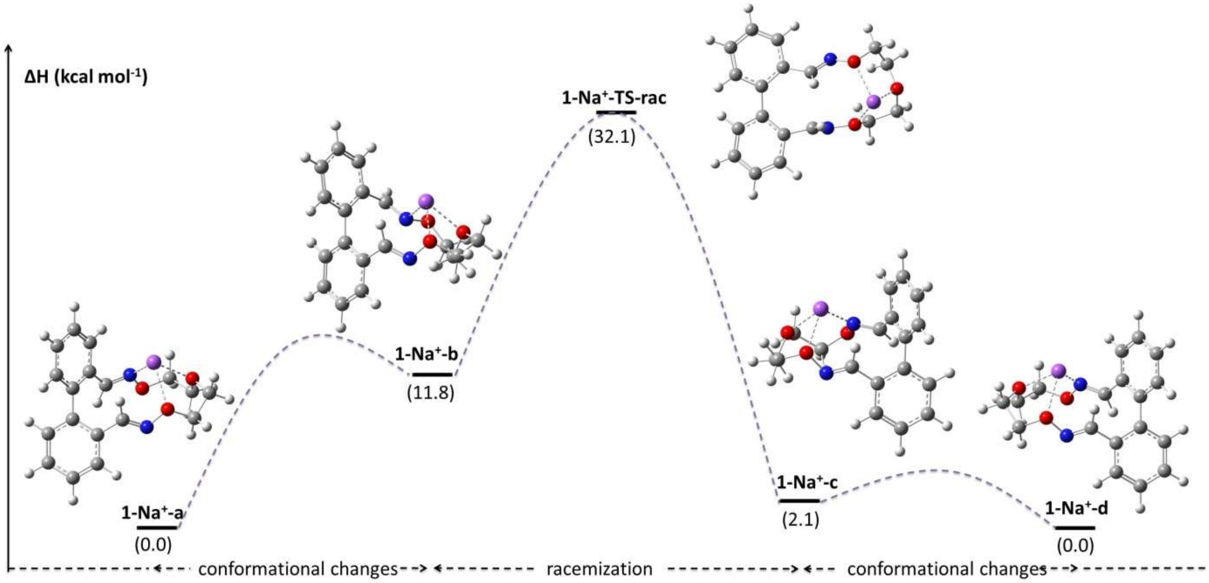

Supplementary Figure S7: Theoretical relative enthalpies profile (353 K, 1 atm) for the racemization mechanism of **1-Na$^+$**.

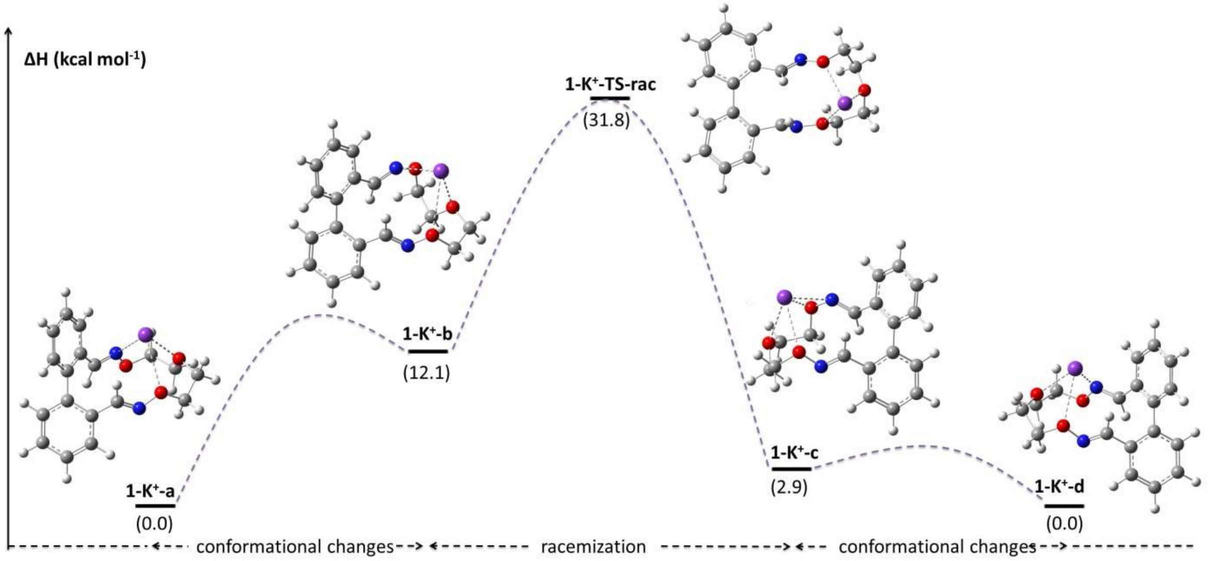

Supplementary Figure S8: Theoretical relative enthalpies profile (353 K, 1 atm) for the racemization mechanism of **1-K$^+$**.

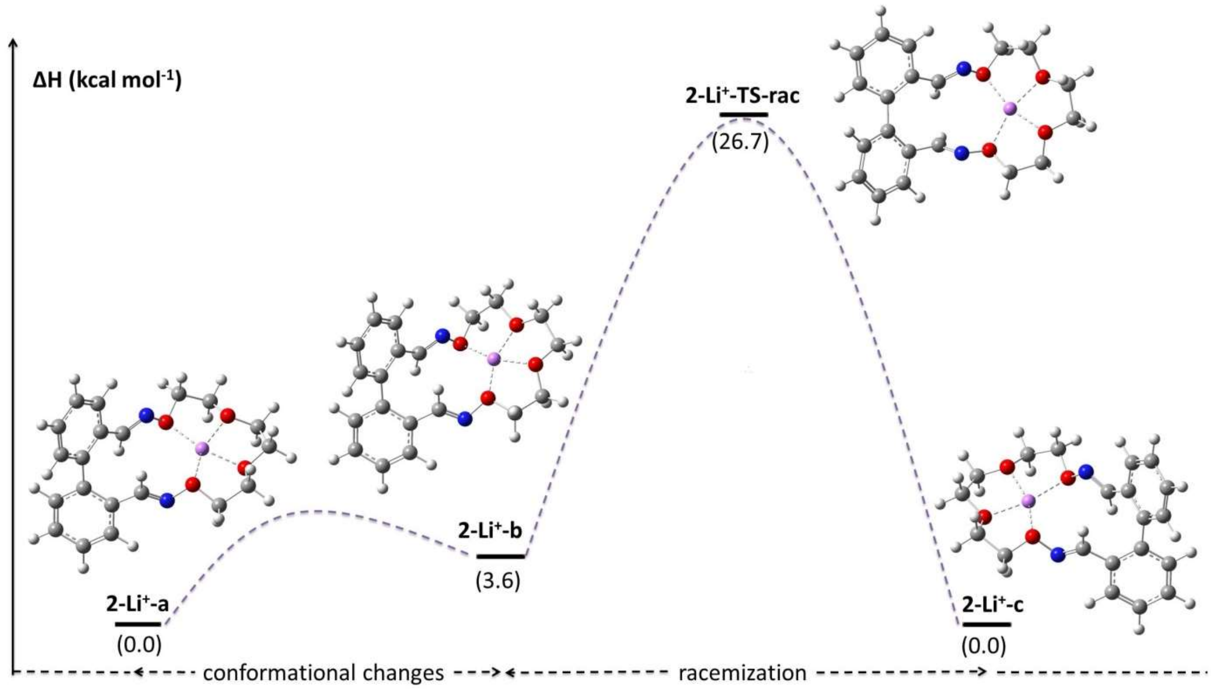

Supplementary Figure S9: Theoretical relative enthalpies profile (353 K, 1 atm) for the racemization mechanism of **2-Li$^+$**.

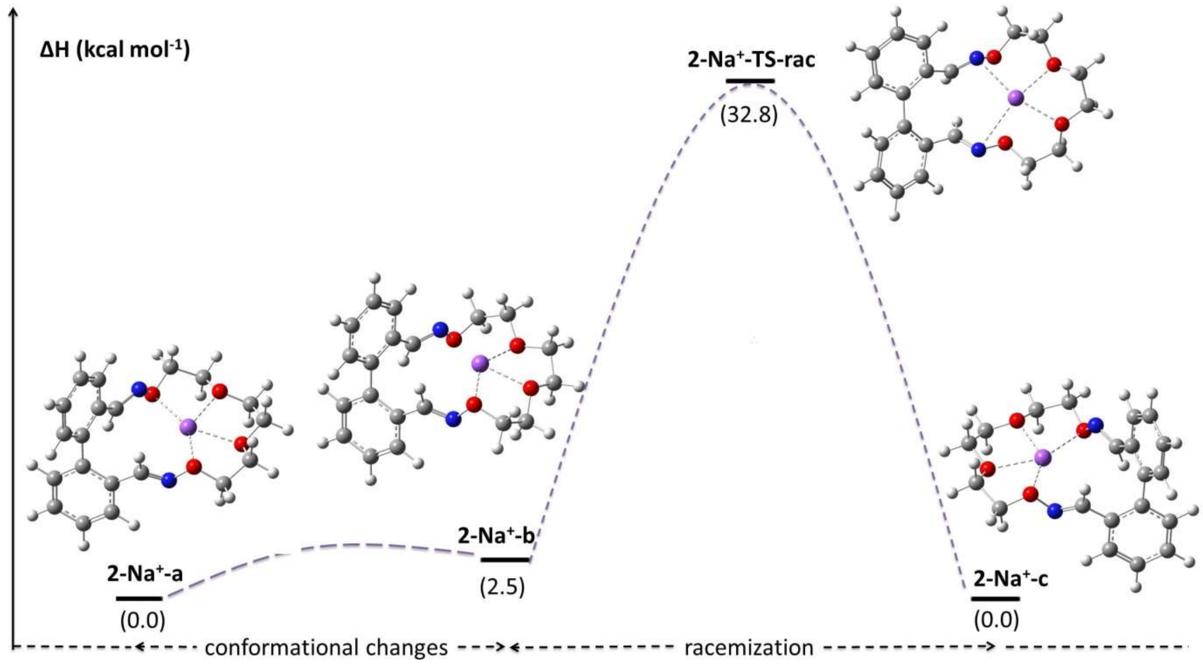

Supplementary Figure S10: Theoretical relative enthalpies profile (353 K, 1 atm) for the racemization mechanism of **2-Na$^+$**.

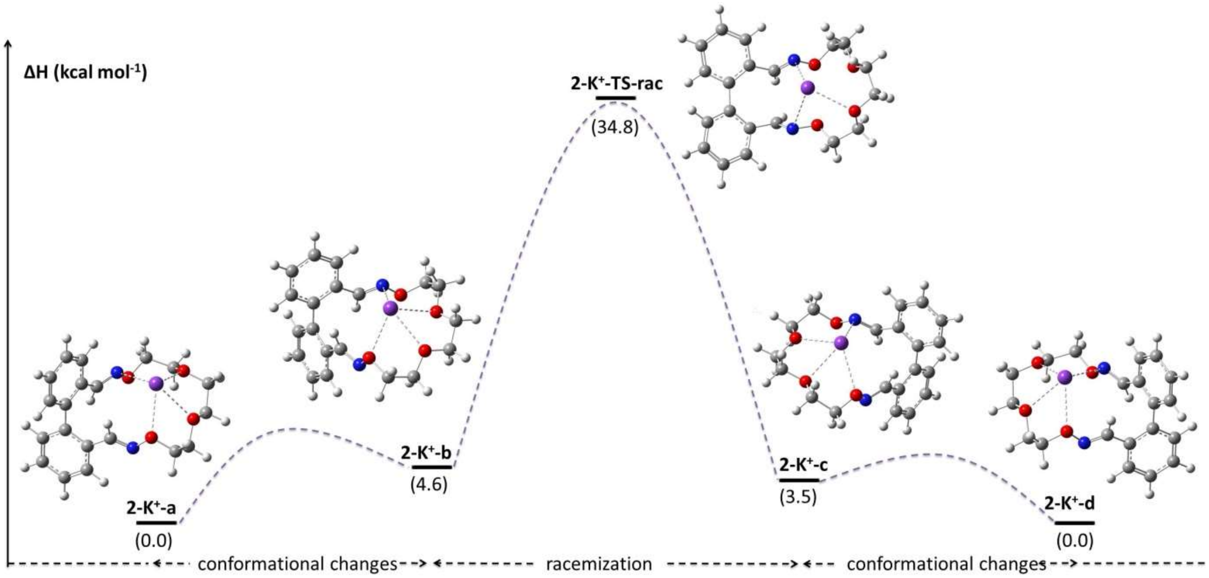

Supplementary Figure S11: Theoretical relative enthalpies profile (353 K, 1 atm) for the racemization mechanism of **2-K**$^+$.

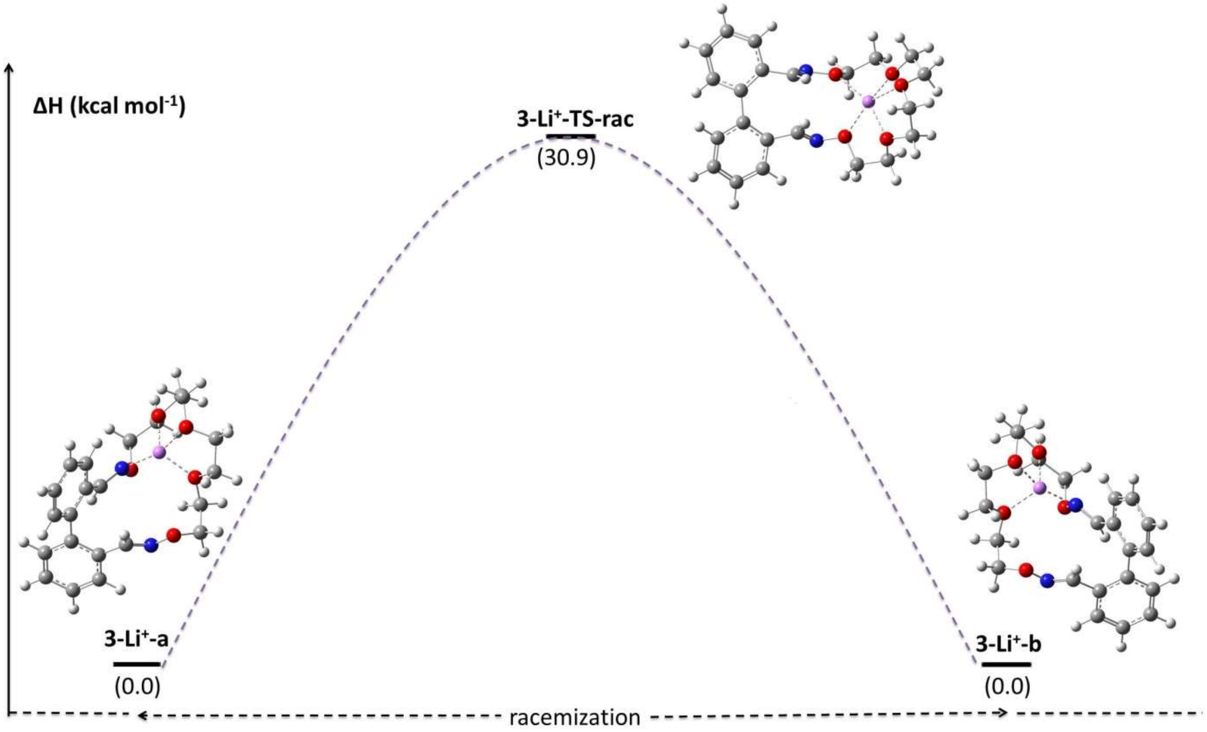

Supplementary Figure S12: Theoretical relative enthalpies profile (353 K, 1 atm) for the racemization mechanism of **3-Li**$^+$**.**

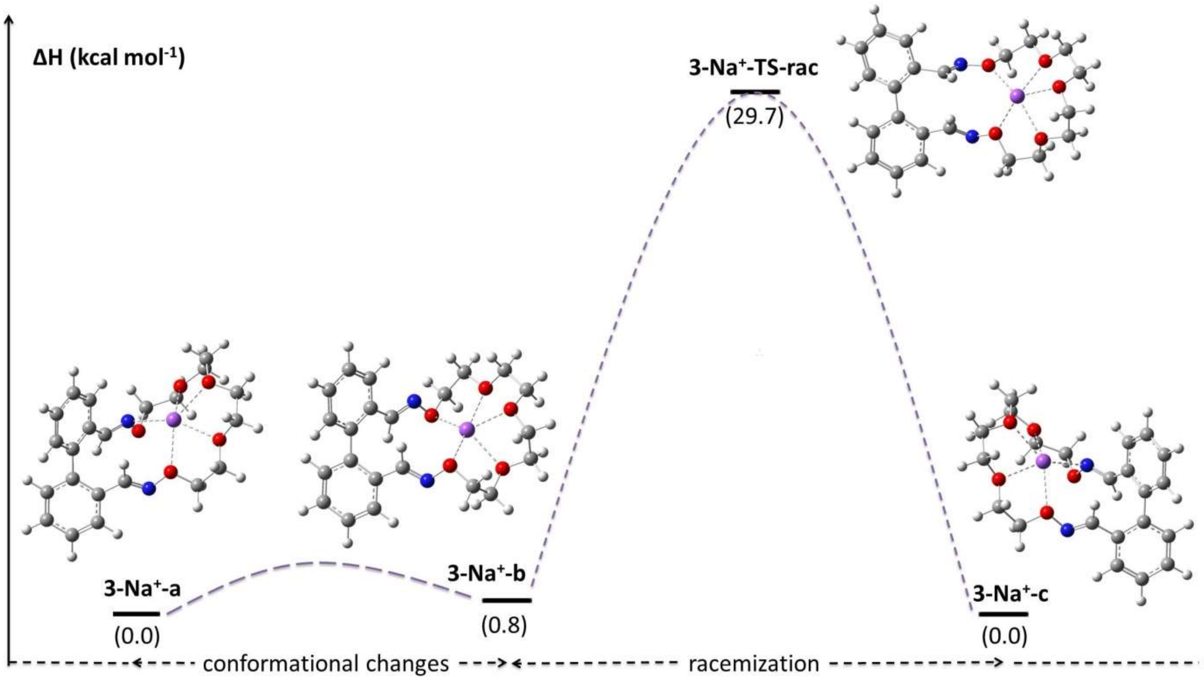

Supplementary Figure S13: Theoretical relative enthalpies profile (353 K, 1 atm) for the racemization mechanism of **3-Na⁺.**

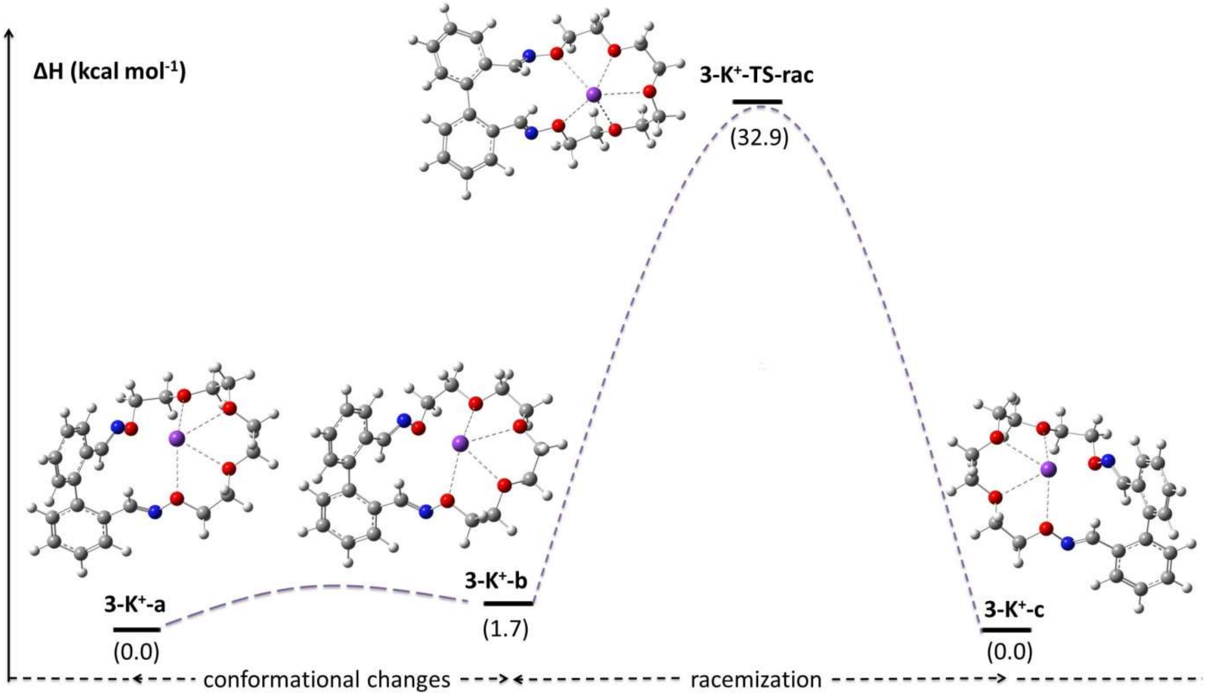

Supplementary Figure S14: Theoretical relative enthalpies profile (353 K, 1 atm) for the racemization mechanism of **3-K⁺.**

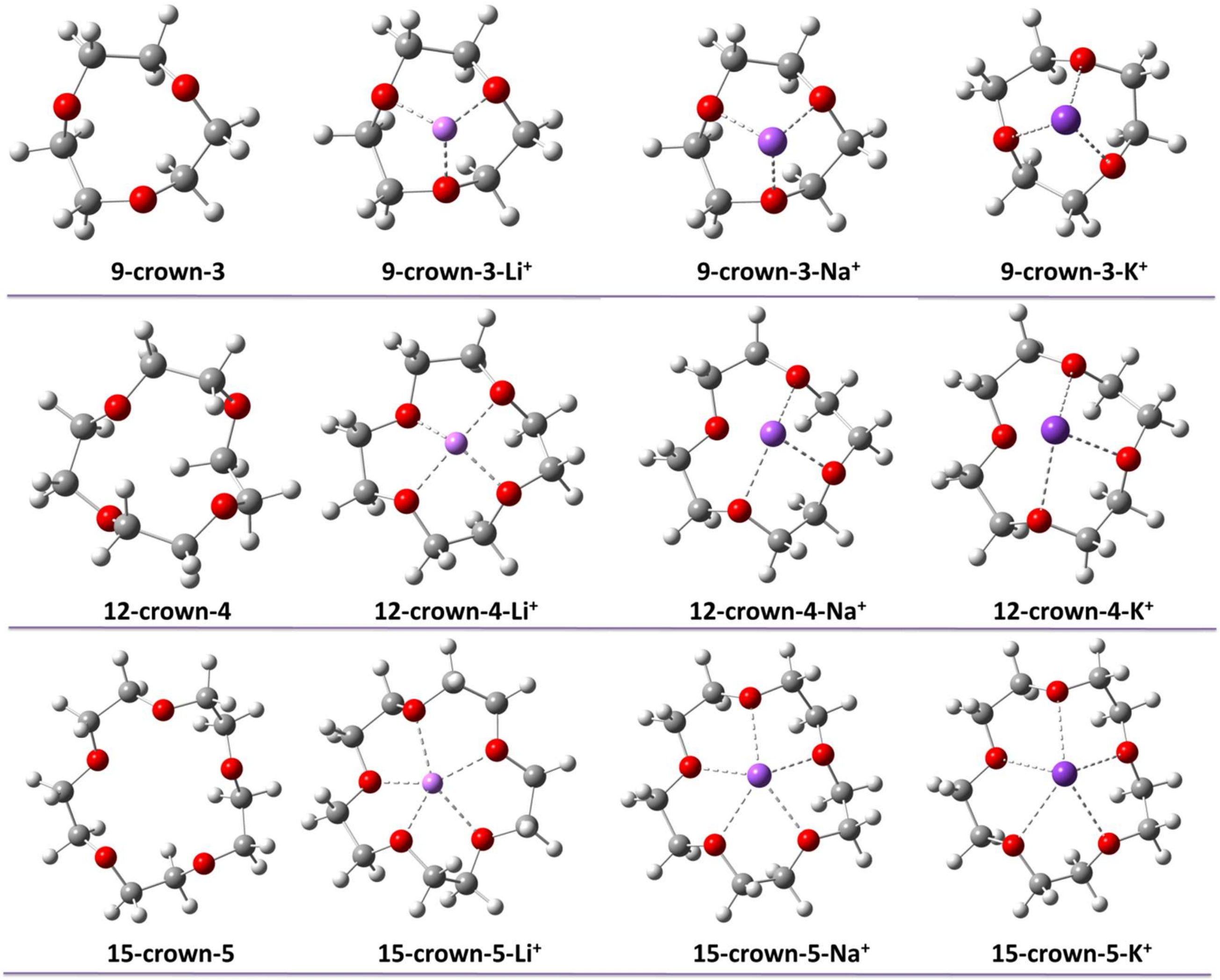

Supplementary Figure S15: Top view of optimized ground state geometries of complexed **9-crown-3**, **12-crown-4** and **15-crown-5**.

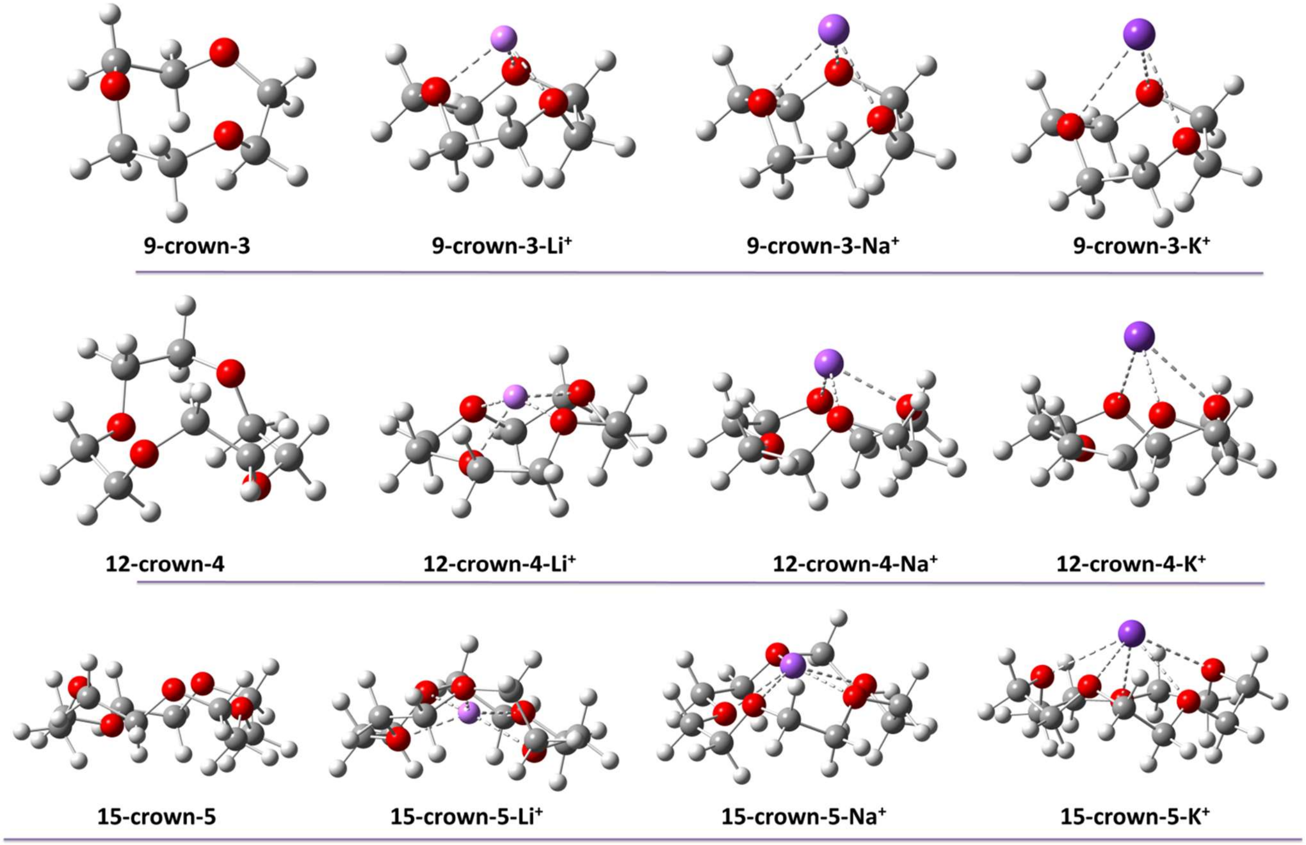

Supplementary Figure S16: Side view of optimized ground state geometries of complexed **9-crown-3**, **12-crown-4** and **15-crown-5**.

DFT coordinates

1-a

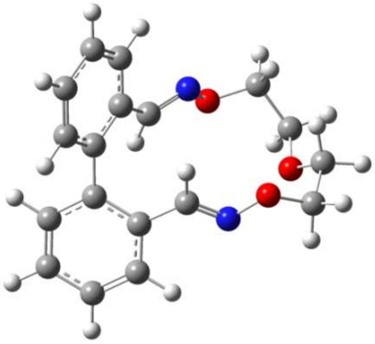

H (353 K, 1 atm) = -1030.651589 a.u.

H	0.39592100	-0.07942300	0.01108000
C	0.29710800	-0.08156600	1.09053600
C	0.04101100	-0.09831900	3.87318500
C	-0.31586500	-1.14557000	1.72334700
C	0.77044800	0.98951100	1.83807800
C	0.63835300	0.97611300	3.21559100
C	-0.45634600	-1.17109100	3.11249700

H	-0.71127900	-1.97630900	1.15168300
H	1.24541700	1.83185100	1.34831600
H	1.02367900	1.79927100	3.80731900
C	-0.04860000	-0.07532600	5.35192100
C	-0.31763100	-0.00187300	8.13362300
C	0.52935300	-1.08101700	6.14504000
C	-0.73278900	0.96506200	5.97730800
C	-0.87037700	1.00595300	7.35454700
C	0.38184900	-1.03126900	7.53159500
H	-1.18290200	1.73655300	5.36221000
H	-1.41287500	1.82127600	7.81928600
H	0.84244200	-1.81023500	8.12709300
H	-0.42054000	0.02346400	9.21236300
C	1.30088200	-2.15329800	5.51785400
H	1.81737400	-1.95801600	4.57848900
C	-1.14061200	-2.28384700	3.76922000
H	-1.54609900	-2.13907100	4.76969100
N	-1.24856100	-3.41173000	3.19161500
N	1.34364500	-3.30945000	6.04633500
O	2.15405500	-4.15635300	5.33470900
O	-1.96843100	-4.29633200	3.95602300
C	1.91494000	-5.50848900	5.68035600
H	2.79479700	-5.91281400	6.19669900
H	1.06398700	-5.55244900	6.36268000
C	1.67052600	-6.30164800	4.41378900
H	2.52283100	-6.16982200	3.74243100
H	1.60650000	-7.37102700	4.67030100
C	-1.80778100	-5.62298100	3.49073000
H	-1.63836100	-5.61493300	2.41087200
H	-2.75100600	-6.13122000	3.70873000
C	-0.68284000	-6.34125200	4.19460700
H	-0.78411700	-7.42594600	4.02365200
H	-0.77702900	-6.16153400	5.27448000

O	0.54249500	-5.87908500	3.69489700

1-TS-a

H (353 K, 1 atm) = -1030.641146 a.u.

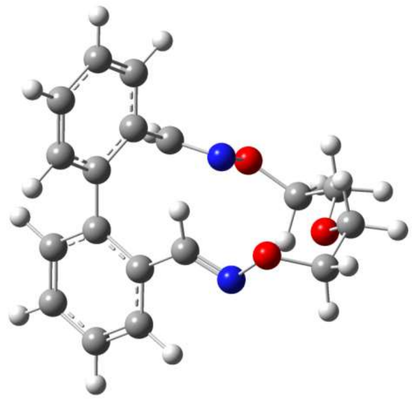

H	0.49005900	4.25937600	-3.69753800
C	0.56772700	4.05733800	-2.63529200
C	0.76240000	3.53208400	0.09587900
C	0.00186500	2.91143800	-2.10993100
C	1.22367300	4.95384800	-1.80118500
C	1.31532900	4.68896600	-0.44458900
C	0.08991900	2.63215500	-0.74484200
H	-0.52505200	2.21102700	-2.74654600
H	1.66186100	5.85863600	-2.20669200
H	1.83113300	5.38051500	0.21272600
C	0.89431600	3.26118300	1.55044900
C	1.05714000	2.72125000	4.28569200
C	1.84101100	2.34999300	2.03149600
C	0.05025600	3.89958800	2.45492400
C	0.12870700	3.63596000	3.81331600
C	1.91139900	2.08865500	3.39670400
H	-0.68666400	4.59950400	2.07660600
H	-0.54101000	4.13967100	4.50093800
H	2.64720200	1.37936500	3.75907400
H	1.11919400	2.50128700	5.34519100
C	2.82781100	1.71728300	1.12667600
H	3.77140400	2.23483600	0.93523600
C	-0.50149700	1.42371300	-0.17780700
H	-0.60991300	1.34819300	0.90265700
N	-0.84831900	0.45358500	-0.92077200
N	2.60872000	0.58221400	0.60920700

O	3.70698700	0.14241500	-0.09659900
O	-1.41409200	-0.56219300	-0.17751600
C	3.38000200	-0.87392500	-1.03433600
H	4.35120600	-1.30139100	-1.28965000
C	2.43389400	-1.94801100	-0.54036400
H	2.74746900	-2.92662000	-0.93695100
H	2.47240400	-2.00004900	0.55603300
C	-1.20433100	-1.80855200	-0.80972500
H	-1.26464100	-1.68415800	-1.89499800
H	-2.01443400	-2.45915200	-0.47032200
C	0.12520600	-2.41036200	-0.42100000
H	0.17106300	-3.45567400	-0.77167800
H	0.21023000	-2.41836200	0.67615300
O	1.14239100	-1.63872800	-0.98861400
H	2.94515000	-0.42481200	-1.93452300

1-b

H (353 K, 1 atm) = -1030.644045 a.u.

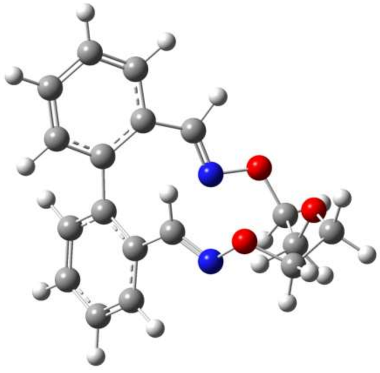

H	1.13886200	3.02899500	-4.45649800
C	0.80071900	2.78112900	-3.45721100
C	-0.07915700	2.10311800	-0.87858600
C	0.24222100	1.54314400	-3.20292300
C	0.91599900	3.69691400	-2.42450300
C	0.47480400	3.35118400	-1.15755400
C	-0.20542900	1.17273500	-1.92861200
H	0.13968800	0.82868200	-4.01289800
H	1.34775900	4.67553600	-2.59942300
H	0.56614000	4.06143400	-0.34323300
C	-0.52937500	1.88272600	0.51813900
C	-1.37808800	1.67371400	3.17076900
C	0.10873000	0.99175600	1.39030900

C	-1.58491000	2.65684000	0.99508700
C	-2.01740700	2.55135300	2.30575500
C	-0.32245500	0.90763600	2.71522000
H	-2.07640400	3.34273500	0.31400700
H	-2.84568300	3.15851000	2.65270100
H	0.19236600	0.22682300	3.38191100
H	-1.69831800	1.59345400	4.20332400
C	1.17693300	0.12236900	0.90282100
H	1.68190400	0.35013700	-0.03469900
C	-0.78453100	-0.16530400	-1.85600900
H	-0.85393200	-0.69793900	-2.80684800
N	-1.18976200	-0.76229300	-0.80668300
N	1.43516000	-0.94025800	1.54754900
O	2.41452700	-1.71137300	0.97133400
O	-1.68005400	-1.99956200	-1.14876000
C	2.26757000	-3.04517300	1.42946500
H	3.27078600	-3.44393900	1.61151100
H	1.72635700	-3.02340200	2.37832800
C	1.58236600	-3.94078200	0.40595500
H	2.24538800	-4.08312400	-0.45071600
H	1.43445000	-4.92501600	0.88356800
C	-1.89340700	-2.82645700	-0.02300100
H	-2.30536900	-3.73772600	-0.46111500
H	-2.63774900	-2.38298300	0.64929100
C	-0.63650900	-3.13533600	0.76417700
H	-0.85654400	-3.97666800	1.44224300
H	-0.35730600	-2.27200800	1.37497900
O	0.38677900	-3.45986800	-0.14169600

1-TS-rac

H (353 K, 1 atm) = -1030.609564 a.u.

H	-1.95052700	0.07128800	0.35907400
C	-1.42833100	0.04067000	1.30798400
C	-0.13209200	-0.10273300	3.84563400
C	-0.92065500	-1.14653900	1.78929800
C	-1.16579700	1.19434500	2.02457600
C	-0.53506900	1.10673400	3.24897600
C	-0.23038600	-1.24211000	3.00510900
H	-0.99470200	-2.04863000	1.19540700
H	-1.44624400	2.16726100	1.63769000
H	-0.33599900	2.03833000	3.75572700
C	0.26895400	-0.02091300	5.29666100
C	0.92321500	0.52985800	8.02905900
C	0.91459000	-0.97835700	6.11186500
C	-0.12147800	1.15290600	5.97267200
C	0.19776000	1.43986600	7.28397400
C	1.24313900	-0.67341200	7.43614000
H	-0.74776200	1.87107400	5.46797000
H	-0.14262700	2.37163000	7.72101400
H	1.74934700	-1.43737700	8.01456600
H	1.19308800	0.72753600	9.05955400
C	1.24819600	-2.38477300	5.78751400
H	2.18995900	-2.65607000	5.31060700
C	0.51065200	-2.50221700	3.14570200
H	1.56758100	-2.45137000	3.39399100
N	0.01293100	-3.60495000	2.75284300
N	0.47479000	-3.27728500	6.24947900
O	0.95103400	-4.54262200	6.02251800
O	0.96630600	-4.59073700	2.67574100

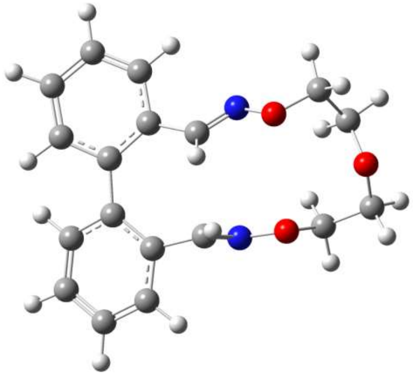

C	-0.03690000	-5.48947400	6.37700200
H	-0.13764600	-5.55948800	7.46774200
H	-1.00239200	-5.16841400	5.97682900
C	0.38891900	-6.83557000	5.83212300
H	1.29269600	-7.16913000	6.34724900
H	-0.41151100	-7.55890200	6.05575800
C	0.35845400	-5.83167400	2.36797500
H	1.17439800	-6.46386600	2.01171600
H	-0.37656600	-5.69777200	1.56772700
C	-0.29050300	-6.46415300	3.57232600
H	-0.87083600	-7.34623300	3.26053800
H	-0.98906800	-5.74002200	4.00495100
O	0.72465300	-6.83094900	4.47149500

1-c

H (353 K, 1 atm) = -1030.648032 a.u.

H	-3.19801900	1.46188700	-3.26386500
C	-2.50690700	1.58688700	-2.43807300
C	-0.70521700	1.91051300	-0.32294700
C	-1.36827100	0.80795600	-2.36721700
C	-2.75648200	2.53233600	-1.45253600
C	-1.85511800	2.69348800	-0.41450500
C	-0.46336000	0.94877400	-1.31414400
H	-1.15604600	0.06996000	-3.13127500
H	-3.64707600	3.14881500	-1.49574600
H	-2.04217000	3.43398400	0.35529200
C	0.24368600	2.21448300	0.78135700
C	1.99877000	3.09532400	2.79428100
C	0.55831500	1.36518100	1.86286600
C	0.81957300	3.48428900	0.75051900

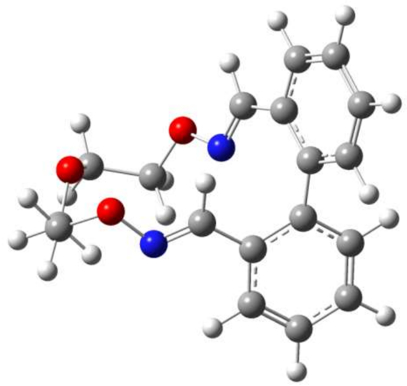

C	1.68911000	3.92913900	1.73275400
C	1.43251400	1.83636000	2.84985000
H	0.57526700	4.13310100	-0.08324700
H	2.11991700	4.92137500	1.66520800
H	1.66726300	1.18315900	3.68374500
H	2.67473600	3.42179200	3.57584100
C	0.06122700	0.01106900	2.08559500
H	0.35983900	-0.46676700	3.02063800
C	0.69246100	0.06630600	-1.20686600
H	1.49087700	0.31434600	-0.50886100
N	0.72136100	-1.02620200	-1.85461200
N	-0.64113400	-0.64460700	1.25216900
O	-0.91502600	-1.90186300	1.71145000
O	1.83722600	-1.76746500	-1.55618600
C	-1.53970800	-2.69736700	0.71464700
H	-2.56241200	-2.92320100	1.04031900
H	-1.58425400	-2.12625200	-0.21468900
C	-0.76661500	-3.98849900	0.54616100
H	-0.57262600	-4.41612000	1.53351300
H	-1.38815200	-4.70567300	-0.01132300
C	1.66293300	-3.09501200	-2.01624600
H	2.55851100	-3.61893300	-1.67734400
H	1.62514900	-3.11861900	-3.11270900
C	0.42091600	-3.75735800	-1.47187400
H	0.34623600	-4.77009400	-1.89925500
H	-0.45257000	-3.18671200	-1.80681100
O	0.48486800	-3.81620700	-0.06947700

1-d

H (353 K, 1 atm) = -1030.651589 a.u.

H	0.39592100	-0.07942300	-0.01108000
C	0.29710800	-0.08156600	-1.09053600
C	0.04101100	-0.09831900	-3.87318500
C	-0.31586500	-1.14557000	-1.72334700
C	0.77044800	0.98951100	-1.83807800
C	0.63835300	0.97611300	-3.21559100
C	-0.45634600	-1.17109100	-3.11249700
H	-0.71127900	-1.97630900	-1.15168300
H	1.24541700	1.83185100	-1.34831600
H	1.02367900	1.79927100	-3.80731900
C	-0.04860000	-0.07532600	-5.35192100
C	-0.31763100	-0.00187300	-8.13362300
C	0.52935300	-1.08101700	-6.14504000
C	-0.73278900	0.96506200	-5.97730800
C	-0.87037700	1.00595300	-7.35454700
C	0.38184900	-1.03126900	-7.53159500
H	-1.18290200	1.73655300	-5.36221000
H	-1.41287500	1.82127600	-7.81928600
H	0.84244200	-1.81023500	-8.12709300
H	-0.42054000	0.02346400	-9.21236300
C	1.30088200	-2.15329800	-5.51785400
H	1.81737400	-1.95801600	-4.57848900
C	-1.14061200	-2.28384700	-3.76922000
H	-1.54609900	-2.13907100	-4.76969100
N	-1.24856100	-3.41173000	-3.19161500
N	1.34364500	-3.30945000	-6.04633500
O	2.15405500	-4.15635300	-5.33470900

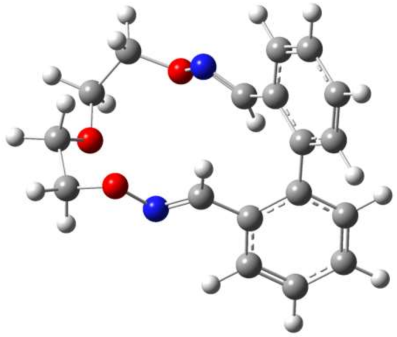

O	-1.96843100	-4.29633200	-3.95602300
C	1.91494000	-5.50848900	-5.68035600
H	2.79479700	-5.91281400	-6.19669900
H	1.06398700	-5.55244900	-6.36268000
C	1.67052600	-6.30164800	-4.41378900
H	2.52283100	-6.16982200	-3.74243100
H	1.60650000	-7.37102700	-4.67030100
C	-1.80778100	-5.62298100	-3.49073000
H	-1.63836100	-5.61493300	-2.41087200
H	-2.75100600	-6.13122000	-3.70873000
C	-0.68284000	-6.34125200	-4.19460700
H	-0.78411700	-7.42594600	-4.02365200
H	-0.77702900	-6.16153400	-5.27448000
O	0.54249500	-5.87908500	-3.69489700

2-a

H (353 K, 1 atm) = -1184.308997 a.u.

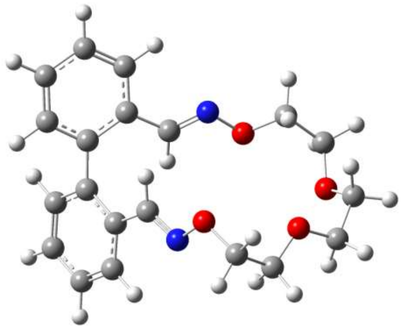

H	0.91949800	2.94469100	-3.51301900
C	0.92343100	3.03488700	-2.43280500
C	0.93224800	3.25333100	0.35495200
C	0.47035900	1.98982700	-1.65364200
C	1.37516600	4.20244000	-1.82942500
C	1.37306300	4.30417600	-0.45019700
C	0.46471800	2.07792300	-0.25838100
H	0.09997600	1.08099100	-2.11183000
H	1.73080000	5.02969600	-2.43285800
H	1.73543800	5.20717700	0.02861600
C	0.95260100	3.44175400	1.82627300
C	0.90419200	3.88633300	4.58675400
C	1.70233500	2.62234000	2.68639500
C	0.20403700	4.48383900	2.37260400

C	0.17341500	4.70778800	3.73778600
C	1.66457000	2.85944500	4.06248100
H	-0.37736400	5.11098400	1.70576500
H	-0.42134800	5.52025900	4.13922700
H	2.25682300	2.22391000	4.70944400
H	0.88906000	4.05557200	5.65730700
C	2.52700300	1.54416000	2.14493400
H	2.83449600	1.58069200	1.10004000
C	-0.04290500	0.97168100	0.55023100
H	-0.18216900	1.11534300	1.62038000
N	-0.31511000	-0.15297700	0.02085100
N	2.86341600	0.56104600	2.87931400
O	3.65355100	-0.32680800	2.20418600
O	-0.77027300	-1.04772000	0.95084400
C	3.85725100	-1.48754400	2.98860400
H	4.39756100	-1.22847500	3.90755900
H	2.88932600	-1.92153700	3.26075500
C	4.67037500	-2.45107200	2.15967300
H	5.53454500	-1.92445000	1.74531100
H	5.04791100	-3.25331200	2.80951100
C	-1.07712600	-2.28410900	0.33443700
H	-1.81320400	-2.13124000	-0.46336900
H	-1.53129400	-2.87613200	1.13136900
C	0.11680500	-3.00991900	-0.22995700
H	-0.25444400	-3.91918400	-0.73101200
H	0.61226300	-2.38610900	-0.98635600
O	1.00970700	-3.33376700	0.80086000
O	3.95217900	-2.97133300	1.07062100
C	3.17935800	-4.10867600	1.36509000
H	3.80081400	-5.01433300	1.29627400
H	2.75981700	-4.06292300	2.37874000
C	2.04073100	-4.19211600	0.38474200
H	2.41474500	-3.90590500	-0.60822400

H	1.67170800	-5.22777100	0.32263400

2-b

H (353 K, 1 atm) = -1184.307929 a.u.

H	-2.84976300	-1.41881100	-4.60467800
C	-2.10323900	-0.76991300	-4.16131300
C	-0.18395800	0.90545300	-3.00273400
C	-2.15623300	-0.48082600	-2.81275900
C	-1.09779800	-0.21930600	-4.94635800
C	-0.15496500	0.60945800	-4.36686100
C	-1.20895900	0.35414300	-2.21333600
H	-2.94344500	-0.89092700	-2.19221300
H	-1.04697300	-0.43871000	-6.00665200
H	0.64243200	1.02824900	-4.97072000
C	0.86443800	1.80404100	-2.46309500
C	2.80491100	3.58816400	-1.52255900
C	1.70451200	1.43968000	-1.39781000
C	1.02931500	3.06080100	-3.04582300
C	1.98572500	3.94816900	-2.58440500
C	2.66513300	2.34284700	-0.94006100
H	0.37355400	3.34611600	-3.86103700
H	2.08938600	4.92118700	-3.05079300
H	3.30718600	2.03879400	-0.12237800
H	3.55894500	4.27523300	-1.15618100
C	-1.30416800	0.65901700	-0.78481200
H	-0.74837400	1.50228900	-0.37880300
C	1.57015500	0.12740600	-0.76543600
H	1.21037100	-0.71955100	-1.34961100
N	-2.01339300	-0.07257400	-0.02219700
N	1.81833300	0.00201500	0.47527500
O	1.63030000	-1.28089600	0.91054200

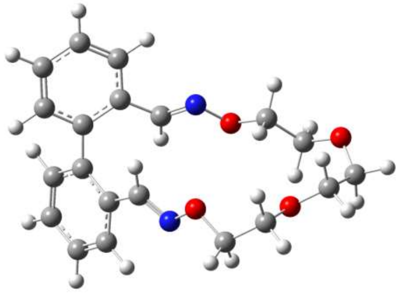

O	-1.94310800	0.32823100	1.28533300
C	1.58715400	-1.29256000	2.32735700
H	2.54524700	-0.95939000	2.73991600
H	0.79282200	-0.62841000	2.67751200
C	-2.89814200	-0.36791500	2.06780900
H	-3.08748200	-1.34696900	1.62181100
H	-3.83619500	0.20072400	2.08790500
C	1.29378500	-2.71478600	2.74793700
H	2.08275700	-3.38089200	2.38288000
H	0.34224300	-3.03594300	2.31357500
C	-2.37215200	-0.53051200	3.46676600
H	-3.21607600	-0.75411300	4.13811300
H	-1.91809800	0.41241300	3.80653700
O	1.27137900	-2.79099100	4.15861900
O	-1.43748200	-1.57691300	3.49726700
C	-0.89615200	-1.79702500	4.77546100
H	-1.70426300	-1.98582900	5.50130600
H	-0.33517300	-0.91483800	5.11922100
C	0.01670400	-3.00189300	4.75021300
H	0.22209100	-3.29215100	5.78447200
H	-0.51109600	-3.83456500	4.26369000

2-TS-rac

H (353 K, 1 atm) = -1184.266244 a.u.

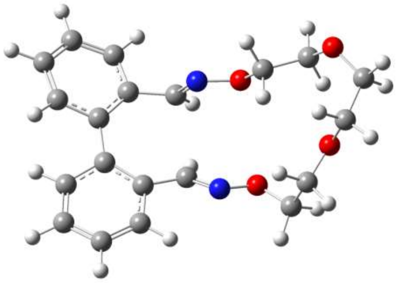

H	-4.08450600	0.06586200	3.42783800
C	-3.01896400	0.09549300	3.62253200
C	-0.23081400	0.20219800	4.22556700
C	-2.47109500	-0.66832200	4.62816900
C	-2.15846600	0.82196500	2.81867900
C	-0.81343100	0.85565400	3.12124700

C	-1.09976900	-0.67816900	4.92246500
H	-3.09924600	-1.33750600	5.20157700
H	-2.52348000	1.35621500	1.94903000
H	-0.17972000	1.40534800	2.44268400
C	1.20933300	0.56234000	4.48796000
C	3.95246200	1.36925300	4.44134400
C	2.19195100	-0.11151200	5.24621500
C	1.66193200	1.74997000	3.87561200
C	2.98153700	2.15008800	3.84010800
C	3.53409500	0.26865700	5.15875300
H	0.94267300	2.41696300	3.42590900
H	3.24278100	3.07754300	3.34364200
H	4.25822600	-0.30611300	5.72664900
H	5.00038900	1.64208900	4.40710300
C	-0.70865100	-1.76815300	5.82536600
H	0.20017800	-2.32612600	5.62551600
C	2.00224100	-1.13909500	6.29310900
H	2.35259900	-2.16267900	6.13918600
N	-1.51313600	-2.17905500	6.72012400
N	1.60424400	-0.75478000	7.43355200
O	1.64484900	-1.76329200	8.35770900
O	-1.06932000	-3.33312000	7.32434300
C	1.04903400	-1.33244400	9.56921600
H	1.40639400	-0.33108900	9.82436800
H	-0.03828600	-1.30530300	9.45955600
C	-2.11380600	-3.88451000	8.10633400
H	-1.77577900	-4.89392300	8.34970900
H	-3.02819400	-3.94861100	7.50735300
C	1.44934800	-2.32688500	10.63748700
H	2.53590400	-2.30269400	10.77028000
H	1.16026100	-3.33554400	10.32876200
C	-2.39498800	-3.12589000	9.37731700
H	-3.33970000	-3.49452900	9.80864400

H	-2.52397300	-2.05845600	9.14813100
O	0.85297400	-1.96933000	11.86745300
O	-1.33391700	-3.32489200	10.27269400
C	-1.48283500	-2.63656100	11.48802600
H	-2.32697200	-3.05260800	12.06196900
H	-1.69010500	-1.57042800	11.31148100
C	-0.21391700	-2.77452100	12.29739600
H	-0.42217100	-2.46446300	13.32487100
H	0.07864800	-3.83442500	12.31484700

2-c

H (353 K, 1 atm) = -1184.308306 a.u.

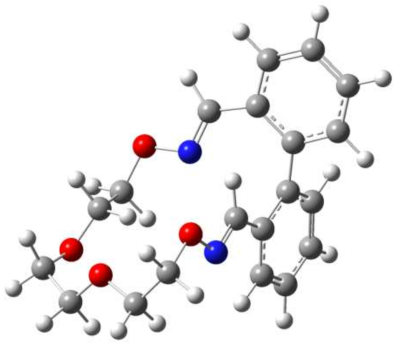

H	-3.93802200	2.16293100	-1.05706000
C	-2.90177400	1.96456800	-1.30611500
C	-0.23103600	1.44637500	-1.93053900
C	-2.34176000	0.73969700	-0.99932300
C	-2.13283800	2.93987200	-1.92728000
C	-0.81079900	2.67318600	-2.24138100
C	-1.00755800	0.46407700	-1.30222000
H	-2.92794300	-0.02662900	-0.50703900
H	-2.56178000	3.90587300	-2.16795800
H	-0.20536300	3.42763700	-2.73143200
C	1.17127500	1.20654900	-2.36606000
C	3.74806800	0.85877300	-3.42302800
C	2.29219700	1.24761500	-1.51515600
C	1.37668800	0.98945500	-3.72606700
C	2.64396000	0.81049700	-4.25838300
C	3.56489100	1.08189600	-2.07126100
H	0.50945200	0.96232300	-4.37647700
H	2.76478400	0.63992100	-5.32189500

H	4.42734500	1.12809500	-1.41434200
H	4.74744000	0.72754000	-3.82106100
C	-0.40305900	-0.81073400	-0.93338800
H	0.67330700	-0.93341200	-1.04463700
C	2.25669300	1.46879000	-0.07285500
H	3.20615900	1.70278800	0.41365400
N	-1.10519200	-1.75720800	-0.45596500
N	1.20282500	1.37081500	0.63255200
O	1.45587700	1.63797800	1.95124100
O	-0.32592000	-2.83044300	-0.10449500
C	0.30596300	1.37336100	2.73627200
H	0.44703300	1.93661600	3.66086600
H	-0.58759400	1.74082100	2.22503600
C	-1.12209900	-3.86905300	0.43296000
H	-0.39727100	-4.62412400	0.74302700
H	-1.77070100	-4.29506000	-0.34287400
C	0.18087900	-0.10227500	3.05161500
H	1.13166800	-0.46597300	3.46913800
H	-0.03072900	-0.67601700	2.14356800
C	-1.97647600	-3.44714500	1.60176900
H	-2.51268900	-4.33664400	1.97178000
H	-2.72534100	-2.71704100	1.26912000
O	-0.85426100	-0.22020000	4.00058000
O	-1.16668600	-2.89058900	2.60064200
C	-1.90227900	-2.40543900	3.69447700
H	-2.27220900	-3.24229800	4.30967400
H	-2.77462400	-1.83139200	3.34895200
C	-1.03775600	-1.50345000	4.54208000
H	-1.53531100	-1.35917600	5.50586600
H	-0.07090100	-1.99255900	4.72803100

2-TS-b

H (353 K, 1 atm) = -1184.302503 a.u.

H	-4.06016500	2.47768500	-2.16992900
C	-3.00697500	2.22486200	-2.21485800
C	-0.29646200	1.56575000	-2.32079100
C	-2.56772600	1.01541200	-1.71473800
C	-2.09904500	3.11170600	-2.78016100
C	-0.75698900	2.77681500	-2.83273100
C	-1.21516700	0.66718300	-1.75843400
H	-3.26719900	0.31027400	-1.28240000
H	-2.43610400	4.06161200	-3.17901400
H	-0.04106100	3.46448500	-3.26950300
C	1.15728100	1.26770100	-2.41218200
C	3.89638200	0.78060900	-2.68267900
C	2.00467400	1.37715500	-1.30331500
C	1.70317300	0.91399600	-3.64421400
C	3.05944600	0.66775400	-3.78238000
C	3.36820500	1.14039500	-1.45387800
H	1.04359400	0.82722300	-4.50062000
H	3.46181500	0.38776500	-4.74921300
H	4.01674500	1.23338500	-0.58960000
H	4.95862500	0.58873000	-2.78031200
C	-0.75233600	-0.61615900	-1.24130100
H	0.29889100	-0.87590000	-1.34523500
C	1.48761600	1.79576400	0.01822000
H	1.60503800	2.83425200	0.33762700
N	-1.55078100	-1.42159300	-0.66595800
N	0.91806500	0.95175900	0.77127900
O	0.48661100	1.51806900	1.94346600

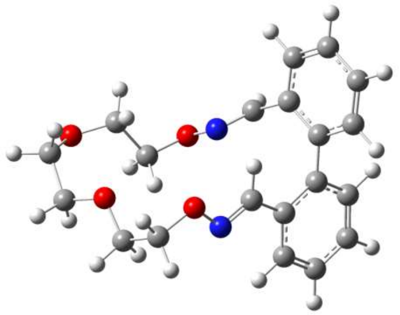

O	-0.89951200	-2.56554600	-0.27372300
C	-0.12389800	0.54109500	2.76995200
H	-0.64459700	1.11484900	3.53898500
H	-0.85006600	-0.03722800	2.19222200
C	-1.77876600	-3.40343700	0.44582400
H	-1.23221400	-4.34220900	0.55655200
H	-2.69041600	-3.58277000	-0.13568000
C	0.89759600	-0.35944000	3.44873800
H	1.77375700	0.24978300	3.69663900
H	1.21023500	-1.16552100	2.77844200
C	-2.15211500	-2.86696300	1.80425200
H	-2.85169300	-3.57963400	2.26974200
H	-2.67321400	-1.90460500	1.70071600
O	0.37358900	-0.86477100	4.65958500
O	-0.99591300	-2.72196600	2.58642100
C	-1.27277300	-2.54749300	3.95294500
H	-1.70794800	-3.47148900	4.36741100
H	-1.99957800	-1.73632200	4.11034300
C	0.00439200	-2.21825000	4.69230200
H	-0.13567900	-2.45453200	5.75057800
H	0.80919500	-2.85905400	4.30521400

2-d

H (353 K, 1 atm) = -1184.304957 a.u.

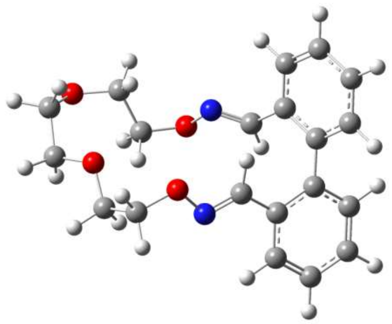

H	-4.11707200	2.52753700	-2.71544600
C	-3.06284000	2.28018700	-2.66653200
C	-0.34790800	1.64005700	-2.53025600
C	-2.65716500	1.11902400	-2.03870600
C	-2.12066200	3.12066800	-3.24727100
C	-0.77696500	2.79618500	-3.17892700
C	-1.30451900	0.77834200	-1.96251700

H	-3.38412300	0.44214500	-1.60635700
H	-2.43321700	4.02934200	-3.74881100
H	-0.03398600	3.45520700	-3.61460800
C	1.10210000	1.34511900	-2.44967100
C	3.80292700	0.64754800	-2.31438500
C	1.74827500	1.25509200	-1.20697600
C	1.83467700	1.11339800	-3.60972800
C	3.17601500	0.76924500	-3.54658500
C	3.09280300	0.89527700	-1.15219500
H	1.33211400	1.17712400	-4.56867400
H	3.72941500	0.58636400	-4.46055600
H	3.57305400	0.83123400	-0.18312300
H	4.84978400	0.37209600	-2.25995900
C	-0.88195400	-0.48075800	-1.35407200
H	0.14408400	-0.81404100	-1.50045900
C	1.00530200	1.57427900	0.01325800
H	0.30304900	2.40911700	0.00129000
N	-1.68284400	-1.19936400	-0.67439600
N	1.15397100	0.88095800	1.06708200
O	0.43553200	1.42015400	2.11414500
O	-1.06616900	-2.35349800	-0.25918900
C	0.03194400	0.40576000	3.01643200
H	-0.41426100	0.94672400	3.85292200
H	-0.73244400	-0.22028700	2.54669500
C	-1.94619900	-3.14289100	0.51405300
H	-1.48413800	-4.13231200	0.52992500
H	-2.92172500	-3.20784900	0.02057500
C	1.17121200	-0.45183400	3.54395600
H	2.01680900	0.19799000	3.78967400
H	1.49729200	-1.17584900	2.79267600
C	-2.12912600	-2.65036700	1.92706400
H	-2.86063200	-3.30938900	2.42106300
H	-2.54672200	-1.63267800	1.92372400

O	0.77107700	-1.09396400	4.73774600
O	-0.89762500	-2.68118800	2.60027800
C	-1.02651400	-2.61416300	3.99983000
H	-1.47847500	-3.54641000	4.37475700
H	-1.68152700	-1.78275700	4.29983800
C	0.33595100	-2.42480200	4.63352500
H	0.29405700	-2.79392900	5.66166500
H	1.06389800	-3.03665400	4.08183200

2-e

H (353 K, 1 atm) = -1184.308997 a.u.

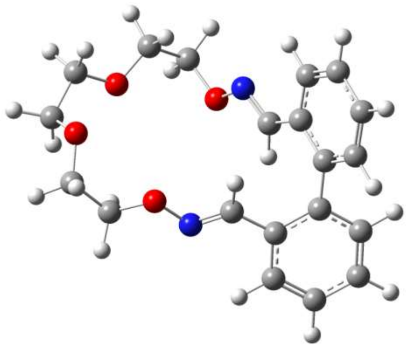

H	0.91949800	2.94469100	3.51301900
C	0.92343100	3.03488700	2.43280500
C	0.93224800	3.25333100	-0.35495200
C	0.47035900	1.98982700	1.65364200
C	1.37516600	4.20244000	1.82942500
C	1.37306300	4.30417600	0.45019700
C	0.46471800	2.07792300	0.25838100
H	0.09997600	1.08099100	2.11183000
H	1.73080000	5.02969600	2.43285800
H	1.73543800	5.20717700	-0.02861600
C	0.95260100	3.44175400	-1.82627300
C	0.90419200	3.88633300	-4.58675400
C	1.70233500	2.62234000	-2.68639500
C	0.20403700	4.48383900	-2.37260400
C	0.17341500	4.70778800	-3.73778600
C	1.66457000	2.85944500	-4.06248100
H	-0.37736400	5.11098400	-1.70576500
H	-0.42134800	5.52025900	-4.13922700
H	2.25682300	2.22391000	-4.70944400
H	0.88906000	4.05557200	-5.65730700

C	2.52700300	1.54416000	-2.14493400
H	2.83449600	1.58069200	-1.10004000
C	-0.04290500	0.97168100	-0.55023100
H	-0.18216900	1.11534300	-1.62038000
N	-0.31511000	-0.15297700	-0.02085100
N	2.86341600	0.56104600	-2.87931400
O	3.65355100	-0.32680800	-2.20418600
O	-0.77027300	-1.04772000	-0.95084400
C	3.85725100	-1.48754400	-2.98860400
H	4.39756100	-1.22847500	-3.90755900
H	2.88932600	-1.92153700	-3.26075500
C	4.67037500	-2.45107200	-2.15967300
H	5.53454500	-1.92445000	-1.74531100
H	5.04791100	-3.25331200	-2.80951100
C	-1.07712600	-2.28410900	-0.33443700
H	-1.81320400	-2.13124000	0.46336900
H	-1.53129400	-2.87613200	-1.13136900
C	0.11680500	-3.00991900	0.22995700
H	-0.25444400	-3.91918400	0.73101200
H	0.61226300	-2.38610900	0.98635600
O	1.00970700	-3.33376700	-0.80086000
O	3.95217900	-2.97133300	-1.07062100
C	3.17935800	-4.10867600	-1.36509000
H	3.80081400	-5.01433300	-1.29627400
H	2.75981700	-4.06292300	-2.37874000
C	2.04073100	-4.19211600	-0.38474200
H	2.41474500	-3.90590500	0.60822400
H	1.67170800	-5.22777100	-0.32263400

3-a

H (353 K, 1 atm) = -1337.96462 a.u.

H	-4.60260300	3.38110700	2.79114700
C	-3.56718600	3.06078900	2.80799900
C	-0.89931700	2.22578200	2.84416600
C	-3.20946000	1.85815200	2.23217900
C	-2.60017500	3.85056700	3.41800200
C	-1.28243200	3.42968600	3.43486500
C	-1.88164300	1.42246700	2.24097200
H	-3.95581400	1.22325000	1.77055300
H	-2.87271700	4.79405100	3.87685700
H	-0.51984300	4.04752400	3.89605200
C	0.53077400	1.83564800	2.89551700
C	3.21721200	1.09724900	3.11941500
C	1.30950800	1.65265000	1.74020700
C	1.12541800	1.66121200	4.14517500
C	2.45337900	1.29271400	4.26375100
C	2.65055100	1.28174000	1.87432300
H	0.51855800	1.80176600	5.03287700
H	2.89145700	1.15519100	5.24566600
H	3.23980700	1.15196500	0.97484100
H	4.25912000	0.80956100	3.20106100
C	-1.51923900	0.13426000	1.65490100
H	-0.54547400	-0.29729300	1.88334800
C	0.73217300	1.87725800	0.41650700
H	-0.23761200	2.36635900	0.33496400
N	-2.32237100	-0.47769300	0.88049400
N	1.35288300	1.51063900	-0.63187700
O	-1.79958900	-1.66660000	0.45141900
O	0.66633300	1.83691900	-1.77027000

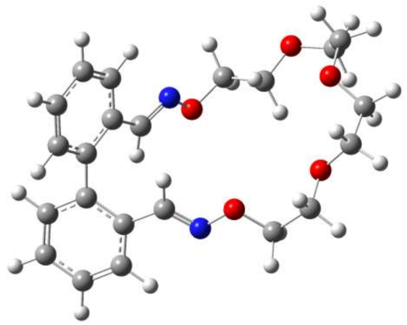

C	1.38874400	1.41029700	-2.91007000
H	2.44681900	1.66456900	-2.80091800
H	0.97519900	1.97847700	-3.74538200
C	1.24322200	-0.07538600	-3.16325800
H	0.19478900	-0.33044400	-3.37275300
H	1.54935500	-0.64451300	-2.27881000
O	2.06348700	-0.34979300	-4.27739200
C	0.47560200	-4.01129000	-3.47234700
H	0.87925200	-4.93614500	-3.91546000
H	-0.14554000	-3.51945300	-4.23393600
C	-0.39550100	-4.37649400	-2.30403700
H	0.23169500	-4.73765100	-1.47553900
H	-1.05922600	-5.20143600	-2.61038500
O	-1.14915600	-3.26382500	-1.90485700
C	-2.07731900	-3.57849500	-0.90213900
H	-1.58810000	-4.09684700	-0.06391700
H	-2.85787200	-4.24779600	-1.29783000
C	-2.72511000	-2.32212000	-0.39562100
H	-2.99630400	-1.67072000	-1.23229600
H	-3.63146900	-2.57096700	0.16846500
C	1.86337500	-1.59271900	-4.89405900
H	2.41775100	-1.54660900	-5.83630300
H	0.79990900	-1.73297700	-5.14064500
C	2.37545100	-2.77769400	-4.09421900
H	2.56240900	-3.62044800	-4.77846900
H	3.32822100	-2.50018100	-3.63499700
O	1.52521100	-3.18387100	-3.04681500

3-b

H (353 K, 1 atm) = -1337.962062 a.u.

H	-2.78743700	-1.51489500	-4.27844500
C	-2.14918800	-0.71197400	-3.92780700
C	-0.50129900	1.35419000	-3.00325700
C	-2.17770500	-0.34041400	-2.59985400
C	-1.30002400	-0.05470100	-4.80973600
C	-0.49201200	0.96578000	-4.34496400
C	-1.36607900	0.69048600	-2.11455500
H	-2.83786700	-0.84204000	-1.90329900
H	-1.26439500	-0.34059000	-5.85471100
H	0.18169100	1.47516300	-5.02498600
C	0.38157000	2.48237300	-2.61398000
C	1.99415800	4.68839700	-2.00655500
C	1.38102000	2.37878600	-1.63276100
C	0.22068900	3.69836600	-3.27956000
C	1.01204100	4.79367900	-2.98268100
C	2.17581700	3.49026200	-1.34374700
H	-0.55767500	3.77987500	-4.03020500
H	0.86187300	5.72805600	-3.51128000
H	2.94705200	3.38663600	-0.59033900
H	2.62374300	5.53843000	-1.76986700
C	-1.43104700	1.06274800	-0.70111800
H	-0.83005800	1.89250500	-0.33549200
C	1.58035700	1.12702300	-0.90381600
H	1.29700500	0.18018700	-1.36336200
N	-2.16411000	0.40546900	0.10525800
N	2.01668600	1.16531600	0.29076200
O	-2.02158600	0.84498000	1.39458200
O	2.10002300	-0.07414600	0.85176700

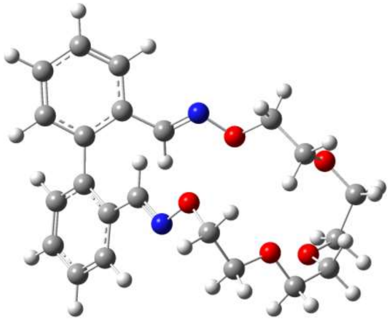

C	2.39264300	0.04472900	2.23669200
H	3.47014200	0.18467800	2.38087900
H	1.86090200	0.91111600	2.63854200
C	1.91996100	-1.19607600	2.93366000
H	1.91918500	-1.00530000	4.01894400
H	0.89439200	-1.41623500	2.61896100
O	2.79528000	-2.26136700	2.63620200
C	-0.66073900	-3.75351200	3.24963000
H	-0.79824700	-4.80016300	3.56470900
H	-0.26338500	-3.19424600	4.10832900
C	-1.99627500	-3.17829900	2.86791000
H	-2.29725500	-3.59988100	1.89792800
H	-2.74800500	-3.47857300	3.61550400
O	-1.91796200	-1.77789600	2.79429300
C	-3.08113800	-1.22574600	2.23708300
H	-3.22994500	-1.59586000	1.21481200
H	-3.96634800	-1.51069200	2.83034900
C	-3.01745200	0.27947400	2.22887100
H	-2.77421500	0.65228100	3.22567400
H	-4.00370400	0.66336500	1.93919000
C	2.41627100	-3.48850900	3.19939100
H	3.32877500	-4.08502800	3.30621300
H	2.00773000	-3.34139700	4.21054800
C	1.45546200	-4.27896400	2.33053200
H	1.34652200	-5.29130700	2.75520400
H	1.89089600	-4.37206600	1.33286500
O	0.19410800	-3.68673000	2.14025400

3-TS-rac

H (353 K, 1 atm) = -1337.920734 a.u.

H	-3.84842000	-1.15461600	2.91565500
C	-2.85294000	-0.85939400	3.22519000
C	-0.21296500	-0.12147100	4.08258600
C	-2.27824000	-1.40158500	4.35312200
C	-2.12070200	0.07451600	2.52023600
C	-0.85932800	0.42388100	2.95567300
C	-0.99287100	-1.06523600	4.80380100
H	-2.82734800	-2.13217000	4.93214900
H	-2.52625300	0.55230800	1.63571900
H	-0.38197000	1.20295800	2.38732200
C	1.14106600	0.46900000	4.41675800
C	3.41267500	2.11586800	4.99120900
C	1.91479800	0.28679800	5.59012600
C	1.68070300	1.39916500	3.50741500
C	2.77524900	2.19884500	3.76734000
C	2.99107800	1.13911500	5.86652700
H	1.24283800	1.51960500	2.53067200
H	3.11786300	2.89056300	3.00630600
H	3.52451900	0.97257200	6.79381700
H	4.25237800	2.75459900	5.23789700
C	-0.65536600	-1.82490900	6.02651200
H	0.15555500	-2.54586000	6.04090400
C	1.84201700	-0.84442900	6.52326400
H	1.93621100	-1.85126600	6.12144400
N	-1.47503700	-1.77919100	6.99713600
N	1.88859000	-0.65245100	7.77788900
O	-1.17410300	-2.69929500	7.97102500
O	2.01590500	-1.82615000	8.46641400

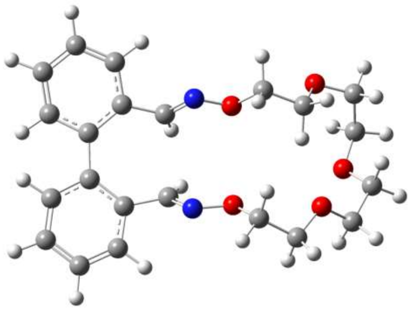

C	1.97451400	-1.56574800	9.85952200
H	2.87916700	-1.02905900	10.16683400
H	1.10063700	-0.94542000	10.08339200
C	1.85261700	-2.87059800	10.58680300
H	1.56509200	-2.65349700	11.62813500
H	1.05809800	-3.46700500	10.13186600
O	3.08988300	-3.55172100	10.56772300
C	0.28882000	-6.02469500	11.18724100
H	0.30593700	-7.04928800	11.59107200
H	0.48069200	-5.33277500	12.01822000
C	-1.07455800	-5.73092000	10.62161400
H	-1.22696300	-6.33243900	9.71394900
H	-1.84469700	-6.01684800	11.35530300
O	-1.14957800	-4.36295400	10.32125200
C	-2.30207300	-3.99258800	9.61397900
H	-2.50068300	-4.70334200	8.79897000
H	-3.18075700	-3.98417700	10.27851300
C	-2.11395800	-2.61985300	9.02858700
H	-1.75586000	-1.92422700	9.79686500
H	-3.06519000	-2.25036700	8.63694400
C	3.09445000	-4.73572500	11.31837500
H	4.13756900	-4.93976800	11.58349300
H	2.54640100	-4.59284400	12.26238700
C	2.57793900	-5.95084500	10.56649400
H	2.75251100	-6.84634400	11.18650400
H	3.15458200	-6.05855700	9.64483200
O	1.23262400	-5.88045200	10.16067400

3-c

H (353 K, 1 atm) = -1337.96462 a.u.

H	-4.60260300	3.38110700	-2.79114700
C	-3.56718600	3.06078900	-2.80799900
C	-0.89931700	2.22578200	-2.84416600
C	-3.20946000	1.85815200	-2.23217900
C	-2.60017500	3.85056700	-3.41800200
C	-1.28243200	3.42968600	-3.43486500
C	-1.88164300	1.42246700	-2.24097200
H	-3.95581400	1.22325000	-1.77055300
H	-2.87271700	4.79405100	-3.87685700
H	-0.51984300	4.04752400	-3.89605200
C	0.53077400	1.83564800	-2.89551700
C	3.21721200	1.09724900	-3.11941500
C	1.30950800	1.65265000	-1.74020700
C	1.12541800	1.66121200	-4.14517500
C	2.45337900	1.29271400	-4.26375100
C	2.65055100	1.28174000	-1.87432300
H	0.51855800	1.80176600	-5.03287700
H	2.89145700	1.15519100	-5.24566600
H	3.23980700	1.15196500	-0.97484100
H	4.25912000	0.80956100	-3.20106100
C	-1.51923900	0.13426000	-1.65490100
H	-0.54547400	-0.29729300	-1.88334800
C	0.73217300	1.87725800	-0.41650700
H	-0.23761200	2.36635900	-0.33496400
N	-2.32237100	-0.47769300	-0.88049400
N	1.35288300	1.51063900	0.63187700
O	-1.79958900	-1.66660000	-0.45141900
O	0.66633300	1.83691900	1.77027000

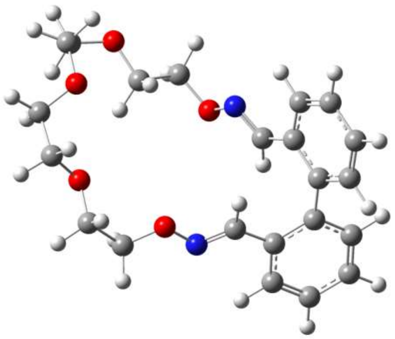

C	1.38874400	1.41029700	2.91007000
H	2.44681900	1.66456900	2.80091800
H	0.97519900	1.97847700	3.74538200
C	1.24322200	-0.07538600	3.16325800
H	0.19478900	-0.33044400	3.37275300
H	1.54935500	-0.64451300	2.27881000
O	2.06348700	-0.34979300	4.27739200
C	0.47560200	-4.01129000	3.47234700
H	0.87925200	-4.93614500	3.91546000
H	-0.14554000	-3.51945300	4.23393600
C	-0.39550100	-4.37649400	2.30403700
H	0.23169500	-4.73765100	1.47553900
H	-1.05922600	-5.20143600	2.61038500
O	-1.14915600	-3.26382500	1.90485700
C	-2.07731900	-3.57849500	0.90213900
H	-1.58810000	-4.09684700	0.06391700
H	-2.85787200	-4.24779600	1.29783000
C	-2.72511000	-2.32212000	0.39562100
H	-2.99630400	-1.67072000	1.23229600
H	-3.63146900	-2.57096700	-0.16846500
C	1.86337500	-1.59271900	4.89405900
H	2.41775100	-1.54660900	5.83630300
H	0.79990900	-1.73297700	5.14064500
C	2.37545100	-2.77769400	4.09421900
H	2.56240900	-3.62044800	4.77846900
H	3.32822100	-2.50018100	3.63499700
O	1.52521100	-3.18387100	3.04681500

4-a

H (393 K, 1 atm) = -1107.763925 a.u.

C	2.83222700	-0.41984700	0.09757300
C	3.34774100	-2.94309600	1.18041700
C	1.95339200	-1.48681000	-0.15207600
C	3.96986700	-0.64711000	0.86827200
C	4.22960500	-1.89515000	1.40941500
C	2.22567400	-2.74032000	0.39935800
H	4.64363000	0.18087500	1.05946500
H	5.11772300	-2.04894700	2.01153200
H	1.54446900	-3.55540400	0.18730500
H	3.54457800	-3.92332900	1.59902300
C	2.57369900	0.94443900	-0.42387400
C	2.13352300	3.49317700	-1.47921900
C	3.44412000	1.48673300	-1.36705400
C	1.47670400	1.70755400	0.01013900
C	1.27170700	2.97904100	-0.53023100
C	3.22947100	2.74786500	-1.89595400
H	4.28897800	0.89300600	-1.69832400
H	0.42614000	3.55779100	-0.17898200
H	3.91486700	3.14841000	-2.63403700
H	1.95931500	4.48189600	-1.88776900
C	0.57903300	1.18055400	1.03607800
H	0.91085400	0.34066100	1.64610600
C	0.78436600	-1.29287200	-1.00930700
H	0.79840900	-0.50270200	-1.75961300
N	-0.57873000	1.67744500	1.20808700
N	-0.24116500	-2.03475900	-0.87304600
O	-1.19425000	-1.77036000	-1.82477100
O	-1.23914100	1.06267400	2.25034600

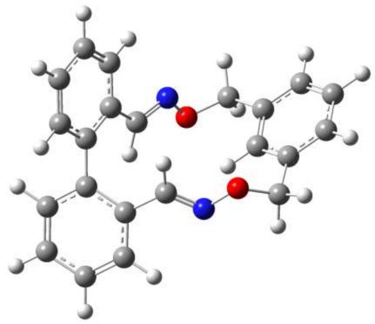

C	-2.47618700	-2.21126400	-1.38871500
H	-3.00432400	-2.52800000	-2.29058700
H	-2.33845900	-3.07701900	-0.73437500
C	-2.62438100	1.32630100	2.15028000
H	-2.79110100	2.40593300	2.06905200
H	-3.03514200	0.98506600	3.10484700
C	-3.22875800	-1.10796700	-0.69965700
C	-4.51232800	1.04172400	0.51380600
C	-2.66299600	-0.47498200	0.40219300
C	-4.45734000	-0.66915200	-1.17389500
C	-5.10493700	0.39303000	-0.55666800
C	-3.28090000	0.61202700	0.99892100
H	-4.90372800	-1.14728200	-2.03999000
H	-5.00277800	1.89818200	0.96647300
H	-6.06256700	0.73481800	-0.93301500
H	-1.70605800	-0.81373200	0.77722700

4-TS-a

H (393 K, 1 atm) = -1107.749651 a.u.

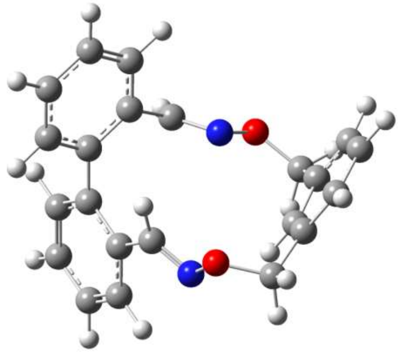

H	0.42219600	4.41324400	-3.60338600
C	0.50177500	4.15844000	-2.55278500
C	0.69215300	3.50151100	0.15201400
C	-0.01976000	2.96433500	-2.09261200
C	1.11485600	5.03447600	-1.66662600
C	1.20471600	4.70388700	-0.32421700
C	0.06993000	2.61721300	-0.74289200
H	-0.51312700	2.27823300	-2.77041400
H	1.51868900	5.97639000	-2.01986200
H	1.68369300	5.38225700	0.37335200
C	0.82136200	3.16008300	1.59246500

C	0.97129400	2.43636200	4.28350700
C	1.89499100	2.39100300	2.05250100
C	-0.15651600	3.56870000	2.49466500
C	-0.08331900	3.21520200	3.83294900
C	1.95480000	2.03094000	3.39565200
H	-0.99124700	4.15785600	2.13086700
H	-0.85576900	3.54030200	4.52045300
H	2.78409900	1.42281200	3.73854400
H	1.02926800	2.14346000	5.32541700
C	2.99477900	1.98246800	1.14820800
H	3.79209000	2.69244300	0.91502100
C	-0.45523200	1.34126200	-0.25876800
H	-0.61556900	1.20113200	0.80900300
N	-0.67774700	0.38827200	-1.07104000
N	3.02084500	0.80100600	0.69296900
O	4.18078900	0.54214300	-0.00006400
O	-1.20141600	-0.71763500	-0.43552700
C	4.02332000	-0.51562100	-0.95774100
H	5.01967700	-0.95605700	-1.03541100
C	-0.73681300	-1.89953000	-1.08661300
H	-0.81486800	-1.75733000	-2.16900200
H	-1.42705300	-2.68479000	-0.77408900
C	0.67036400	-2.21647100	-0.66814100
C	3.26102600	-2.56465200	0.30368800
C	0.95317100	-3.23661100	0.23186300
C	1.70832500	-1.39742400	-1.09460800
C	2.99170000	-1.53446800	-0.58999900
C	2.24758300	-3.42373200	0.69847800
H	0.15402400	-3.88432900	0.57840800
H	1.49546500	-0.59312900	-1.78987900
H	2.46037400	-4.22983700	1.39155600
H	4.26295800	-2.68338800	0.70316000
H	3.76585400	-0.06328900	-1.92206400

4-b

H (393 K, 1 atm) = -1107.756577 a.u.

H	0.88973400	4.24983400	-3.56259400
C	0.90094100	3.98970000	-2.51032000
C	0.90687800	3.32416400	0.19965800
C	0.18415100	2.89715800	-2.06300000
C	1.63026100	4.75408300	-1.60864700
C	1.62413100	4.42257800	-0.26461100
C	0.18391700	2.54480300	-0.71143400
H	-0.38641600	2.28964400	-2.75486500
H	2.19545900	5.61356000	-1.95069200
H	2.18388300	5.02012500	0.44625100
C	0.88562900	3.06413800	1.66394000
C	0.78395400	2.83877200	4.45633300
C	1.84565500	2.29145700	2.33997000
C	-0.11118900	3.69168000	2.40589200
C	-0.17549900	3.58033500	3.78606900
C	1.78234400	2.21136900	3.73484700
H	-0.84610200	4.28869900	1.87718600
H	-0.96580200	4.08124200	4.33308300
H	2.53361200	1.62699900	4.25594800
H	0.75678200	2.74951400	5.53599800
C	2.92390100	1.52830700	1.71404200
H	3.74968900	1.22604100	2.36191200
C	-0.47481300	1.32279000	-0.25753800
H	-0.59070300	1.13966100	0.80997400
N	-0.81106000	0.43995300	-1.10773300
N	2.91517700	1.14375600	0.50241100
O	4.05203900	0.42359500	0.21173400

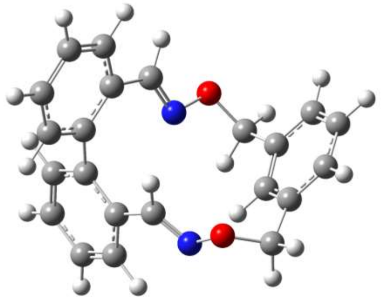

O	-1.35032000	-0.68649700	-0.51940700
C	3.85682700	-0.33935800	-0.97894800
H	4.84912800	-0.73876300	-1.19735300
C	-0.88787500	-1.82800300	-1.24121000
H	-1.00114200	-1.63340600	-2.31256500
H	-1.55542900	-2.64010600	-0.94888400
C	0.53742300	-2.14781700	-0.88310000
C	3.15481700	-2.55494500	-0.00764500
C	0.85913300	-3.26052000	-0.11709200
C	1.55321900	-1.25974200	-1.22895200
C	2.84827200	-1.42931500	-0.76757200
C	2.16715900	-3.47595000	0.29941000
H	0.07964700	-3.96071200	0.16655000
H	1.31674400	-0.38579300	-1.82323300
H	2.40879500	-4.35435400	0.88724400
H	4.16657300	-2.69836800	0.35792300
H	3.54821100	0.32683100	-1.78998100

4-TS-b

H (393 K, 1 atm) = -1107.753452 a.u.

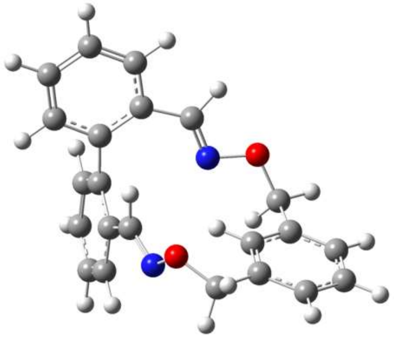

H	0.84592100	3.83906700	-3.66663500
C	0.86625100	3.71282100	-2.59024700
C	0.90032200	3.38860300	0.18304000
C	0.16291600	2.67875400	-2.00481900
C	1.59792300	4.58731000	-1.79693300
C	1.60480500	4.42563700	-0.42211900
C	0.17459000	2.49849300	-0.62018200
H	-0.40276800	1.98212900	-2.61136400
H	2.15581200	5.39990000	-2.24809300
H	2.16490500	5.11129200	0.20382600
C	0.86073800	3.33841200	1.66964800

C	0.64158700	3.50651400	4.46370700
C	1.64169000	2.49593100	2.48449200
C	-0.00478900	4.24193400	2.28208400
C	-0.12499900	4.33302300	3.65961800
C	1.51069600	2.60834600	3.87468600
H	-0.60000100	4.88764200	1.64596900
H	-0.81286100	5.04713500	4.09723700
H	2.11545200	1.96334200	4.50337600
H	0.56669200	3.56045800	5.54340400
C	2.60385200	1.48545500	2.05165400
H	3.15908200	0.99381300	2.85313700
C	-0.45240800	1.33104600	-0.00954300
H	-0.52354600	1.27288000	1.07634300
N	-0.75209900	0.32535500	-0.72849200
N	2.82830000	1.11676000	0.85342300
O	3.80409200	0.14399100	0.83886400
O	-1.13776100	-0.75799600	0.03551500
C	3.92015900	-0.47969100	-0.43506400
H	4.94686300	-0.84945300	-0.47229300
C	-0.85215700	-1.94866100	-0.67634800
H	-1.26127700	-1.86648900	-1.69061600
H	-1.40114200	-2.73116300	-0.14721300
C	0.61728500	-2.29031900	-0.74296200
C	3.33959400	-2.88846800	-0.98477700
C	1.02932400	-3.57470600	-1.09253700
C	1.58815700	-1.33018000	-0.51187700
C	2.94256200	-1.60545300	-0.63783300
C	2.37908700	-3.86891500	-1.20842200
H	0.29236100	-4.35151800	-1.27372100
H	1.30761100	-0.32791200	-0.24497500
H	2.68804600	-4.87400500	-1.47319700
H	4.39382200	-3.12784500	-1.08176700
H	3.79304700	0.28387700	-1.20899600

4-c

H (393 K, 1 atm) = -1107.754527 a.u.

H	0.84592100	3.83906700	-3.66663500
C	0.86625100	3.71282100	-2.59024700
C	0.90032200	3.38860300	0.18304000
C	0.16291600	2.67875400	-2.00481900
C	1.59792300	4.58731000	-1.79693300
C	1.60480500	4.42563700	-0.42211900
C	0.17459000	2.49849300	-0.62018200
H	-0.40276800	1.98212900	-2.61136400
H	2.15581200	5.39990000	-2.24809300
H	2.16490500	5.11129200	0.20382600
C	0.86073800	3.33841200	1.66964800
C	0.64158700	3.50651400	4.46370700
C	1.64169000	2.49593100	2.48449200
C	-0.00478900	4.24193400	2.28208400
C	-0.12499900	4.33302300	3.65961800
C	1.51069600	2.60834600	3.87468600
H	-0.60000100	4.88764200	1.64596900
H	-0.81286100	5.04713500	4.09723700
H	2.11545200	1.96334200	4.50337600
H	0.56669200	3.56045800	5.54340400
C	2.60385200	1.48545500	2.05165400
H	3.15908200	0.99381300	2.85313700
C	-0.45240800	1.33104600	-0.00954300
H	-0.52354600	1.27288000	1.07634300
N	-0.75209900	0.32535500	-0.72849200
N	2.82830000	1.11676000	0.85342300
O	3.80409200	0.14399100	0.83886400
O	-1.13776100	-0.75799600	0.03551500

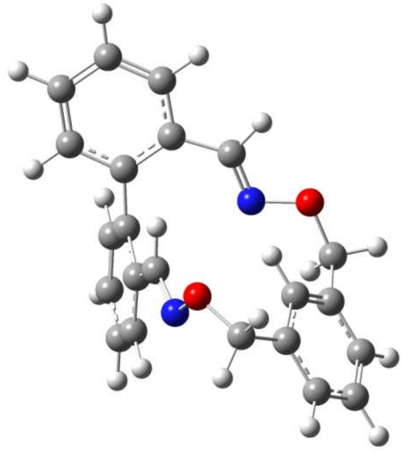

C	3.92015900	-0.47969100	-0.43506400
H	4.94686300	-0.84945300	-0.47229300
C	-0.85215700	-1.94866100	-0.67634800
H	-1.26127700	-1.86648900	-1.69061600
H	-1.40114200	-2.73116300	-0.14721300
C	0.61728500	-2.29031900	-0.74296200
C	3.33959400	-2.88846800	-0.98477700
C	1.02932400	-3.57470600	-1.09253700
C	1.58815700	-1.33018000	-0.51187700
C	2.94256200	-1.60545300	-0.63783300
C	2.37908700	-3.86891500	-1.20842200
H	0.29236100	-4.35151800	-1.27372100
H	1.30761100	-0.32791200	-0.24497500
H	2.68804600	-4.87400500	-1.47319700
H	4.39382200	-3.12784500	-1.08176700
H	3.79304700	0.28387700	-1.20899600

4-TS-rac

H (393 K, 1 atm) = -1107.711285 a.u.

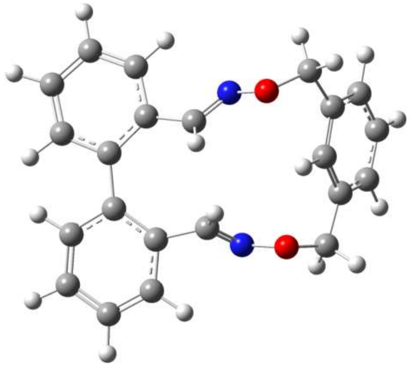

H	-2.49550700	4.04398000	-2.87539000
C	-1.80966900	3.92127500	-2.04581500
C	-0.00499600	3.52860000	0.16436500
C	-1.46416900	2.66590600	-1.60001400
C	-1.23697200	5.00033400	-1.40642600
C	-0.36618200	4.78794900	-0.35869400
C	-0.58421800	2.43075000	-0.52874300
H	-1.89185000	1.79928900	-2.08501400
H	-1.44591500	6.01629200	-1.72143800
H	0.07909200	5.68303800	0.03545600
C	1.02401200	3.55532700	1.27990300

C	3.28912800	4.02372300	2.97871700
C	1.72833000	2.48244000	1.89011100
C	1.42150600	4.83043500	1.73365600
C	2.50663600	5.07755400	2.54692600
C	2.86008800	2.75266300	2.67817700
H	0.85915600	5.70387800	1.45438000
H	2.73112400	6.09808500	2.83507700
H	3.37338500	1.89927500	3.09995300
H	4.16825000	4.17965300	3.59236900
C	1.32657500	1.06952000	1.98663500
H	0.28475100	0.82010100	2.17469100
C	-0.46917400	0.96825000	-0.31743700
H	-0.86290900	0.49368800	0.57941800
N	-0.17084100	0.26497200	-1.33622400
N	2.23360400	0.18351900	2.10071200
O	1.75617800	-1.02612700	2.54705100
O	-0.51530600	-1.06176900	-1.18043600
C	2.72399400	-2.02898900	2.26652200
H	2.39367300	-2.89580900	2.84543100
C	0.29663000	-1.87999600	-2.00545500
H	0.49255700	-1.36526200	-2.95071900
H	-0.30705200	-2.76981900	-2.20785900
C	1.57624000	-2.24412000	-1.30403100
C	3.90703900	-2.76767300	0.13189100
C	2.71638200	-2.68144500	-1.96712200
C	1.61341400	-2.10549600	0.07010700
C	2.76908900	-2.33168600	0.79546800
C	3.86914000	-2.95771700	-1.24427400
H	2.71648500	-2.78544000	-3.04764200
H	0.73077300	-1.75788200	0.58597500
H	4.76110100	-3.28899200	-1.76424000
H	4.83024900	-2.93457800	0.67765300
H	3.70391300	-1.71054900	2.63532500

4-d

H (393 K, 1 atm) = -1107.754527 a.u.

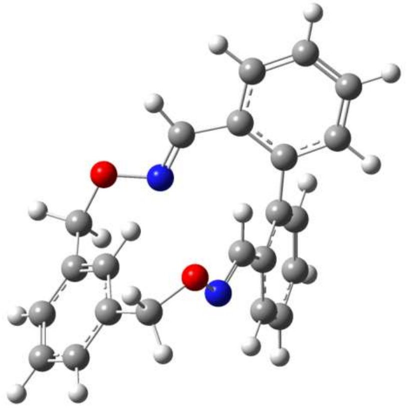

H	1.01017200	3.21387900	3.71968000
C	0.99541300	3.22583700	2.63587600
C	0.94767800	3.24663600	-0.15745100
C	0.33540500	2.22910000	1.94532300
C	1.64030000	4.23878900	1.93786700
C	1.60578500	4.24730200	0.55444400
C	0.30529300	2.22058600	0.54935200
H	-0.16315000	1.42662700	2.47491200
H	2.16496200	5.02396300	2.47020500
H	2.10147900	5.03873400	0.00334700
C	0.87107200	3.38474000	-1.63582400
C	0.57838000	3.89369800	-4.38438900
C	1.53529000	2.57186600	-2.57587100
C	0.08928400	4.43773100	-2.10683700
C	-0.06368700	4.69917200	-3.45901500
C	1.36434200	2.84866900	-3.93790200
H	-0.41911000	5.05924400	-1.37818100
H	-0.68592400	5.52523100	-3.78324800
H	1.87513500	2.22043500	-4.66007800
H	0.47056800	4.07668500	-5.44696700
C	2.41342700	1.43989400	-2.29524700
H	2.79221000	0.90833500	-3.17102600
C	-0.30193300	1.10744900	-0.17235700
H	-0.33903400	1.13913800	-1.26073800
N	-0.66932200	0.06292900	0.45026000
N	2.76360200	1.02861300	-1.14213700
O	3.59904700	-0.06150000	-1.26857300

O	-1.05658200	-0.94165400	-0.41917900
C	3.93017300	-0.60792100	-0.00810700
H	4.88409100	-1.11856100	-0.16451900
C	-0.81192700	-2.18961900	0.21213700
H	-1.48531900	-2.31491300	1.06797800
H	-1.07575700	-2.93038900	-0.54765100
C	0.61767400	-2.33349400	0.66787500
C	3.22226800	-2.35274400	1.64609500
C	0.94260200	-3.11058200	1.76987600
C	1.61828200	-1.60193700	0.03766100
C	2.90488400	-1.56131800	0.54561100
C	2.24960000	-3.14093600	2.23859400
H	0.17031200	-3.67551400	2.28248100
H	1.36753900	-1.00110100	-0.82338900
H	2.50146400	-3.75601600	3.09526400
H	4.22938800	-2.33961900	2.05195700
H	4.09235800	0.20967600	0.70288900

4-e

H (393 K, 1 atm) = -1107.763925 a.u.

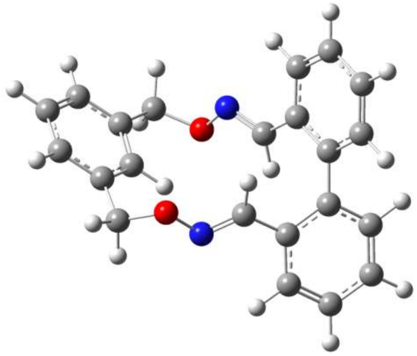

C	2.83222700	-0.41984700	-0.09757300
C	3.34774100	-2.94309600	-1.18041700
C	1.95339200	-1.48681000	0.15207600
C	3.96986700	-0.64711000	-0.86827200
C	4.22960500	-1.89515000	-1.40941500
C	2.22567400	-2.74032000	-0.39935800
H	4.64363000	0.18087500	-1.05946500
H	5.11772300	-2.04894700	-2.01153200
H	1.54446900	-3.55540400	-0.18730500
H	3.54457800	-3.92332900	-1.59902300
C	2.57369900	0.94443900	0.42387400

C	2.13352300	3.49317700	1.47921900
C	3.44412000	1.48673300	1.36705400
C	1.47670400	1.70755400	-0.01013900
C	1.27170700	2.97904100	0.53023100
C	3.22947100	2.74786500	1.89595400
H	4.28897800	0.89300600	1.69832400
H	0.42614000	3.55779100	0.17898200
H	3.91486700	3.14841000	2.63403700
H	1.95931500	4.48189600	1.88776900
C	0.57903300	1.18055400	-1.03607800
H	0.91085400	0.34066100	-1.64610600
C	0.78436600	-1.29287200	1.00930700
H	0.79840900	-0.50270200	1.75961300
N	-0.57873000	1.67744500	-1.20808700
N	-0.24116500	-2.03475900	0.87304600
O	-1.19425000	-1.77036000	1.82477100
O	-1.23914100	1.06267400	-2.25034600
C	-2.47618700	-2.21126400	1.38871500
H	-3.00432400	-2.52800000	2.29058700
H	-2.33845900	-3.07701900	0.73437500
C	-2.62438100	1.32630100	-2.15028000
H	-2.79110100	2.40593300	-2.06905200
H	-3.03514200	0.98506600	-3.10484700
C	-3.22875800	-1.10796700	0.69965700
C	-4.51232800	1.04172400	-0.51380600
C	-2.66299600	-0.47498200	-0.40219300
C	-4.45734000	-0.66915200	1.17389500
C	-5.10493700	0.39303000	0.55666800
C	-3.28090000	0.61202700	-0.99892100
H	-4.90372800	-1.14728200	2.03999000
H	-5.00277800	1.89818200	-0.96647300
H	-6.06256700	0.73481800	0.93301500
H	-1.70605800	-0.81373200	-0.77722700

1-Li$^+$-a

H (353 K, 1 atm) = -1038.036725 a.u.

H	0.97331700	2.01393700	-4.43377300
C	0.64796700	2.09288900	-3.40351900
C	-0.21437500	2.30713900	-0.74654700
C	-0.04662400	1.04985700	-2.81552100
C	0.89234800	3.25064900	-2.67881100
C	0.46389400	3.35311500	-1.36407000
C	-0.48809300	1.14690300	-1.49383500
H	-0.30492500	0.17001000	-3.39418800
H	1.42190900	4.07722900	-3.13747800
H	0.68180900	4.24651100	-0.78989500
C	-0.57587000	2.40150300	0.68506900
C	-1.30624000	2.54651900	3.37804000
C	-0.07930700	1.46748800	1.61127100
C	-1.41807900	3.41314200	1.13684200
C	-1.78206000	3.48560600	2.47154500
C	-0.45611700	1.54343900	2.95074600
H	-1.80638800	4.13370300	0.42591300
H	-2.44263300	4.27663800	2.80610100
H	-0.05455200	0.82267100	3.65272200
H	-1.58909700	2.60645200	4.42200000
C	0.85111300	0.44549500	1.13965000
H	1.51873100	0.70283300	0.31444300
C	-1.30299100	0.07043100	-0.92628000
H	-2.18751400	0.31129500	-0.33659600
N	-0.98949000	-1.14863600	-1.13239900
N	0.84685700	-0.72855400	1.62313900
O	1.73687600	-1.55939700	0.91523000
O	-1.91048400	-2.03057500	-0.61042800

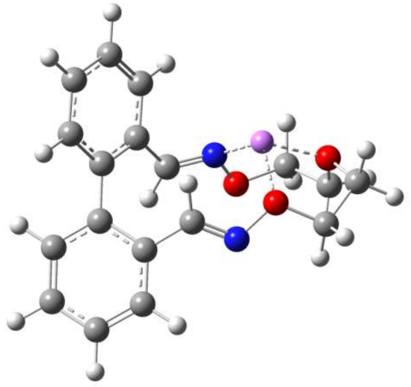

C	1.76727000	-2.85905600	1.51380600
H	2.66876200	-2.96769100	2.12263000
H	0.89328700	-2.94972900	2.16001100
C	1.75246500	-3.87094600	0.38923100
H	2.68254500	-3.83401500	-0.18308400
H	1.64502600	-4.88504800	0.78573500
C	-1.64010700	-3.36817000	-1.00394800
H	-1.38468900	-3.40454400	-2.06771700
H	-2.58645100	-3.89213900	-0.85990100
C	-0.58365700	-4.04744200	-0.16919800
H	-0.62262000	-5.12864900	-0.34172700
H	-0.77828900	-3.85101300	0.89040500
O	0.71043600	-3.56768500	-0.53627800
Li	0.90625500	-1.72416600	-0.93844400

1-Li^{+}-rac

H (353 K, 1 atm) = -1037.987773 a.u.

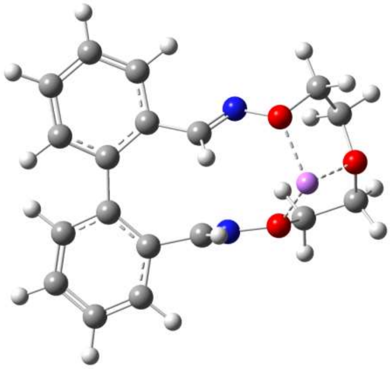

H	-1.93114800	0.06705500	0.35702900
C	-1.41939500	0.03270400	1.31074000
C	-0.14554400	-0.10909300	3.86372600
C	-0.94232800	-1.16220200	1.80642400
C	-1.14289200	1.18412400	2.02286100
C	-0.52422200	1.09891300	3.25563300
C	-0.26986900	-1.25333500	3.03232100
H	-1.02661300	-2.06266500	1.21185300
H	-1.40488700	2.15783800	1.62644800
H	-0.31752400	2.03050600	3.75940900
C	0.25082100	-0.01821800	5.31266500
C	0.93927700	0.56726800	8.02932300
C	0.93266300	-0.94782000	6.12782200

C	-0.16137600	1.14741200	5.98642100
C	0.17419600	1.44957300	7.29088100
C	1.28038600	-0.63414300	7.44355900
H	-0.81297000	1.84506400	5.48436900
H	-0.18223900	2.37419200	7.72887200
H	1.81454500	-1.37781600	8.02415100
H	1.22291900	0.78231600	9.05199800
C	1.28334700	-2.34933200	5.80980000
H	2.25845400	-2.59996300	5.38126600
C	0.45592000	-2.51839600	3.17231700
H	1.52515800	-2.46108300	3.37978600
N	-0.06768100	-3.62645500	2.83464200
N	0.48018600	-3.24095500	6.21160000
O	0.94621200	-4.54212300	5.97641300
O	0.91114100	-4.64695400	2.74572600
C	-0.08190100	-5.46329700	6.37034100
H	-0.16214300	-5.48886400	7.45967100
H	-1.02940700	-5.10859000	5.96051400
C	0.31104500	-6.82062800	5.83302300
H	1.17431500	-7.22036700	6.36886800
H	-0.51701700	-7.52518400	5.95362100
C	0.28624500	-5.86464600	2.32464500
H	1.08949400	-6.46396600	1.88941900
H	-0.45842600	-5.65027500	1.55764600
C	-0.32970300	-6.56427600	3.50909300
H	-0.73323800	-7.54025100	3.22335400
H	-1.13594100	-5.94950600	3.92000600
O	0.71842700	-6.73145400	4.46906300
Li	1.80462500	-5.16221900	4.36792200

1-Li+-c

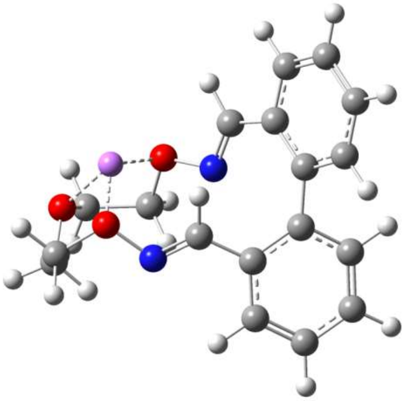

H (353 K, 1 atm) = -1038.023442 a.u.

H	-3.12262600	1.59304200	-3.35874000
C	-2.45113200	1.69961600	-2.51542800
C	-0.68280200	1.99916100	-0.35556600
C	-1.33441500	0.89402800	-2.41303500
C	-2.70021000	2.64872600	-1.53422700
C	-1.82058900	2.79679500	-0.47561600
C	-0.45163000	1.02586300	-1.34054800
H	-1.12142400	0.15317500	-3.17397200
H	-3.57560300	3.28418200	-1.59763500
H	-2.01652600	3.54320400	0.28544200
C	0.23942900	2.31457000	0.77003800
C	1.90754300	3.25462600	2.84538600
C	0.59992600	1.46578400	1.84108900
C	0.73993400	3.61570700	0.78252100
C	1.56187000	4.08698600	1.79443100
C	1.42874200	1.95968300	2.85684200
H	0.47672400	4.27291900	-0.03806900
H	1.92977900	5.10545200	1.75630600
H	1.69411400	1.30246200	3.67832800
H	2.54409800	3.60801200	3.64702900
C	0.23292900	0.06641100	2.00332000
H	0.69279200	-0.45466500	2.84795300
C	0.66216700	0.09722200	-1.20259600
H	1.52141000	0.38660000	-0.59425200
N	0.57977700	-1.06212000	-1.71916500
N	-0.51728200	-0.58324900	1.20431000
O	-0.47538300	-1.96161100	1.50163700

O	1.67521700	-1.87652500	-1.35009200
C	-1.48473200	-2.66095200	0.75804400
H	-2.40628700	-2.71735800	1.34307200
H	-1.67630600	-2.10330200	-0.16206300
C	-0.92987600	-4.03926000	0.48044000
H	-0.75224200	-4.58168100	1.41120900
H	-1.63260000	-4.62013000	-0.12261700
C	1.55316300	-3.12895500	-2.03539300
H	2.45837100	-3.67908400	-1.76936600
H	1.54041800	-2.96509800	-3.11474300
C	0.31020500	-3.86721500	-1.59500400
H	0.29928100	-4.87668100	-2.01731800
H	-0.57920200	-3.32636100	-1.92771500
O	0.33971300	-3.93528400	-0.16750700
Li	1.19317000	-2.37171000	0.51225100

1-Li$^+$-c

H (353 K, 1 atm) = -1038.036725 a.u.

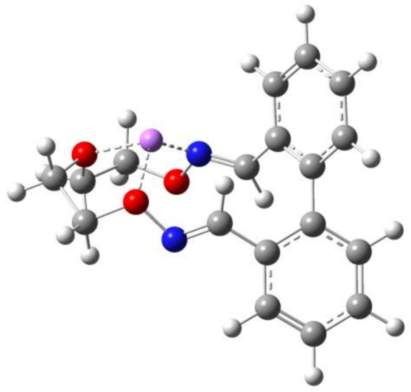

H	0.97331700	2.01393700	4.43377300
C	0.64796700	2.09288900	3.40351900
C	-0.21437500	2.30713900	0.74654700
C	-0.04662400	1.04985700	2.81552100
C	0.89234800	3.25064900	2.67881100
C	0.46389400	3.35311500	1.36407000
C	-0.48809300	1.14690300	1.49383500
H	-0.30492500	0.17001000	3.39418800
H	1.42190900	4.07722900	3.13747800
H	0.68180900	4.24651100	0.78989500
C	-0.57587000	2.40150300	-0.68506900
C	-1.30624000	2.54651900	-3.37804000
C	-0.07930700	1.46748800	-1.61127100

C	-1.41807900	3.41314200	-1.13684200
C	-1.78206000	3.48560600	-2.47154500
C	-0.45611700	1.54343900	-2.95074600
H	-1.80638800	4.13370300	-0.42591300
H	-2.44263300	4.27663800	-2.80610100
H	-0.05455200	0.82267100	-3.65272200
H	-1.58909700	2.60645200	-4.42200000
C	0.85111300	0.44549500	-1.13965000
H	1.51873100	0.70283300	-0.31444300
C	-1.30299100	0.07043100	0.92628000
H	-2.18751400	0.31129500	0.33659600
N	-0.98949000	-1.14863600	1.13239900
N	0.84685700	-0.72855400	-1.62313900
O	1.73687600	-1.55939700	-0.91523000
O	-1.91048400	-2.03057500	0.61042800
C	1.76727000	-2.85905600	-1.51380600
H	2.66876200	-2.96769100	-2.12263000
H	0.89328700	-2.94972900	-2.16001100
C	1.75246500	-3.87094600	-0.38923100
H	2.68254500	-3.83401500	0.18308400
H	1.64502600	-4.88504800	-0.78573500
C	-1.64010700	-3.36817000	1.00394800
H	-1.38468900	-3.40454400	2.06771700
H	-2.58645100	-3.89213900	0.85990100
C	-0.58365700	-4.04744200	0.16919800
H	-0.62262000	-5.12864900	0.34172700
H	-0.77828900	-3.85101300	-0.89040500
O	0.71043600	-3.56768500	0.53627800
Li	0.90625500	-1.72416600	0.93844400

1-Na$^+$-a

H (353 K, 1 atm) = -1192.722415 a.u.

H	0.51964900	-0.19852800	0.05815600
C	0.39699100	-0.17645500	1.13449600
C	0.03523800	-0.09395700	3.91128100
C	-0.31654900	-1.17861800	1.77094900
C	0.91105600	0.87846300	1.87575800
C	0.72401100	0.91810700	3.24896400
C	-0.50285200	-1.15249200	3.15707800
H	-0.79720300	-1.95946900	1.19058400
H	1.45429400	1.67592200	1.38288000
H	1.13840300	1.73420200	3.83009400
C	-0.08919400	-0.06766000	5.38665000
C	-0.41467800	-0.06626700	8.15931300
C	0.47962600	-1.08549400	6.16978800
C	-0.79409900	0.95370300	6.01513300
C	-0.95704400	0.95564600	7.39142100
C	0.30487600	-1.07953800	7.55206500
H	-1.23728500	1.73859400	5.41256600
H	-1.51258300	1.75582000	7.86583700
H	0.76061700	-1.86483000	8.14289800
H	-0.54065000	-0.06376700	9.23526000
C	1.28413900	-2.11574800	5.51490800
H	1.88830400	-1.82139400	4.65218700
C	-1.29150800	-2.20227000	3.80973200
H	-1.94188400	-1.95571800	4.64813700
N	-1.19186100	-3.40413100	3.39627000
N	1.26513800	-3.32088200	5.91379700
O	2.06244100	-4.14065300	5.11625200
O	-2.02176300	-4.26775900	4.05681400

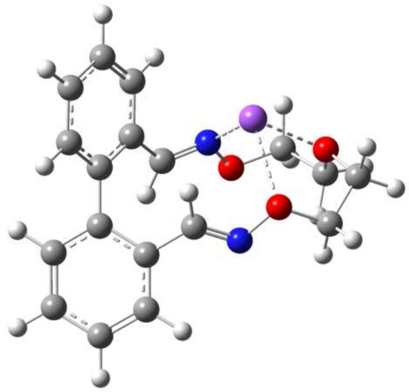

C	1.99480000	-5.48830400	5.58564400
H	2.95564600	-5.76836400	6.02727700
H	1.22925300	-5.53525500	6.36026300
C	1.67659700	-6.39459600	4.41610100
H	2.50104000	-6.39452800	3.69694300
H	1.55457800	-7.42536900	4.76796500
C	-1.85225800	-5.60711900	3.61902000
H	-1.75097000	-5.63732300	2.52958800
H	-2.78514500	-6.10335000	3.89189000
C	-0.71056300	-6.32852400	4.29152300
H	-0.85151200	-7.41021600	4.17533700
H	-0.71972500	-6.08837300	5.36035300
O	0.53011100	-5.95692400	3.70210400
Na	1.04502500	-3.88829700	2.85416800

1-Na^{+}-b

H (353 K, 1 atm) = -1192.703607 a.u.

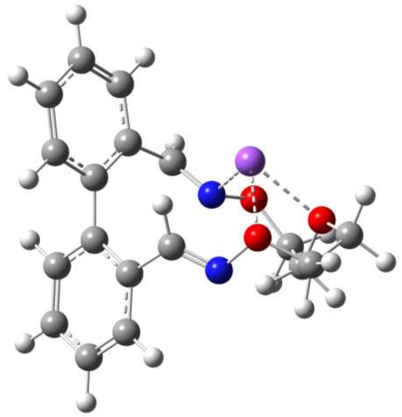

H	0.99526900	2.98561400	-4.46582200
C	0.67764500	2.76755800	-3.45338000
C	-0.19006800	2.21361800	-0.84153700
C	-0.18352800	1.71262500	-3.20999700
C	1.11301100	3.54934600	-2.39410400
C	0.66919700	3.27827200	-1.10965900
C	-0.60284100	1.40312400	-1.91147000
H	-0.54294800	1.11046200	-4.03865300
H	1.77863800	4.38617800	-2.56925600
H	0.97509000	3.91502500	-0.28711500
C	-0.70520100	2.07110000	0.54761900
C	-1.69630900	1.94224000	3.15735700
C	-0.07728400	1.26665400	1.50564400

C	-1.83196700	2.79907200	0.91600600
C	-2.32748400	2.73538300	2.20859400
C	-0.57833100	1.20984600	2.80621300
H	-2.32002400	3.42459200	0.17719000
H	-3.20246700	3.31501100	2.47792000
H	-0.07203100	0.59118200	3.53720400
H	-2.07234900	1.90244000	4.17253300
C	1.04245800	0.42445700	1.09791600
H	1.69692200	0.76692300	0.28996000
C	-1.46208100	0.21237800	-1.79390900
H	-2.27913500	0.08686800	-2.50717700
N	-1.18985500	-0.73200600	-0.98232000
N	1.18211500	-0.72033400	1.62965700
O	2.19296000	-1.48381800	1.05266100
O	-1.97795600	-1.83537600	-1.21104400
C	2.09593500	-2.81515400	1.57175600
H	3.10872800	-3.15808400	1.79442500
H	1.53032900	-2.76614200	2.50190400
C	1.45377300	-3.75802400	0.56023800
H	2.16464900	-4.00784200	-0.23238900
H	1.18931300	-4.69510200	1.06577300
C	-2.04466000	-2.76465100	-0.13215500
H	-2.31480500	-3.69932000	-0.62597500
H	-2.85796100	-2.47848100	0.54475900
C	-0.80399600	-2.94229200	0.70854800
H	-0.99409600	-3.79285500	1.37541000
H	-0.61819600	-2.06031500	1.32710600
O	0.32923600	-3.21284500	-0.11060600
Na	1.10356000	-1.33775300	-1.17210700

1-Na$^+$-rac

H (353 K, 1 atm) = -1192.671347 a.u.

H	-1.96061800	0.04449900	0.37379500
C	-1.44283600	0.01460700	1.32446200
C	-0.15515500	-0.11954000	3.87038700
C	-0.94995600	-1.17540600	1.81580600
C	-1.17279200	1.16821700	2.03561700
C	-0.54661100	1.08616400	3.26431900
C	-0.26991400	-1.26440800	3.03803900
H	-1.02840400	-2.07586700	1.22057200
H	-1.44512400	2.14014500	1.64174800
H	-0.34422600	2.01912600	3.76712500
C	0.24641100	-0.02215300	5.31792100
C	0.94975700	0.57985100	8.02780100
C	0.93512100	-0.94602500	6.13360500
C	-0.16759500	1.14444800	5.98982100
C	0.17485600	1.45461000	7.29044000
C	1.29091000	-0.62284100	7.44509700
H	-0.82658100	1.83639700	5.48972500
H	-0.18397900	2.37926500	7.72647400
H	1.82959000	-1.36276500	8.02645000
H	1.23919600	0.80103600	9.04757500
C	1.28179400	-2.35041000	5.82366000
H	2.26148100	-2.59971600	5.40255600
C	0.46601300	-2.52617300	3.17139900
H	1.53263700	-2.46191600	3.39042100
N	-0.04432200	-3.63150900	2.80701900
N	0.48065500	-3.24316100	6.22558200

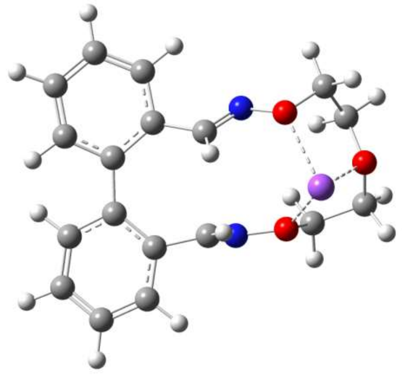

O	0.94439100	-4.53831400	6.01471200
O	0.92360100	-4.64176200	2.68854000
C	-0.11272300	-5.44497800	6.34366400
H	-0.25079700	-5.48307200	7.42794700
H	-1.03424000	-5.06932600	5.89586900
C	0.26060100	-6.81393700	5.82293600
H	1.10611000	-7.22456800	6.38096500
H	-0.58633100	-7.49423700	5.96480900
C	0.28003100	-5.86101100	2.31954700
H	1.06868100	-6.48783100	1.89377000
H	-0.46641700	-5.66456600	1.54795900
C	-0.35232300	-6.52773500	3.51332800
H	-0.83120400	-7.46732000	3.21720800
H	-1.11333800	-5.86188100	3.92940700
O	0.67646200	-6.78492200	4.46520500
Na	2.40005500	-5.26811700	4.35194800

1-Na^{+}-c

H (353 K, 1 atm) = -1192,719114 a.u.

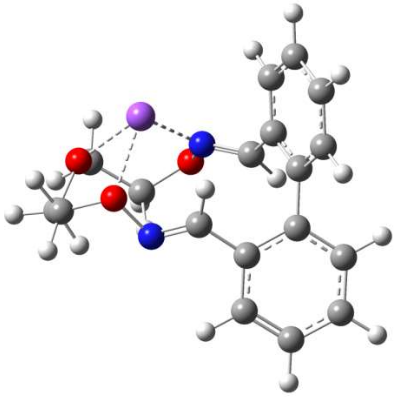

H	-3.73221300	1.95504100	-3.09193800
C	-2.95126600	2.05463200	-2.34776700
C	-0.93897700	2.32394000	-0.43301600
C	-1.87029300	1.19211200	-2.36321800
C	-3.02398500	3.06037100	-1.39258400
C	-2.02324600	3.19367500	-0.44232000
C	-0.85633100	1.32132000	-1.41587900
H	-1.78497200	0.42614200	-3.12469800
H	-3.86481100	3.74375500	-1.38693100
H	-2.08852800	3.96756200	0.31417900
C	0.11048300	2.41804400	0.61043300
C	2.22619800	2.46674400	2.44293500

C	0.25794500	1.38817000	1.55377300
C	1.01421500	3.47367000	0.62509900
C	2.06388300	3.50076600	1.53344600
C	1.31941900	1.41687400	2.45870900
H	0.90601900	4.26802100	-0.10484500
H	2.75869700	4.33216500	1.52706200
H	1.38981500	0.64369400	3.21716100
H	3.03888700	2.49156000	3.15902900
C	-0.74103800	0.31308100	1.63045000
H	-1.79789600	0.55902900	1.73761400
C	0.32371100	0.46311500	-1.43518300
H	1.25769400	0.88120600	-1.05160100
N	0.29038700	-0.74018700	-1.84246100
N	-0.37276600	-0.90361100	1.58536900
O	-1.42620200	-1.76632000	1.84569300
O	1.54358100	-1.34165400	-1.68235200
C	-1.52229400	-2.82821500	0.90762900
H	-2.40690800	-3.37289300	1.24228000
H	-1.73275500	-2.41844500	-0.08694300
C	-0.34702000	-3.78487600	0.86237600
H	0.05375000	-3.92873100	1.86835900
H	-0.69441400	-4.75995300	0.50182000
C	1.49849500	-2.69713100	-2.12141200
H	2.51773000	-3.06076300	-1.97265300
H	1.26337900	-2.74306300	-3.18805000
C	0.50032500	-3.51944600	-1.34374800
H	0.61042000	-4.57678500	-1.61451000
H	-0.51109300	-3.19798200	-1.60451800
O	0.73333900	-3.34073700	0.04565400
Na	1.76852700	-1.41361200	0.71869100

1-Na$^+$-d

H (353 K, 1 atm) = -1192.722415 a.u.

H	0.51964900	-0.19852800	-0.05815600
C	0.39699100	-0.17645500	-1.13449600
C	0.03523800	-0.09395700	-3.91128100
C	-0.31654900	-1.17861800	-1.77094900
C	0.91105600	0.87846300	-1.87575800
C	0.72401100	0.91810700	-3.24896400
C	-0.50285200	-1.15249200	-3.15707800
H	-0.79720300	-1.95946900	-1.19058400
H	1.45429400	1.67592200	-1.38288000
H	1.13840300	1.73420200	-3.83009400
C	-0.08919400	-0.06766000	-5.38665000
C	-0.41467800	-0.06626700	-8.15931300
C	0.47962600	-1.08549400	-6.16978800
C	-0.79409900	0.95370300	-6.01513300
C	-0.95704400	0.95564600	-7.39142100
C	0.30487600	-1.07953800	-7.55206500
H	-1.23728500	1.73859400	-5.41256600
H	-1.51258300	1.75582000	-7.86583700
H	0.76061700	-1.86483000	-8.14289800
H	-0.54065000	-0.06376700	-9.23526000
C	1.28413900	-2.11574800	-5.51490800
H	1.88830400	-1.82139400	-4.65218700
C	-1.29150800	-2.20227000	-3.80973200
H	-1.94188400	-1.95571800	-4.64813700
N	-1.19186100	-3.40413100	-3.39627000
N	1.26513800	-3.32088200	-5.91379700
O	2.06244100	-4.14065300	-5.11625200

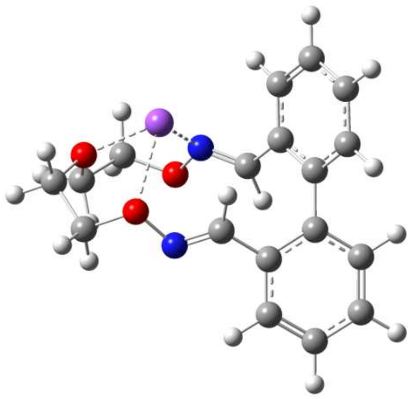

O	-2.02176300	-4.26775900	-4.05681400
C	1.99480000	-5.48830400	-5.58564400
H	2.95564600	-5.76836400	-6.02727700
H	1.22925300	-5.53525500	-6.36026300
C	1.67659700	-6.39459600	-4.41610100
H	2.50104000	-6.39452800	-3.69694300
H	1.55457800	-7.42536900	-4.76796500
C	-1.85225800	-5.60711900	-3.61902000
H	-1.75097000	-5.63732300	-2.52958800
H	-2.78514500	-6.10335000	-3.89189000
C	-0.71056300	-6.32852400	-4.29152300
H	-0.85151200	-7.41021600	-4.17533700
H	-0.71972500	-6.08837300	-5.36035300
O	0.53011100	-5.95692400	-3.70210400
Na	1.04502500	-3.88829700	-2.85416800

1-K$^+$-a

H (353 K, 1 atm) = -1630.295581 a.u.

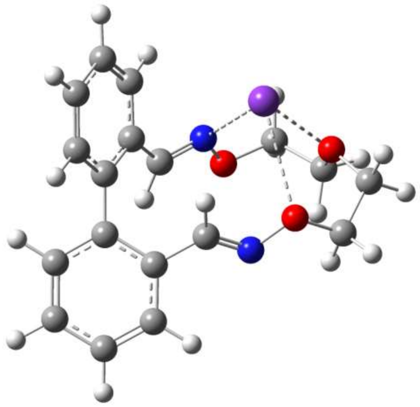

H	0.34424000	-0.08813800	0.03552900
C	0.28051200	-0.10206700	1.11734000
C	0.04511300	-0.09936700	3.90990700
C	-0.39798700	-1.12438500	1.75897000
C	0.83254800	0.93057200	1.86392400
C	0.70347700	0.93194500	3.24358700
C	-0.51324100	-1.14588300	3.15332100
H	-0.90759600	-1.88921700	1.18171200
H	1.35097700	1.74366800	1.36981500
H	1.13343300	1.73784300	3.82768100
C	-0.05940600	-0.07330400	5.38745900
C	-0.38191400	-0.04106300	8.16124400

C	0.49995500	-1.08919700	6.17862400
C	-0.75263200	0.96129100	6.00905100
C	-0.91438100	0.97934000	7.38503500
C	0.32664900	-1.06622800	7.56127700
H	-1.18996700	1.74413500	5.39949900
H	-1.46123100	1.78992800	7.85179700
H	0.77537700	-1.85103000	8.15801800
H	-0.50628700	-0.02837300	9.23734400
C	1.29715700	-2.13799900	5.54357200
H	1.92104400	-1.86176100	4.68965400
C	-1.24467500	-2.23051600	3.81301300
H	-1.80732500	-2.02895400	4.72354000
N	-1.19483200	-3.40877500	3.32832300
N	1.25758000	-3.33475100	5.96476300
O	2.07105200	-4.17943000	5.22687600
O	-1.97135300	-4.29070700	4.02802600
C	1.92398900	-5.51993100	5.69024900
H	2.84255900	-5.83321700	6.19658600
H	1.10701900	-5.54049300	6.41188900
C	1.65685500	-6.42394700	4.50747500
H	2.52000200	-6.42832000	3.83402100
H	1.52140400	-7.45483800	4.85678100
C	-1.82044100	-5.62237900	3.56621900
H	-1.68573100	-5.63020000	2.48031900
H	-2.76906900	-6.11036100	3.79884200
C	-0.71229700	-6.37293900	4.26105000
H	-0.85904500	-7.45072000	4.11202800
H	-0.76638100	-6.16193100	5.33484800
O	0.54999800	-5.99346800	3.73576300
K	1.38736100	-3.83654500	2.38247300

1-K$^+$-a

H (353 K, 1 atm) = -1630.276383 a.u.

H	0.39744200	2.98583600	-4.54078400
C	0.17958200	2.84508200	-3.48882700
C	-0.43748300	2.48419900	-0.78037600
C	-0.32261000	1.63834800	-3.04378900
C	0.37320900	3.88287500	-2.58694900
C	0.05943400	3.69973200	-1.25248100
C	-0.62831800	1.43446000	-1.69329300
H	-0.52327400	0.83990200	-3.74724400
H	0.75929600	4.83729700	-2.92478100
H	0.21342800	4.50606400	-0.54458300
C	-0.75218600	2.38087000	0.66128600
C	-1.39078200	2.31903600	3.38369400
C	-0.14693300	1.44022000	1.50730300
C	-1.66814800	3.28205500	1.20450000
C	-1.99193900	3.25221200	2.54897500
C	-0.47430600	1.42540500	2.86574300
H	-2.14024600	4.00483000	0.54874200
H	-2.70892100	3.96036600	2.94709800
H	0.02035100	0.71144500	3.51222000
H	-1.62680700	2.29931700	4.44087900
C	0.82022200	0.46909700	0.98638900
H	1.42360800	0.71362400	0.11138500
C	-1.18336200	0.13388000	-1.28700300
H	-1.85660700	0.08190000	-0.43207800
N	-0.84898200	-0.87158400	-1.99520700
N	0.88965400	-0.65467000	1.57205400
O	1.79459000	-1.55296200	1.03487500
O	-1.37540300	-2.12569500	-1.81847500

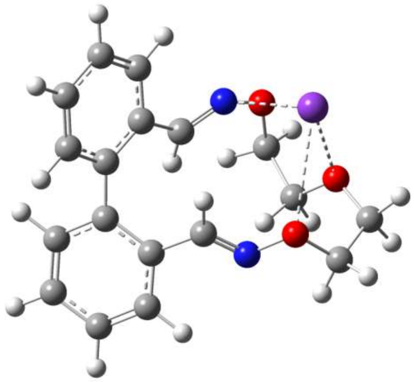

C	1.65749700	-2.77723600	1.75918900
H	2.65099200	-3.09434700	2.08697100
H	1.05564400	-2.56680300	2.64239200
C	1.03077000	-3.87984300	0.90515700
H	1.79372500	-4.37510900	0.29662100
H	0.61507200	-4.64583100	1.57283700
C	-2.04674900	-2.52763500	-0.62596300
H	-2.54237300	-3.44553100	-0.94347400
H	-2.81898800	-1.81554200	-0.31984900
C	-1.08948400	-2.80932200	0.52077900
H	-1.59122000	-3.47832000	1.23216000
H	-0.81666000	-1.89497400	1.05402400
O	0.06572100	-3.42939200	-0.01968000
K	1.47447000	-2.21827100	-2.00648400

1-K^{+}-rac

H (353 K, 1 atm) = -1630.244987 a.u.

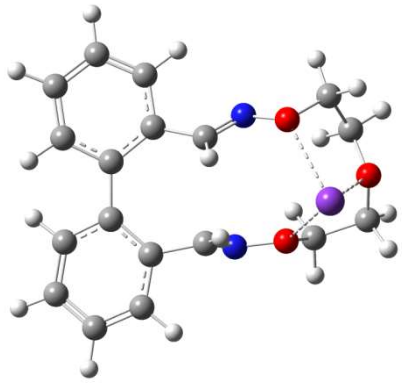

H	-1.99102800	0.02926000	0.38543900
C	-1.46591100	0.00552500	1.33230600
C	-0.16022000	-0.11441200	3.86996500
C	-0.96110500	-1.17944900	1.82338000
C	-1.19853500	1.16280700	2.03856300
C	-0.56369100	1.08728200	3.26290500
C	-0.27171600	-1.26212600	3.04083800
H	-1.03827100	-2.08247700	1.23188500
H	-1.48012900	2.13210400	1.64466700
H	-0.36511000	2.02351100	3.76070500
C	0.24849100	-0.01350300	5.31605700
C	0.94610600	0.57653700	8.03116400
C	0.91867600	-0.94848700	6.13502100
C	-0.14529400	1.16189200	5.98511900
C	0.19535300	1.46669100	7.28740300

C	1.26929500	-0.63152700	7.44967900
H	-0.78818800	1.86711100	5.48318400
H	-0.14772200	2.39896300	7.71998400
H	1.79016500	-1.38179500	8.03385700
H	1.23099000	0.79161000	9.05357400
C	1.25580700	-2.35395600	5.82043400
H	2.22544100	-2.60396600	5.38000700
C	0.47133800	-2.52130100	3.17254400
H	1.53808300	-2.45373500	3.38607700
N	-0.03682900	-3.62787700	2.80968300
N	0.46284100	-3.24817500	6.23547600
O	0.92278900	-4.53857200	6.02002700
O	0.92445900	-4.63470500	2.68691200
C	-0.13936200	-5.43921400	6.33410800
H	-0.29268900	-5.48101300	7.41701400
H	-1.05631700	-5.06066300	5.87908000
C	0.22805000	-6.81107500	5.81883900
H	1.06849800	-7.22234900	6.38523000
H	-0.62350800	-7.48501100	5.97103600
C	0.27517400	-5.84956900	2.32619800
H	1.05920300	-6.48701100	1.90658000
H	-0.46625100	-5.65612300	1.54801100
C	-0.37091600	-6.50863100	3.51587200
H	-0.87851700	-7.43064900	3.20926400
H	-1.11524600	-5.82486100	3.93269700
O	0.64475200	-6.80330800	4.46518800
K	2.94391500	-5.41485000	4.33927000

1-K$^+$-c

H (353 K, 1 atm) = -1630.290979 a.u.

H	-3.34910500	2.03371900	-3.58457200
C	-2.68932100	2.13215000	-2.73093500
C	-0.96473500	2.39636100	-0.54015600
C	-1.63520100	1.25550300	-2.56919200
C	-2.88684000	3.14990400	-1.80632800
C	-2.02970700	3.27800200	-0.72832200
C	-0.76519400	1.37217500	-1.48173400
H	-1.45287100	0.47643600	-3.29902600
H	-3.70861600	3.84574200	-1.92710300
H	-2.19107700	4.06361600	0.00101600
C	-0.09144700	2.59583800	0.63878600
C	1.57163600	3.09544000	2.83849800
C	0.14144700	1.59598100	1.59725100
C	0.51802300	3.83838700	0.81339000
C	1.34273500	4.08914100	1.89578800
C	0.97232200	1.85988300	2.68838800
H	0.35007800	4.60992000	0.07067900
H	1.80495500	5.06294100	2.00621800
H	1.11286900	1.08762500	3.43492300
H	2.20258000	3.29115400	3.69718700
C	-0.49407400	0.28066100	1.47603400
H	-1.48736600	0.21828200	1.03464900
C	0.34973800	0.43392300	-1.34392000
H	1.23688300	0.73669000	-0.78640200
N	0.24874400	-0.72748100	-1.84975000
N	0.15567400	-0.73899400	1.87359700
O	-0.39587000	-1.99867600	1.77180700
O	1.35915500	-1.52546900	-1.61681300

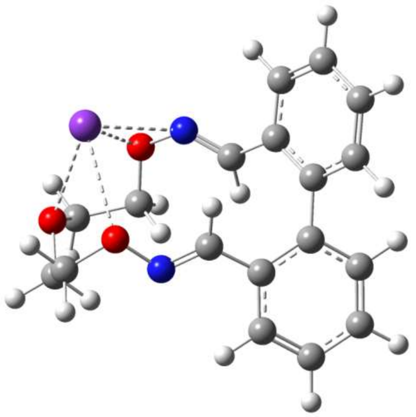

C	-1.43637600	-2.27077600	0.82853300
H	-2.41901200	-2.18690600	1.30375800
H	-1.37864800	-1.57578200	-0.01475900
C	-1.16320300	-3.68008400	0.35230600
H	-1.06831900	-4.34964400	1.21019900
H	-1.99462500	-4.03982000	-0.26240400
C	1.15327000	-2.77828400	-2.26858500
H	2.06838000	-3.34729200	-2.08260400
H	1.06083600	-2.62769700	-3.34809200
C	-0.06206700	-3.50893600	-1.75501500
H	-0.15849100	-4.46398600	-2.28544000
H	-0.94840600	-2.90526100	-1.96723000
O	0.06325500	-3.73709900	-0.35955900
K	2.16841100	-2.38602300	0.99669400

1-K^{+}-d

H (353 K, 1 atm) = -1630.295581 a.u.

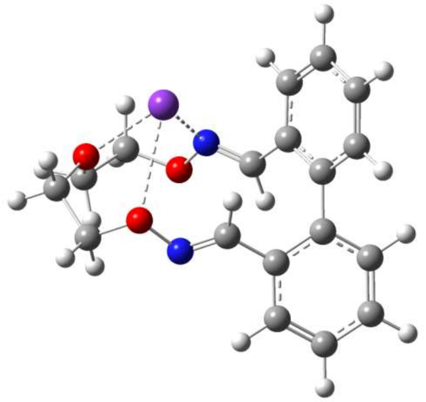

H	0.34424000	-0.08813800	-0.03552900
C	0.28051200	-0.10206700	-1.11734000
C	0.04511300	-0.09936700	-3.90990700
C	-0.39798700	-1.12438500	-1.75897000
C	0.83254800	0.93057200	-1.86392400
C	0.70347700	0.93194500	-3.24358700
C	-0.51324100	-1.14588300	-3.15332100
H	-0.90759600	-1.88921700	-1.18171200
H	1.35097700	1.74366800	-1.36981500
H	1.13343300	1.73784300	-3.82768100
C	-0.05940600	-0.07330400	-5.38745900
C	-0.38191400	-0.04106300	-8.16124400
C	0.49995500	-1.08919700	-6.17862400

C	-0.75263200	0.96129100	-6.00905100
C	-0.91438100	0.97934000	-7.38503500
C	0.32664900	-1.06622800	-7.56127700
H	-1.18996700	1.74413500	-5.39949900
H	-1.46123100	1.78992800	-7.85179700
H	0.77537700	-1.85103000	-8.15801800
H	-0.50628700	-0.02837300	-9.23734400
C	1.29715700	-2.13799900	-5.54357200
H	1.92104400	-1.86176100	-4.68965400
C	-1.24467500	-2.23051600	-3.81301300
H	-1.80732500	-2.02895400	-4.72354000
N	-1.19483200	-3.40877500	-3.32832300
N	1.25758000	-3.33475100	-5.96476300
O	2.07105200	-4.17943000	-5.22687600
O	-1.97135300	-4.29070700	-4.02802600
C	1.92398900	-5.51993100	-5.69024900
H	2.84255900	-5.83321700	-6.19658600
H	1.10701900	-5.54049300	-6.41188900
C	1.65685500	-6.42394700	-4.50747500
H	2.52000200	-6.42832000	-3.83402100
H	1.52140400	-7.45483800	-4.85678100
C	-1.82044100	-5.62237900	-3.56621900
H	-1.68573100	-5.63020000	-2.48031900
H	-2.76906900	-6.11036100	-3.79884200
C	-0.71229700	-6.37293900	-4.26105000
H	-0.85904500	-7.45072000	-4.11202800
H	-0.76638100	-6.16193100	-5.33484800
O	0.54999800	-5.99346800	-3.73576300
K	1.38736100	-3.83654500	-2.38247300

2-Li⁺-a

H (353 K, 1 atm) = -1191.711135 a.u.

H	-4.69179500	2.23069700	2.18453200
C	-3.60972800	2.18012100	2.19113200
C	-0.80921400	2.03916300	2.22477300
C	-2.96928400	1.03547800	1.76240200
C	-2.85971000	3.25987300	2.64065200
C	-1.47887200	3.18486000	2.65679600
C	-1.57475500	0.94871200	1.77545800
H	-3.54135600	0.17929900	1.42699500
H	-3.35307800	4.16359000	2.97844400
H	-0.89592000	4.03552800	2.99043700
C	0.67039200	2.03296400	2.27480300
C	3.46505200	2.06893700	2.50419400
C	1.48260000	1.71845600	1.17101600
C	1.29090400	2.36755700	3.47927100
C	2.66865900	2.38522500	3.59789300
C	2.87358800	1.73834800	1.30208900
H	0.67210000	2.59654900	4.33916700
H	3.12336800	2.64516100	4.54652100
H	3.48175400	1.50782100	0.43618500
H	4.54475300	2.08958500	2.58957800
C	-0.92276200	-0.28276000	1.33455100
H	0.07764500	-0.52015600	1.69594300
C	0.88270800	1.38416100	-0.11911200
H	-0.12135200	1.73792700	-0.35256800
N	-1.51291800	-1.05324500	0.51128400
N	1.52258300	0.66008200	-0.94733800
O	-0.68421900	-2.13722500	0.14294800

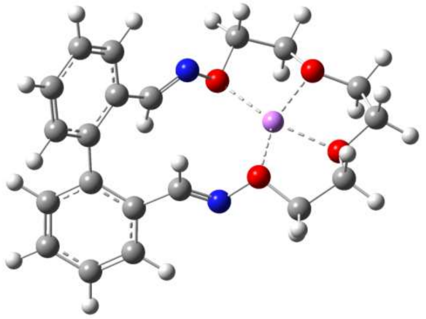

O	0.73979400	0.37777300	-2.08979700
C	1.59069700	0.35649200	-3.23869600
H	2.57962800	0.01011600	-2.92844300
H	1.67695200	1.36309500	-3.65719400
C	0.98482200	-0.59275300	-4.23328000
H	1.63773800	-0.68073400	-5.10873100
H	-0.00195700	-0.24706900	-4.56798400
O	0.86177900	-1.83232000	-3.55916800
C	0.23148800	-2.87329200	-4.28440600
H	0.86414600	-3.21816900	-5.10917300
H	-0.72302800	-2.51974600	-4.69428900
C	-0.00072800	-3.97970500	-3.28980900
H	0.95575000	-4.39366600	-2.94646700
H	-0.59463700	-4.78481100	-3.73535300
O	-0.68643400	-3.38111700	-2.20428300
C	-0.82629800	-4.17924300	-1.04259400
H	0.15961900	-4.51687100	-0.69732100
H	-1.44636700	-5.06079200	-1.23965000
C	-1.49513500	-3.30328100	-0.02146900
H	-2.48236800	-2.98993300	-0.36989900
H	-1.59931700	-3.82270500	0.93518100
Li	0.05258300	-1.56977400	-1.73554100

2-Li^+-b

H (353 K, 1 atm) = -1191.705465 a.u.

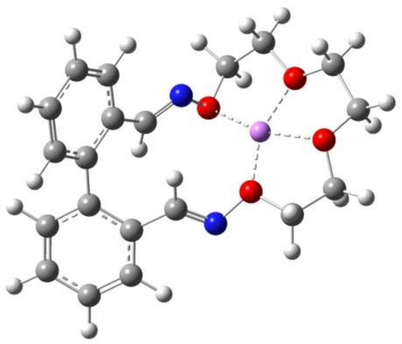

H	-2.90786700	-0.15651000	-5.09049200
C	-2.25192500	0.34463800	-4.38907800
C	-0.56062900	1.65179700	-2.57116100
C	-2.29414500	0.01846100	-3.04949700
C	-1.36638900	1.32042800	-4.83138000
C	-0.53597800	1.96333000	-3.93153400

C	-1.46085500	0.66281300	-2.13161200
H	-2.98329300	-0.73513400	-2.68834800
H	-1.32173200	1.57913200	-5.88271500
H	0.16333000	2.71278300	-4.28374800
C	0.34073800	2.40826000	-1.66772400
C	1.97673600	3.98331500	-0.01500800
C	1.24560100	1.80907700	-0.77099300
C	0.28702200	3.80189600	-1.71309400
C	1.09040000	4.58234600	-0.90094000
C	2.05015600	2.60669400	0.04687100
H	-0.41740900	4.27457000	-2.38763200
H	1.02377600	5.66238900	-0.95853200
H	2.75242400	2.12392000	0.71534100
H	2.61410900	4.59037000	0.61635400
C	-1.50591200	0.30723900	-0.72007800
H	-1.04987900	0.98417300	0.00046500
C	1.36523900	0.35668400	-0.69943400
H	1.09801600	-0.24433600	-1.56941800
N	-2.00751000	-0.79143400	-0.31406400
N	1.74871800	-0.21633100	0.37357800
O	-1.71002800	-0.95085500	1.05743200
O	1.70931400	-1.62132600	0.21565800
C	2.81591500	-2.22413200	0.88540600
H	2.75428600	-3.27494800	0.59811000
H	3.75586800	-1.80639900	0.51633700
C	2.71943900	-2.08424500	2.38675900
H	3.46836000	-2.72511700	2.86573800
H	2.88401600	-1.04867500	2.70490800
O	1.40692500	-2.49566500	2.72495100
C	1.06287000	-2.47303000	4.09937500
H	1.67684300	-3.17815800	4.66976200
H	1.20865700	-1.46565000	4.50961700
C	-0.38948400	-2.87180700	4.15360800

H	-0.50332000	-3.90320100	3.79772200
H	-0.77741900	-2.80598400	5.17532200
O	-1.07934700	-1.98367300	3.29019200
C	-2.31254300	-2.46423500	2.78077600
H	-2.14676000	-3.41902700	2.26358500
H	-3.04004300	-2.62726500	3.58373300
C	-2.82284400	-1.41654800	1.81834900
H	-3.25335100	-0.56218800	2.34781300
H	-3.57250900	-1.84003700	1.14757900
Li	0.07529600	-1.55360100	1.63159500

2-Li^{+}-rac

H (353 K, 1 atm) = -1191.668668 a.u.

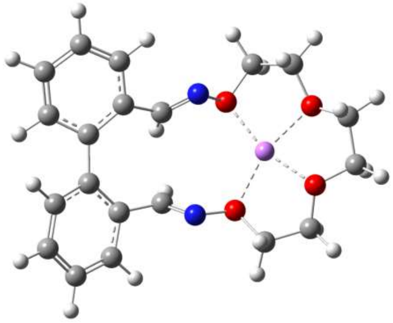

H	-3.90562900	-0.63999600	2.51147300
C	-2.97024500	-0.37002700	2.98571200
C	-0.46795800	0.27694400	4.21469100
C	-2.30647000	-1.27218300	3.79066900
C	-2.43519100	0.89654200	2.86082800
C	-1.23489400	1.19573600	3.47280700
C	-1.09312400	-0.97589000	4.42095500
H	-2.74209600	-2.24912700	3.96176600
H	-2.95291000	1.66680700	2.30182600
H	-0.89979800	2.21825000	3.39824200
C	0.84420400	0.81367000	4.71820900
C	2.99778800	2.38050000	5.74533500
C	1.62172100	0.37574600	5.81947600
C	1.33594600	1.96040000	4.07317900
C	2.37616000	2.72897600	4.55974500
C	2.63937700	1.18778100	6.33549800
H	0.89885800	2.26822400	3.13544300
H	2.69680600	3.60143800	4.00293100

H	3.19387600	0.81744900	7.18843300
H	3.79391300	2.98433900	6.16278200
C	-0.70651300	-2.09176600	5.32142000
H	0.03308900	-2.84262700	5.04204800
C	1.65502800	-0.99130800	6.32654200
H	1.60217300	-1.79777700	5.60100000
N	-1.43514900	-2.23428900	6.34855100
N	1.95438500	-1.27182100	7.53151900
O	-1.13353300	-3.39679000	7.08787500
O	2.13460200	-2.68008500	7.65166200
C	3.18705800	-2.95020000	8.58083300
H	3.53902700	-3.94997300	8.31839600
H	4.00049500	-2.23660200	8.44299100
C	2.66835700	-2.93492200	9.99617100
H	3.44173000	-3.28028600	10.69146100
H	2.35058200	-1.92673800	10.28881400
O	1.55776600	-3.81560400	9.98463200
C	0.79203300	-3.89793900	11.17288600
H	1.34258300	-4.41719400	11.96435300
H	0.53129700	-2.89169100	11.52427100
C	-0.44883300	-4.66358600	10.79237200
H	-0.18848700	-5.69095300	10.50899900
H	-1.16290000	-4.69442900	11.62161100
O	-0.99468000	-3.97720200	9.67769900
C	-1.95546600	-4.68207500	8.91375200
H	-1.52868300	-5.62693800	8.55116500
H	-2.84755000	-4.91234000	9.50681000
C	-2.33360000	-3.77203500	7.77949500
H	-2.80867800	-2.86265000	8.15308300
H	-3.01516600	-4.27682800	7.08981900
Li	0.40317800	-3.34871900	8.41328800

2-Li$^+$-c

H (353 K, 1 atm) = -1191.711135 a.u.

H	-4.69179500	2.23069700	-2.18453200
C	-3.60972800	2.18012100	-2.19113200
C	-0.80921400	2.03916300	-2.22477300
C	-2.96928400	1.03547800	-1.76240200
C	-2.85971000	3.25987300	-2.64065200
C	-1.47887200	3.18486000	-2.65679600
C	-1.57475500	0.94871200	-1.77545800
H	-3.54135600	0.17929900	-1.42699500
H	-3.35307800	4.16359000	-2.97844400
H	-0.89592000	4.03552800	-2.99043700
C	0.67039200	2.03296400	-2.27480300
C	3.46505200	2.06893700	-2.50419400
C	1.48260000	1.71845600	-1.17101600
C	1.29090400	2.36755700	-3.47927100
C	2.66865900	2.38522500	-3.59789300
C	2.87358800	1.73834800	-1.30208900
H	0.67210000	2.59654900	-4.33916700
H	3.12336800	2.64516100	-4.54652100
H	3.48175400	1.50782100	-0.43618500
H	4.54475300	2.08958500	-2.58957800
C	-0.92276200	-0.28276000	-1.33455100
H	0.07764500	-0.52015600	-1.69594300
C	0.88270800	1.38416100	0.11911200
H	-0.12135200	1.73792700	0.35256800
N	-1.51291800	-1.05324500	-0.51128400
N	1.52258300	0.66008200	0.94733800
O	-0.68421900	-2.13722500	-0.14294800
O	0.73979400	0.37777300	2.08979700

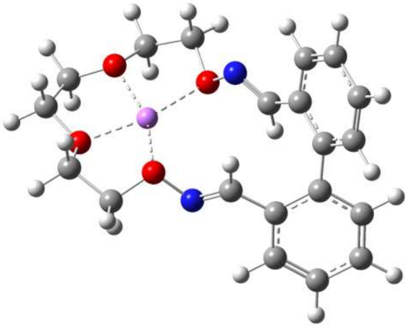

C	1.59069700	0.35649200	3.23869600
H	2.57962800	0.01011600	2.92844300
H	1.67695200	1.36309500	3.65719400
C	0.98482200	-0.59275300	4.23328000
H	1.63773800	-0.68073400	5.10873100
H	-0.00195700	-0.24706900	4.56798400
O	0.86177900	-1.83232000	3.55916800
C	0.23148800	-2.87329200	4.28440600
H	0.86414600	-3.21816900	5.10917300
H	-0.72302800	-2.51974600	4.69428900
C	-0.00072800	-3.97970500	3.28980900
H	0.95575000	-4.39366600	2.94646700
H	-0.59463700	-4.78481100	3.73535300
O	-0.68643400	-3.38111700	2.20428300
C	-0.82629800	-4.17924300	1.04259400
H	0.15961900	-4.51687100	0.69732100
H	-1.44636700	-5.06079200	1.23965000
C	-1.49513500	-3.30328100	0.02146900
H	-2.48236800	-2.98993300	0.36989900
H	-1.59931700	-3.82270500	-0.93518100
Li	0.05258300	-1.56977400	1.73554100

2-Na^{+}-a

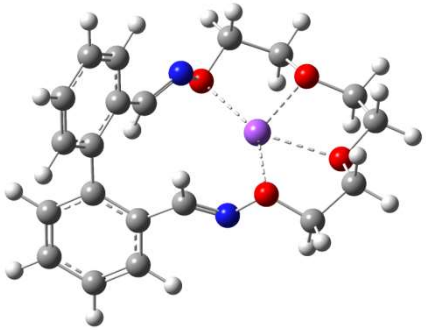

H (353 K, 1 atm) = -1346.398884 a.u.

H	-4.60125300	2.55179500	1.64363100
C	-3.53261100	2.42814200	1.77120000
C	-0.77590500	2.10728500	2.11425000
C	-2.94922100	1.19670600	1.54570500
C	-2.74942600	3.49898000	2.18393700
C	-1.38478900	3.33590500	2.35499200

C	-1.57408200	1.02068100	1.71707200
H	-3.55492700	0.34531600	1.26011000
H	-3.20384300	4.46438000	2.37290700
H	-0.76999100	4.17483000	2.66066000
C	0.69150200	1.97721500	2.26530900
C	3.44808200	1.66914800	2.62084900
C	1.51969300	1.67228100	1.17138600
C	1.27033600	2.14502800	3.52029700
C	2.63485700	1.99102100	3.70043500
C	2.89444700	1.51724100	1.36465100
H	0.63240000	2.37958400	4.36482200
H	3.06569800	2.12224200	4.68600900
H	3.52312700	1.30346700	0.50886400
H	4.51657500	1.55519700	2.75832100
C	-0.97132800	-0.29915800	1.54847600
H	-0.04617400	-0.52937600	2.07920800
C	0.94724700	1.58289100	-0.16950300
H	0.01509900	2.10858600	-0.38240800
N	-1.51763900	-1.17647800	0.79967900
N	1.52768400	0.90835400	-1.08435200
O	-0.76969800	-2.35750700	0.76390000
O	0.80439000	0.93845100	-2.28078000
C	1.64148000	0.57981800	-3.38374200
H	2.59354900	0.21512300	-2.99342400
H	1.82245700	1.46962200	-3.99286100
C	0.96254200	-0.48383800	-4.20314000
H	1.57328100	-0.70269400	-5.08797200
H	-0.02382500	-0.14171300	-4.54448100
O	0.82573700	-1.63407200	-3.38993400
C	0.14499600	-2.71369300	-4.00197100
H	0.70558100	-3.08593500	-4.86790000
H	-0.84642700	-2.38857600	-4.34446300
C	0.00714900	-3.81034800	-2.98013900

H	0.99894000	-4.15228200	-2.65553900
H	-0.52460300	-4.66113200	-3.42292900
O	-0.71135700	-3.28799800	-1.87813500
C	-0.85541400	-4.18045900	-0.78917000
H	0.13049000	-4.52412700	-0.44804100
H	-1.44167000	-5.06054000	-1.08170700
C	-1.57512900	-3.44946600	0.31144400
H	-2.52687500	-3.05377800	-0.04829800
H	-1.76296000	-4.12155400	1.15320000
Na	0.02824300	-1.16707700	-1.24826400

2-Na$^+$-b

H (353 K, 1 atm) = -1346.394968 a.u.

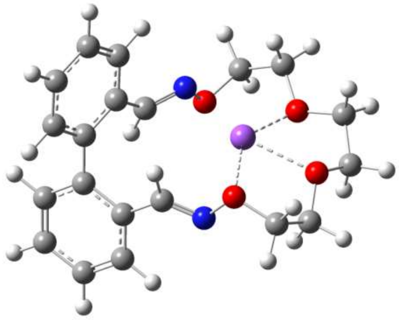

H	-2.44870600	-1.01653100	-4.77223400
C	-1.89752800	-0.32147400	-4.15045700
C	-0.47791900	1.48048800	-2.55385600
C	-2.10192000	-0.30641800	-2.78420100
C	-1.00111800	0.57038300	-4.72651700
C	-0.29922100	1.46394700	-3.93298700
C	-1.40321300	0.59231200	-1.97644700
H	-2.82437900	-0.97181800	-2.32704400
H	-0.84742900	0.56881200	-5.79917600
H	0.41275600	2.14801800	-4.38059300
C	0.31734200	2.40225600	-1.70681100
C	1.70294100	4.14333100	-0.01354200
C	1.28718900	1.90755500	-0.81666300
C	0.07935400	3.77174000	-1.74909400
C	0.76416300	4.63725000	-0.91015800
C	1.96845100	2.78731900	0.02693300
H	-0.66827000	4.15476900	-2.43436700

H	0.56378800	5.70117800	-0.95582600
H	2.73352900	2.39560500	0.68677600
H	2.24330200	4.82023600	0.63716300
C	-1.65897200	0.67741700	-0.54411400
H	-1.41804300	1.61397500	-0.03651100
C	1.62611600	0.48777500	-0.84467600
H	1.48966100	-0.06419100	-1.77581300
N	-2.15024900	-0.28918400	0.12965300
N	2.08751200	-0.12095600	0.17989600
O	-2.25093000	0.07922900	1.47777500
O	2.33550200	-1.45296600	-0.11994000
C	3.22818400	-2.05535700	0.80440100
H	3.33080200	-3.07119500	0.42085800
H	4.20606200	-1.56389900	0.77196200
C	2.72747200	-2.08972900	2.23250200
H	3.35704500	-2.78792400	2.79754500
H	2.80931700	-1.10525500	2.71167500
O	1.37912500	-2.51703300	2.21964600
C	0.87467100	-2.98029100	3.45839100
H	1.39227300	-3.89576100	3.76840700
H	1.01447300	-2.22307300	4.24239000
C	-0.59027800	-3.26246500	3.26011100
H	-0.71688500	-3.98920400	2.44750200
H	-1.02278300	-3.68261100	4.17559200
O	-1.22472200	-2.04088700	2.92497100
C	-2.52341100	-2.18478300	2.37808900
H	-2.47070400	-2.72013500	1.42169200
H	-3.17337100	-2.75349200	3.05478400
C	-3.10759200	-0.80519500	2.19408100
H	-3.23323400	-0.31489400	3.16068900
H	-4.07946900	-0.87235600	1.69861900
Na	0.06673400	-0.71574200	1.49988200

2-Na⁺-rac

H (353 K, 1 atm) = -1346.346631 a.u.

H	-3.95382200	-0.56749800	2.56726700
C	-3.00891700	-0.31995600	3.03477000
C	-0.47003900	0.26011500	4.23413000
C	-2.36049300	-1.23704100	3.83377700
C	-2.44069600	0.93048700	2.90287000
C	-1.22562200	1.19598800	3.50127400
C	-1.13035900	-0.97688600	4.45314100
H	-2.81459700	-2.20456400	4.00336400
H	-2.94079900	1.71513500	2.34780800
H	-0.87314000	2.21121200	3.42119200
C	0.85516900	0.77664400	4.73446200
C	2.99190600	2.34626000	5.80849500
C	1.66686000	0.29996700	5.79819500
C	1.32770900	1.95633700	4.13375300
C	2.35629500	2.72664100	4.64129000
C	2.66958900	1.11702400	6.34096200
H	0.88281200	2.29573400	3.21161400
H	2.65482300	3.62545200	4.11499800
H	3.25134300	0.71678500	7.16134900
H	3.77669400	2.94908300	6.24814900
C	-0.76192300	-2.09728200	5.34704800
H	0.09296100	-2.74766800	5.17009600
C	1.79498900	-1.09068600	6.22707400
H	1.82664600	-1.86053500	5.45790700
N	-1.61881400	-2.38494300	6.24469100
N	2.10518800	-1.40591600	7.42596300
O	-1.29020300	-3.53691700	6.96371500

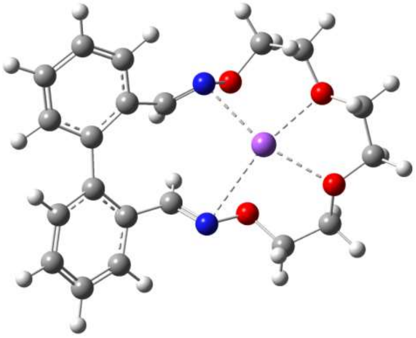

O	2.40746500	-2.77649900	7.50780900
C	3.31011300	-3.03366300	8.58138500
H	3.61583000	-4.06754200	8.41491600
H	4.18626000	-2.38474800	8.50333900
C	2.67800900	-2.90067400	9.94985200
H	3.38747900	-3.25908000	10.70605900
H	2.44077600	-1.85243800	10.18006000
O	1.50429100	-3.68585900	9.92965100
C	0.74798400	-3.74803700	11.12150500
H	1.30925600	-4.24094100	11.92413800
H	0.48001900	-2.73829400	11.46635100
C	-0.48733700	-4.54940700	10.79391000
H	-0.18564800	-5.55205100	10.46977000
H	-1.12844600	-4.64460200	11.67706000
O	-1.17781500	-3.89253900	9.74165300
C	-1.94689800	-4.74663800	8.91204800
H	-1.33483900	-5.59631700	8.58323600
H	-2.81743800	-5.13894300	9.45138700
C	-2.42867800	-3.95803600	7.72552300
H	-2.98779500	-3.07361100	8.04091900
H	-3.07683200	-4.58077600	7.10247600
Na	0.16516500	-2.52206900	8.43112100

2-Na⁺-c

H (353 K, 1 atm) = -1346.398884 a.u.

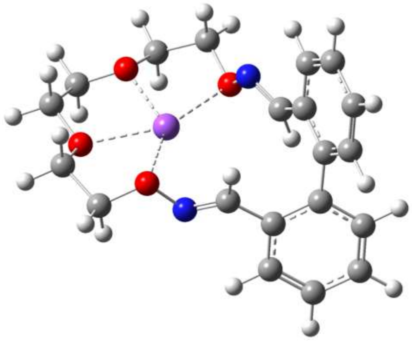

H	-4.60125300	2.55179500	-1.64363100
C	-3.53261100	2.42814200	-1.77120000
C	-0.77590500	2.10728500	-2.11425000
C	-2.94922100	1.19670600	-1.54570500
C	-2.74942600	3.49898000	-2.18393700
C	-1.38478900	3.33590500	-2.35499200

C	-1.57408200	1.02068100	-1.71707200
H	-3.55492700	0.34531600	-1.26011000
H	-3.20384300	4.46438000	-2.37290700
H	-0.76999100	4.17483000	-2.66066000
C	0.69150200	1.97721500	-2.26530900
C	3.44808200	1.66914800	-2.62084900
C	1.51969300	1.67228100	-1.17138600
C	1.27033600	2.14502800	-3.52029700
C	2.63485700	1.99102100	-3.70043500
C	2.89444700	1.51724100	-1.36465100
H	0.63240000	2.37958400	-4.36482200
H	3.06569800	2.12224200	-4.68600900
H	3.52312700	1.30346700	-0.50886400
H	4.51657500	1.55519700	-2.75832100
C	-0.97132800	-0.29915800	-1.54847600
H	-0.04617400	-0.52937600	-2.07920800
C	0.94724700	1.58289100	0.16950300
H	0.01509900	2.10858600	0.38240800
N	-1.51763900	-1.17647800	-0.79967900
N	1.52768400	0.90835400	1.08435200
O	-0.76969800	-2.35750700	-0.76390000
O	0.80439000	0.93845100	2.28078000
C	1.64148000	0.57981800	3.38374200
H	2.59354900	0.21512300	2.99342400
H	1.82245700	1.46962200	3.99286100
C	0.96254200	-0.48383800	4.20314000
H	1.57328100	-0.70269400	5.08797200
H	-0.02382500	-0.14171300	4.54448100
O	0.82573700	-1.63407200	3.38993400
C	0.14499600	-2.71369300	4.00197100
H	0.70558100	-3.08593500	4.86790000
H	-0.84642700	-2.38857600	4.34446300
C	0.00714900	-3.81034800	2.98013900

H	0.99894000	-4.15228200	2.65553900
H	-0.52460300	-4.66113200	3.42292900
O	-0.71135700	-3.28799800	1.87813500
C	-0.85541400	-4.18045900	0.78917000
H	0.13049000	-4.52412700	0.44804100
H	-1.44167000	-5.06054000	1.08170700
C	-1.57512900	-3.44946600	-0.31144400
H	-2.52687500	-3.05377800	0.04829800
H	-1.76296000	-4.12155400	-1.15320000
Na	0.02824300	-1.16707700	1.24826400

2-K$^+$-a

H (353 K, 1 atm) = -1783.971162 a.u.

H	1.16067000	3.16447000	-3.56244200
C	1.09968100	3.19223300	-2.48113300
C	0.93556900	3.26963600	0.30577500
C	0.57336500	2.11647100	-1.79274600
C	1.53042700	4.31604700	-1.78669700
C	1.44539100	4.35237800	-0.40467100
C	0.48232400	2.14252100	-0.40000600
H	0.20392700	1.24846700	-2.32515800
H	1.93387400	5.16627300	-2.32381700
H	1.79440900	5.22222800	0.14024500
C	0.88967200	3.31619000	1.78674800
C	0.70038400	3.38050600	4.57675800
C	1.68063900	2.45779800	2.56983000
C	0.03171500	4.20909400	2.42292200
C	-0.06922500	4.24114400	3.80422900
C	1.57550000	2.50372100	3.96319900
H	-0.57622000	4.87165000	1.81733700
H	-0.74529800	4.94213500	4.27926200

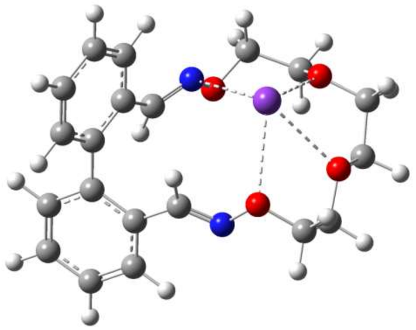

H	2.22606800	1.87322600	4.55849500
H	0.63776000	3.41546600	5.65792700
C	2.65264100	1.57405400	1.92759600
H	3.03576200	1.83203400	0.94066000
C	-0.12883900	1.04027700	0.33510000
H	-0.50966800	1.24557100	1.33762200
N	-0.24000300	-0.12517100	-0.16195200
N	3.06066600	0.51846300	2.51628800
O	3.99415600	-0.16082200	1.76578700
O	-0.86203100	-0.99145500	0.73143100
C	4.71487500	-1.10577800	2.54776500
H	5.77858100	-0.89849700	2.40116300
H	4.47771100	-0.96308200	3.60446000
C	4.42600600	-2.51622100	2.11550100
H	4.49915500	-2.58073500	1.02275200
H	5.18127000	-3.18504500	2.54928000
C	-1.13846100	-2.22929900	0.08489600
H	-1.71582500	-2.05168800	-0.82622400
H	-1.76103500	-2.77718500	0.79535400
C	0.10561900	-3.00959200	-0.24428600
H	-0.18554800	-3.94598900	-0.73892500
H	0.73724500	-2.43624700	-0.93351400
O	0.81137400	-3.27913600	0.95218400
O	3.13643800	-2.90256600	2.55691400
C	2.71751100	-4.15921000	2.04915900
H	3.57182200	-4.83584500	1.93504400
H	2.04741000	-4.59669000	2.79458200
C	1.99078200	-4.02576800	0.73540600
H	2.62246400	-3.52223900	-0.00933600
H	1.74807500	-5.02510300	0.34924300
K	0.93649700	-1.30554500	2.91674300

2-K$^+$-b

H (353 K, 1 atm) = -1783.963867 a.u.

H	-2.38043000	-1.04782100	-4.95358700
C	-1.84259600	-0.36788900	-4.30392700
C	-0.46060300	1.39280200	-2.63413200
C	-2.12661900	-0.33659700	-2.95219400
C	-0.88179300	0.48634400	-4.83027800
C	-0.19805900	1.35985300	-3.99941900
C	-1.44552000	0.54070600	-2.10703100
H	-2.89417700	-0.97683000	-2.53448700
H	-0.66461500	0.47263300	-5.89178600
H	0.56096100	2.01798200	-4.40697700
C	0.31414400	2.30167200	-1.75017600
C	1.68223700	4.02873600	-0.02653900
C	1.32873000	1.80733500	-0.91153800
C	0.01837800	3.66109300	-1.72161500
C	0.69041400	4.51995600	-0.86596100
C	2.00542400	2.68445100	-0.05763700
H	-0.76119400	4.04126800	-2.37207500
H	0.44495600	5.57534200	-0.85945500
H	2.81924500	2.30145500	0.54789600
H	2.22303900	4.70076700	0.62923800
C	-1.76613200	0.62480100	-0.68707200
H	-1.50573500	1.54881000	-0.16454200
C	1.69479000	0.39319200	-0.97411400
H	1.47635400	-0.17000500	-1.88137600
N	-2.34042300	-0.31982900	-0.05453300
N	2.25455700	-0.18892800	0.01470900
O	-2.51610200	0.02229600	1.28175700
O	2.50927500	-1.50679300	-0.26802300

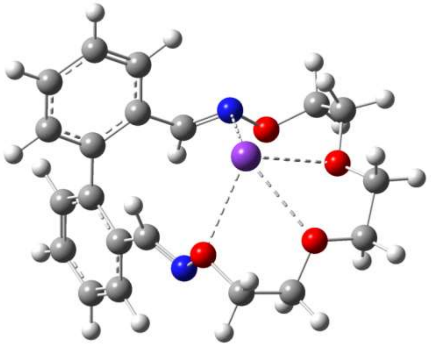

C	3.31846400	-2.12545700	0.71524100
H	3.39591100	-3.15374400	0.36015100
H	4.31967800	-1.68044300	0.72920700
C	2.74365000	-2.10657400	2.11647700
H	3.30735900	-2.82906700	2.72035600
H	2.87982000	-1.12080200	2.58577500
O	1.37592800	-2.44262500	2.05737600
C	0.83290600	-2.99478500	3.23706900
H	1.35628700	-3.91969900	3.51069700
H	0.92771200	-2.29746500	4.08451800
C	-0.61441800	-3.30289600	2.96887300
H	-0.67974500	-3.94224800	2.07977700
H	-1.04556500	-3.84545000	3.81950900
O	-1.31647700	-2.09350000	2.74806100
C	-2.55188900	-2.29243700	2.08504900
H	-2.37558400	-2.72406700	1.09254900
H	-3.19046100	-2.98210500	2.65313400
C	-3.27280300	-0.97598900	1.95941000
H	-3.46316700	-0.54367900	2.94360800
H	-4.22773600	-1.12911300	1.44944900
K	0.17833800	0.08461300	1.98141400

2-K^{+}-rac

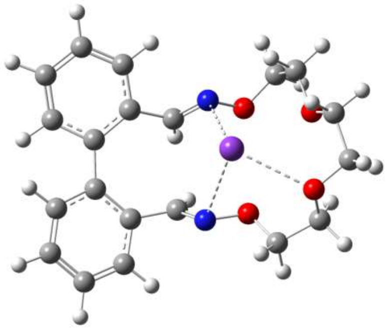

H (353 K, 1 atm) = -1783.915733 a.u.

H	-4.04986600	-0.47063700	2.77982600
C	-3.08034300	-0.24053900	3.20393400
C	-0.48661000	0.30365000	4.29537800
C	-2.38873300	-1.18771800	3.92848500
C	-2.51674900	1.01401000	3.08338800
C	-1.27414300	1.26312900	3.62987600

C	-1.12703100	-0.94578800	4.48647400
H	-2.83022200	-2.16405000	4.08402000
H	-3.04323900	1.81464300	2.57767900
H	-0.92528200	2.28035300	3.56038200
C	0.85535100	0.79187300	4.77937100
C	3.06277500	2.25262700	5.86062600
C	1.70678300	0.22784900	5.76514400
C	1.30243000	2.01841400	4.25753200
C	2.36499600	2.73702100	4.76988700
C	2.75034900	0.98827800	6.31219300
H	0.81188500	2.43983300	3.39447700
H	2.64243700	3.67639800	4.30670000
H	3.36760800	0.51868000	7.06763300
H	3.88149900	2.80867300	6.30038100
C	-0.68515100	-2.11165700	5.28422100
H	0.10330300	-2.78746100	4.96041100
C	1.80398600	-1.19083100	6.12355100
H	1.85082300	-1.92411400	5.32136200
N	-1.40537100	-2.40802900	6.29127600
N	2.06150100	-1.55938400	7.31690900
O	-0.98668500	-3.56702100	6.93129200
O	2.31626400	-2.91662600	7.37884800
C	3.13948100	-3.23643700	8.48952400
H	3.29150200	-4.31229600	8.39272400
H	4.10907200	-2.73388100	8.40416100
C	2.53127300	-2.92649600	9.83583200
H	3.20614000	-3.30739300	10.61472400
H	2.43972000	-1.84017200	9.98536900
O	1.27181700	-3.54885400	9.89971000
C	0.60034100	-3.42764900	11.12615500
H	1.19039900	-3.83574500	11.95798300
H	0.41046500	-2.36609300	11.36743100
C	-0.69781900	-4.19137100	10.99891500

H	-0.47864000	-5.26045700	10.92700700
H	-1.31563500	-4.03188200	11.88804600
O	-1.40469100	-3.75929900	9.84651000
C	-1.61588200	-4.78653100	8.88932300
H	-0.68364600	-5.33622100	8.71617700
H	-2.37567800	-5.48994000	9.25197400
C	-2.08773800	-4.18827800	7.59579200
H	-2.87359200	-3.44678900	7.76701600
H	-2.49367700	-4.97506700	6.95190700
K	-0.40065700	-1.41185500	8.72670300

2-K^{+}-c

H (353 K, 1 atm) = -1783.965614 a.u.

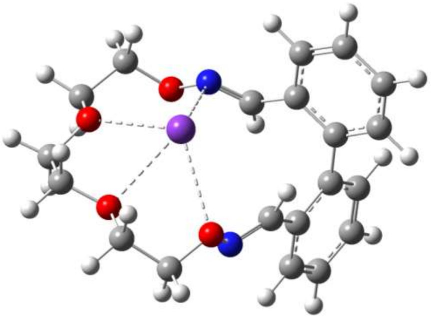

H	-4.39613900	2.61500200	-1.30646800
C	-3.34602800	2.44774200	-1.51483500
C	-0.64569200	2.00976300	-2.09311900
C	-2.83267100	1.16354700	-1.48936900
C	-2.52112400	3.51448900	-1.84660500
C	-1.18391300	3.29195600	-2.13652400
C	-1.48364800	0.92855200	-1.77347100
H	-3.48145100	0.31767800	-1.29241500
H	-2.92134600	4.52065700	-1.88684600
H	-0.53538200	4.12380900	-2.38715500
C	0.80268500	1.80147400	-2.34218200
C	3.50680000	1.32410700	-2.83840400
C	1.68551900	1.56542800	-1.27631100
C	1.29554400	1.81282700	-3.64281600
C	2.63759200	1.57478200	-3.89255000
C	3.03536200	1.32599000	-1.53964700
H	0.61236900	1.99783500	-4.46409800

H	3.00617900	1.58651700	-4.91144900
H	3.70968000	1.16053100	-0.70828700
H	4.55742200	1.14293400	-3.03103700
C	-0.95578400	-0.43751700	-1.78478100
H	-0.11416000	-0.68415100	-2.43146800
C	1.18551800	1.61935500	0.09582300
H	0.26956200	2.18253000	0.28955200
N	-1.48657600	-1.32864400	-1.04178000
N	1.80420700	1.04399500	1.04842200
O	-0.91318500	-2.56258600	-1.20097400
O	1.16023800	1.21859000	2.26458900
C	1.96014400	0.70110600	3.32796800
H	2.86004700	0.26380400	2.89481500
H	2.23977300	1.52760500	3.98735400
C	1.19336600	-0.33979100	4.10114700
H	1.82891900	-0.71368400	4.91482400
H	0.29141700	0.09248000	4.56186000
O	0.85026600	-1.37600100	3.20794000
C	0.29705300	-2.52744100	3.80510000
H	0.94773000	-2.90438900	4.60465000
H	-0.68355900	-2.30460200	4.25387300
C	0.17074600	-3.58810400	2.74681600
H	1.16248400	-3.79765700	2.32602300
H	-0.21186100	-4.51000400	3.20451200
O	-0.70001800	-3.14873800	1.72052700
C	-0.79036200	-4.09855300	0.67351000
H	0.21505900	-4.35174800	0.31275700
H	-1.26051800	-5.02051200	1.04288200
C	-1.60992900	-3.56444400	-0.46924400
H	-2.56525400	-3.16312400	-0.12300800
H	-1.81218200	-4.38278800	-1.16559300
K	-1.11706000	-0.41651500	1.54542000

2-K^{+}-d

H (353 K, 1 atm) = -1783.971162 a.u.

H	1.16067000	3.16447000	3.56244200
C	1.09968100	3.19223300	2.48113300
C	0.93556900	3.26963600	-0.30577500
C	0.57336500	2.11647100	1.79274600
C	1.53042700	4.31604700	1.78669700
C	1.44539100	4.35237800	0.40467100
C	0.48232400	2.14252100	0.40000600
H	0.20392700	1.24846700	2.32515800
H	1.93387400	5.16627300	2.32381700
H	1.79440900	5.22222800	-0.14024500
C	0.88967200	3.31619000	-1.78674800
C	0.70038400	3.38050600	-4.57675800
C	1.68063900	2.45779800	-2.56983000
C	0.03171500	4.20909400	-2.42292200
C	-0.06922500	4.24114400	-3.80422900
C	1.57550000	2.50372100	-3.96319900
H	-0.57622000	4.87165000	-1.81733700
H	-0.74529800	4.94213500	-4.27926200
H	2.22606800	1.87322600	-4.55849500
H	0.63776000	3.41546600	-5.65792700
C	2.65264100	1.57405400	-1.92759600
H	3.03576200	1.83203400	-0.94066000
C	-0.12883900	1.04027700	-0.33510000
H	-0.50966800	1.24557100	-1.33762200
N	-0.24000300	-0.12517100	0.16195200
N	3.06066600	0.51846300	-2.51628800
O	3.99415600	-0.16082200	-1.76578700

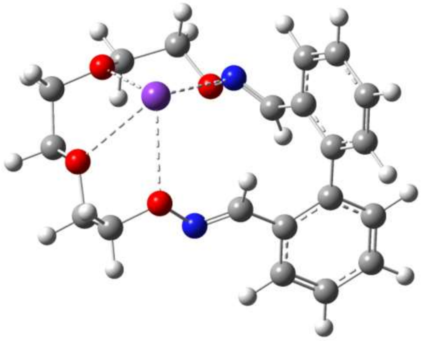

O	-0.86203100	-0.99145500	-0.73143100
C	4.71487500	-1.10577800	-2.54776500
H	5.77858100	-0.89849700	-2.40116300
H	4.47771100	-0.96308200	-3.60446000
C	4.42600600	-2.51622100	-2.11550100
H	4.49915500	-2.58073500	-1.02275200
H	5.18127000	-3.18504500	-2.54928000
C	-1.13846100	-2.22929900	-0.08489600
H	-1.71582500	-2.05168800	0.82622400
H	-1.76103500	-2.77718500	-0.79535400
C	0.10561900	-3.00959200	0.24428600
H	-0.18554800	-3.94598900	0.73892500
H	0.73724500	-2.43624700	0.93351400
O	0.81137400	-3.27913600	-0.95218400
O	3.13643800	-2.90256600	-2.55691400
C	2.71751100	-4.15921000	-2.04915900
H	3.57182200	-4.83584500	-1.93504400
H	2.04741000	-4.59669000	-2.79458200
C	1.99078200	-4.02576800	-0.73540600
H	2.62246400	-3.52223900	0.00933600
H	1.74807500	-5.02510300	-0.34924300
K	0.93649700	-1.30554500	-2.91674300

3-Li$^+$-a

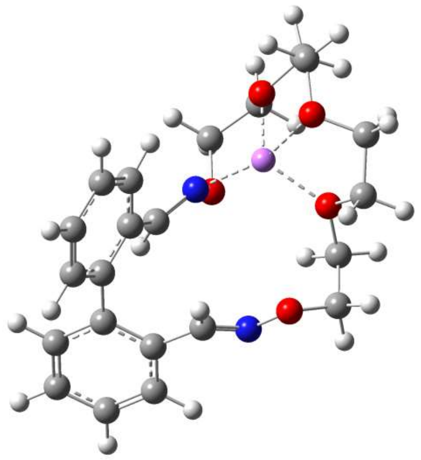

H (353 K, 1 atm) = -1345.380076 a.u.

H	-0.78368400	5.62841000	-2.85148300
C	-0.59841100	5.21694800	-1.86644000
C	-0.12445900	4.15942800	0.67026900
C	-0.83436900	3.87573500	-1.62465300
C	-0.14822100	6.03928300	-0.84151000
C	0.08518500	5.51210300	0.41814100

C	-0.60823500	3.33562500	-0.35949300
H	-1.22203600	3.23228200	-2.40506100
H	0.02458300	7.09309900	-1.02504800
H	0.46020100	6.14609800	1.21390100
C	0.19343900	3.59023400	1.99709500
C	0.70669100	2.45023900	4.50257500
C	1.14025300	2.55864100	2.13146600
C	-0.46476900	4.04585800	3.13551100
C	-0.21602400	3.48141400	4.37582400
C	1.38581900	1.99910300	3.38745400
H	-1.19841600	4.83741100	3.03445900
H	-0.74360500	3.84747900	5.24874500
H	2.15337300	1.23994500	3.48692500
H	0.91030200	2.01759500	5.47463500
C	1.93375800	2.15652700	0.97554100
H	2.09523300	2.87525300	0.17285600
C	-0.92575600	1.93889000	-0.06591400
H	-1.27899000	1.68053400	0.93309900
N	-0.79441700	1.03184800	-0.94541000
N	2.46722500	1.00376600	0.88738900
O	3.23038500	0.88402300	-0.29250600
O	-1.21400900	-0.19085100	-0.45843900
C	4.56873400	0.51594300	0.01779900
H	5.15005400	0.80489800	-0.86053600
H	4.92335700	1.09674700	0.87474900
C	4.76725200	-0.95903900	0.26096600
H	4.43117500	-1.54181800	-0.60705700
H	5.83721300	-1.14935000	0.41282500
C	-0.86915500	-1.20760800	-1.37150300
H	-1.12230700	-0.91024100	-2.39410300
H	-1.49637300	-2.05946500	-1.10005500
C	0.60131500	-1.55492400	-1.32028100
H	0.80336200	-2.37404500	-2.02232200

H	1.19946000	-0.68902900	-1.60817800
O	1.06440400	-1.91982000	-0.02224000
O	4.03248600	-1.34678300	1.41011900
C	1.32445400	-3.64731400	1.55602200
H	2.10613200	-4.14968700	0.97651800
C	0.36174200	-2.97794700	0.61209600
H	0.01294900	-3.71391800	-0.12069300
H	-0.50829800	-2.58092500	1.14667600
H	0.80826900	-4.39468800	2.16797400
C	4.24026600	-2.68313800	1.82495600
H	5.24532600	-2.81039500	2.24437600
H	4.14324000	-3.36103500	0.96685800
C	3.19362700	-2.96770700	2.87857100
H	3.35490100	-2.32718800	3.74771400
H	3.24381500	-4.01063700	3.20671000
O	1.89719900	-2.64556700	2.38849100
Li	2.09539900	-0.92977200	1.31787000

3-Li^{+}-rac

H (353 K, 1 atm) = -1345.330837 a.u.

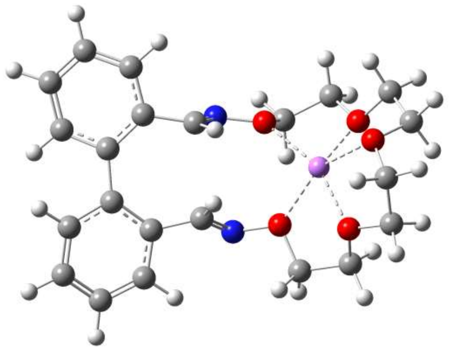

H	-3.02993900	-2.27746500	2.11457800
C	-2.26738800	-1.76480100	2.68787100
C	-0.22005200	-0.46932300	4.19113100
C	-1.75735400	-2.32901200	3.83999100
C	-1.82347400	-0.50530700	2.32846500
C	-0.83661600	0.11462000	3.06902800
C	-0.78007000	-1.69376600	4.60533200
H	-2.14583000	-3.27961200	4.18882800
H	-2.24957200	0.00826400	1.47485700
H	-0.55596200	1.11682400	2.77942100
C	0.92701700	0.30850400	4.77122100

C	2.80749000	2.29193000	5.58073900
C	1.40273800	0.37022200	6.10647500
C	1.57364300	1.16868600	3.86903900
C	2.48822300	2.13411900	4.24536200
C	2.28851700	1.38739200	6.48400200
H	1.35847500	1.07278900	2.81460400
H	2.94422100	2.76051000	3.48786900
H	2.60465300	1.41740400	7.51887000
H	3.49337100	3.06302900	5.90923600
C	-0.53491100	-2.37006000	5.89665400
H	0.16812900	-3.20229700	5.98695400
C	1.28458000	-0.67013500	7.13549600
H	1.49912300	-1.69578000	6.84239200
N	-1.26909200	-2.00171100	6.85964200
N	1.15774200	-0.38896300	8.36825100
O	-1.05081500	-2.71945500	8.02461300
O	1.35866200	-1.55647500	9.16622700
C	2.56273300	-1.36658300	9.89387600
H	3.40215800	-1.31556700	9.19103100
H	2.51954000	-0.43336800	10.46351000
C	2.72036000	-2.54964500	10.80764300
H	3.73269700	-2.55646300	11.22622100
H	2.00101300	-2.51712100	11.63671300
O	2.48542400	-3.70281800	10.01803700
C	0.01404200	-6.05138500	10.71396900
H	-0.10839100	-7.13733100	10.79787900
H	0.46595200	-5.68204100	11.64169700
C	-1.31783800	-5.39538500	10.50788300
H	-1.80386000	-5.80151900	9.61231900
H	-1.96591100	-5.57859400	11.37249700
O	-1.08687400	-4.00729400	10.33237500
C	-2.25573400	-3.28643300	9.95701500
H	-2.92420200	-3.94621300	9.39368300

H	-2.78380100	-2.92301000	10.84480800
C	-1.82494600	-2.14599300	9.08412900
H	-1.20743300	-1.42310300	9.62593300
H	-2.68677600	-1.62933400	8.66159100
C	2.84044100	-4.92876700	10.62486500
H	3.92938500	-5.05329300	10.64447700
H	2.47981000	-4.95419900	11.66082600
C	2.19924800	-6.00706700	9.78294500
H	2.34721700	-6.99398800	10.23436000
H	2.64460500	-6.01562400	8.78635300
O	0.82170900	-5.72768000	9.59408300
Li	0.57631700	-3.55842300	9.21412100

3-Li^{+}-b

H (353 K, 1 atm) = -1345.380076 a.u.

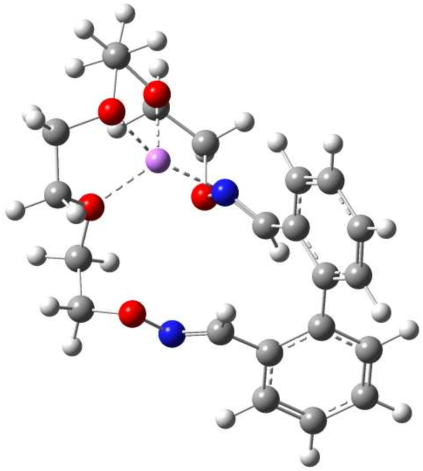

H	-0.78368400	5.62841000	2.85148300
C	-0.59841100	5.21694800	1.86644000
C	-0.12445900	4.15942800	-0.67026900
C	-0.83436900	3.87573500	1.62465300
C	-0.14822100	6.03928300	0.84151000
C	0.08518500	5.51210300	-0.41814100
C	-0.60823500	3.33562500	0.35949300
H	-1.22203600	3.23228200	2.40506100
H	0.02458300	7.09309900	1.02504800
H	0.46020100	6.14609800	-1.21390100
C	0.19343900	3.59023400	-1.99709500
C	0.70669100	2.45023900	-4.50257500
C	1.14025300	2.55864100	-2.13146600
C	-0.46476900	4.04585800	-3.13551100
C	-0.21602400	3.48141400	-4.37582400
C	1.38581900	1.99910300	-3.38745400

H	-1.19841600	4.83741100	-3.03445900
H	-0.74360500	3.84747900	-5.24874500
H	2.15337300	1.23994500	-3.48692500
H	0.91030200	2.01759500	-5.47463500
C	1.93375800	2.15652700	-0.97554100
H	2.09523300	2.87525300	-0.17285600
C	-0.92575600	1.93889000	0.06591400
H	-1.27899000	1.68053400	-0.93309900
N	-0.79441700	1.03184800	0.94541000
N	2.46722500	1.00376600	-0.88738900
O	3.23038500	0.88402300	0.29250600
O	-1.21400900	-0.19085100	0.45843900
C	4.56873400	0.51594300	-0.01779900
H	5.15005400	0.80489800	0.86053600
H	4.92335700	1.09674700	-0.87474900
C	4.76725200	-0.95903900	-0.26096600
H	4.43117500	-1.54181800	0.60705700
H	5.83721300	-1.14935000	-0.41282500
C	-0.86915500	-1.20760800	1.37150300
H	-1.12230700	-0.91024100	2.39410300
H	-1.49637300	-2.05946500	1.10005500
C	0.60131500	-1.55492400	1.32028100
H	0.80336200	-2.37404500	2.02232200
H	1.19946000	-0.68902900	1.60817800
O	1.06440400	-1.91982000	0.02224000
O	4.03248600	-1.34678300	-1.41011900
C	1.32445400	-3.64731400	-1.55602200
H	2.10613200	-4.14968700	-0.97651800
C	0.36174200	-2.97794700	-0.61209600
H	0.01294900	-3.71391800	0.12069300
H	-0.50829800	-2.58092500	-1.14667600
H	0.80826900	-4.39468800	-2.16797400
C	4.24026600	-2.68313800	-1.82495600

H	5.24532600	-2.81039500	-2.24437600
H	4.14324000	-3.36103500	-0.96685800
C	3.19362700	-2.96770700	-2.87857100
H	3.35490100	-2.32718800	-3.74771400
H	3.24381500	-4.01063700	-3.20671000
O	1.89719900	-2.64556700	-2.38849100
Li	2.09539900	-0.92977200	-1.31787000

3-Na^{+}-a

H (353 K, 1 atm) = -1500.066985 a.u.

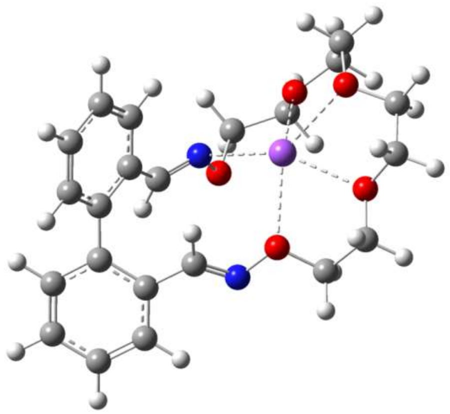

H	-4.47161000	3.62289300	2.98050100
C	-3.45812700	3.24153300	2.94665300
C	-0.84344700	2.25874800	2.86313100
C	-3.18754600	2.06176500	2.28055400
C	-2.43256900	3.92747200	3.58624400
C	-1.13863500	3.43745500	3.54456500
C	-1.88800500	1.55648800	2.23680100
H	-3.98216000	1.50259200	1.80174500
H	-2.64217400	4.84957700	4.11532500
H	-0.33413300	3.98433500	4.02327000
C	0.55280000	1.77664800	2.79828700
C	3.16209300	0.76134200	2.72831300
C	1.18220600	1.50946300	1.56949500
C	1.26247000	1.54753100	3.97476800
C	2.55227600	1.04439500	3.94407700
C	2.48156000	0.99882000	1.54914200
H	0.77889300	1.74507500	4.92466400
H	3.08381300	0.87039100	4.87223300
H	2.96220300	0.83013200	0.59249200
H	4.17462800	0.37680400	2.70257500
C	-1.59966800	0.27990800	1.58985700

H	-0.73351500	-0.28698200	1.93312000
C	0.50677300	1.84086600	0.31644300
H	-0.20214000	2.66866500	0.29836900
N	-2.31253000	-0.16918700	0.63915400
N	0.74100400	1.19163800	-0.75385000
O	-1.79364800	-1.37854800	0.17862200
O	-0.00683800	1.71222200	-1.82263300
C	0.81551700	1.89432000	-2.96814600
H	1.82016800	2.19203800	-2.65636800
H	0.36285700	2.72460500	-3.51591100
C	0.88283700	0.69806200	-3.88069500
H	1.46342900	0.98170200	-4.76864400
H	-0.12272800	0.40640500	-4.21391200
O	1.50611700	-0.38158800	-3.21122900
C	0.85049600	-4.02445700	-2.90751400
H	1.44329700	-4.92773600	-3.09777000
H	0.43135900	-3.67952100	-3.86046300
C	-0.27064300	-4.34364900	-1.96309900
H	0.12357500	-4.71731500	-1.00845800
H	-0.90113800	-5.12604300	-2.40413400
O	-1.01579100	-3.16133400	-1.74931600
C	-2.18463800	-3.37587400	-0.98054400
H	-1.93674500	-3.93515000	-0.06900200
H	-2.91936700	-3.95549500	-1.55304300
C	-2.76676400	-2.04286100	-0.61584500
H	-2.99429200	-1.44669500	-1.50609100
H	-3.68734000	-2.18361100	-0.04292600
C	1.99594600	-1.38398100	-4.07655900
H	2.76298700	-0.97345300	-4.74634000
H	1.18409600	-1.77919300	-4.70256700
C	2.60685700	-2.46421400	-3.21827800
H	3.05191800	-3.24208600	-3.84925500
H	3.39772400	-2.03350800	-2.60033600

O	1.65925200	-3.02074500	-2.32249600
Na	0.36895000	-1.20466400	-1.23415100

3-Na⁺-b

H (353 K, 1 atm) = -1500.065749 a.u.

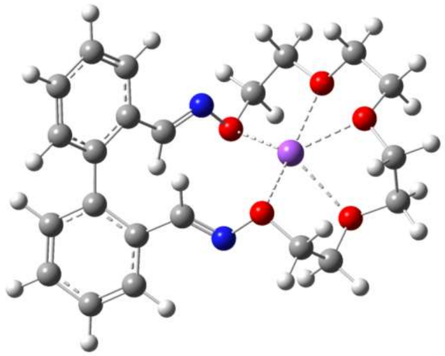

H	-2.71396700	-1.17635900	-4.79426900
C	-2.11881200	-0.41050900	-4.31167800
C	-0.59083500	1.57932700	-3.06637600
C	-2.18617600	-0.24284800	-2.94363300
C	-1.29712400	0.41743900	-5.06754600
C	-0.54621200	1.40084200	-4.44846700
C	-1.43222000	0.74700700	-2.30741600
H	-2.84303900	-0.86283100	-2.34588400
H	-1.24180200	0.29509300	-6.14284100
H	0.10555700	2.03668300	-5.03660100
C	0.23947900	2.64510100	-2.45959500
C	1.73879400	4.74074100	-1.36620700
C	1.20210200	2.38870400	-1.46772000
C	0.06137500	3.95740600	-2.89582100
C	0.79792100	4.99685400	-2.35631600
C	1.94015900	3.44669100	-0.93072800
H	-0.68414000	4.15866600	-3.65661800
H	0.63759900	6.00940100	-2.70737600
H	2.68948000	3.23045200	-0.17927600
H	2.32220400	5.55070500	-0.94521500
C	-1.54576700	0.94797600	-0.86681700
H	-1.20971300	1.89202100	-0.43760700
C	1.46167500	1.01812800	-1.03509900
H	1.13407900	0.19405400	-1.67001300
N	-2.02197100	0.04282600	-0.10688700
N	2.06677900	0.77861200	0.05855400

O	-1.96703200	0.45189600	1.23668800
O	2.19977200	-0.60069800	0.26647000
C	3.30247000	-0.82767500	1.13983900
H	3.52379400	-1.89085200	1.03629000
H	4.17204800	-0.26248100	0.79268000
C	2.98161600	-0.45483600	2.56936400
H	3.85341400	-0.64231700	3.20647500
H	2.74801400	0.61030000	2.61947300
O	1.84017000	-1.13433400	3.06858300
C	-0.61355800	-3.92200700	2.98699600
H	-0.55607700	-5.01688700	3.01681000
H	-0.52755800	-3.54158500	4.01220900
C	-1.93304600	-3.49779300	2.40906900
H	-2.03852300	-3.87159600	1.38167100
H	-2.74699700	-3.91685800	3.01388600
O	-1.97790100	-2.08527600	2.42046400
C	-3.19322400	-1.55473800	1.93022700
H	-3.37931000	-1.91671800	0.91312700
H	-4.03338000	-1.85974900	2.56767500
C	-3.09222000	-0.05141300	1.95082500
H	-2.94195000	0.30940900	2.96994000
H	-4.01391000	0.38482400	1.55341900
C	2.05472600	-2.40776500	3.65569800
H	3.09006500	-2.51036400	3.99966000
H	1.41161600	-2.45083600	4.53901100
C	1.71808400	-3.54166600	2.70542600
H	1.82306200	-4.50186300	3.22566200
H	2.39753000	-3.55397600	1.85174800
O	0.41585800	-3.39827500	2.16696400
Na	0.00429900	-1.07807700	1.54151100

3-Na$^+$-rac

H (353 K, 1 atm) = -1500.019697 a.u.

H	-3.16764800	-2.02315400	2.10939300
C	-2.38490600	-1.51392100	2.65787000
C	-0.27433900	-0.23002300	4.10187400
C	-1.84577800	-2.07355300	3.79713500
C	-1.94015200	-0.25929800	2.28541800
C	-0.92879800	0.35160400	2.99788300
C	-0.83903000	-1.44776500	4.53651800
H	-2.23333800	-3.01911700	4.16041400
H	-2.38497000	0.25963600	1.44471300
H	-0.66423600	1.35197900	2.69374600
C	0.88754400	0.54720900	4.65832100
C	2.77138000	2.50799300	5.53036600
C	1.50164600	0.46531400	5.93507600
C	1.41319100	1.54316100	3.81658700
C	2.32130400	2.50031300	4.22312100
C	2.38629300	1.47037300	6.35041000
H	1.11426300	1.57009800	2.78034900
H	2.67446900	3.23523100	3.50948200
H	2.81397000	1.38005600	7.34024500
H	3.46070300	3.26373400	5.88615700
C	-0.59009300	-2.18197700	5.79817800
H	0.08336100	-3.04337000	5.81674600
C	1.51070200	-0.69751900	6.82974900
H	1.59479500	-1.68797200	6.38903000
N	-1.33057600	-1.90356800	6.78607600
N	1.63586500	-0.55231700	8.08630100
O	-1.14451900	-2.76798000	7.85546400
O	1.86515600	-1.78741200	8.73722200

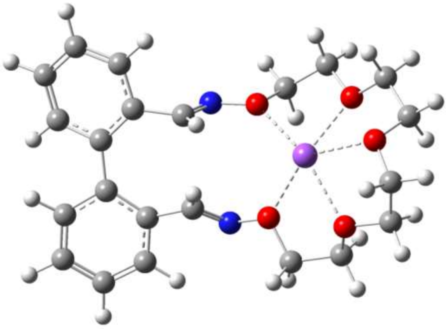

C	3.03799900	-1.64876000	9.53302500
H	3.91843400	-1.83571500	8.90801100
H	3.09037400	-0.62690600	9.91820500
C	2.98225200	-2.61625600	10.68099700
H	3.93322600	-2.57048300	11.22628500
H	2.17572300	-2.34496500	11.37674200
O	2.75549200	-3.91686800	10.17076700
C	-0.06980200	-6.05448300	11.15726300
H	-0.16240800	-7.08647300	11.51703900
H	0.21542000	-5.41846300	12.00404600
C	-1.38877200	-5.58903500	10.60981000
H	-1.70192100	-6.22833700	9.77372700
H	-2.15158900	-5.65008800	11.39590700
O	-1.23173100	-4.25564400	10.16476000
C	-2.41401000	-3.69101500	9.62650800
H	-2.87755100	-4.39433500	8.92297100
H	-3.13361500	-3.47120700	10.42485300
C	-2.04754500	-2.42470500	8.90913300
H	-1.56567500	-1.70776400	9.58249600
H	-2.93681300	-1.96141800	8.48017200
C	2.82092700	-4.93260800	11.15226600
H	3.86087800	-5.11688700	11.44982500
H	2.26758400	-4.62256500	12.04882900
C	2.22810900	-6.18596700	10.55456200
H	2.28397000	-7.00967800	11.27536900
H	2.79477900	-6.47434000	9.66655000
O	0.89368700	-5.97823100	10.12183400
Na	0.72871000	-3.84266900	8.90619200

3-Na^{+}-c

H (353 K, 1 atm) = -1500.066985 a.u.

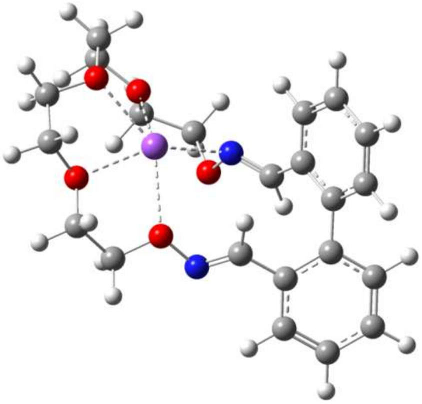

H	-4.47161000	3.62289300	-2.98050100
C	-3.45812700	3.24153300	-2.94665300
C	-0.84344700	2.25874800	-2.86313100
C	-3.18754600	2.06176500	-2.28055400
C	-2.43256900	3.92747200	-3.58624400
C	-1.13863500	3.43745500	-3.54456500
C	-1.88800500	1.55648800	-2.23680100
H	-3.98216000	1.50259200	-1.80174500
H	-2.64217400	4.84957700	-4.11532500
H	-0.33413300	3.98433500	-4.02327000
C	0.55280000	1.77664800	-2.79828700
C	3.16209300	0.76134200	-2.72831300
C	1.18220600	1.50946300	-1.56949500
C	1.26247000	1.54753100	-3.97476800
C	2.55227600	1.04439500	-3.94407700
C	2.48156000	0.99882000	-1.54914200
H	0.77889300	1.74507500	-4.92466400
H	3.08381300	0.87039100	-4.87223300
H	2.96220300	0.83013200	-0.59249200
H	4.17462800	0.37680400	-2.70257500
C	-1.59966800	0.27990800	-1.58985700
H	-0.73351500	-0.28698200	-1.93312000
C	0.50677300	1.84086600	-0.31644300
H	-0.20214000	2.66866500	-0.29836900
N	-2.31253000	-0.16918700	-0.63915400
N	0.74100400	1.19163800	0.75385000
O	-1.79364800	-1.37854800	-0.17862200
O	-0.00683800	1.71222200	1.82263300

C	0.81551700	1.89432000	2.96814600
H	1.82016800	2.19203800	2.65636800
H	0.36285700	2.72460500	3.51591100
C	0.88283700	0.69806200	3.88069500
H	1.46342900	0.98170200	4.76864400
H	-0.12272800	0.40640500	4.21391200
O	1.50611700	-0.38158800	3.21122900
C	0.85049600	-4.02445700	2.90751400
H	1.44329700	-4.92773600	3.09777000
H	0.43135900	-3.67952100	3.86046300
C	-0.27064300	-4.34364900	1.96309900
H	0.12357500	-4.71731500	1.00845800
H	-0.90113800	-5.12604300	2.40413400
O	-1.01579100	-3.16133400	1.74931600
C	-2.18463800	-3.37587400	0.98054400
H	-1.93674500	-3.93515000	0.06900200
H	-2.91936700	-3.95549500	1.55304300
C	-2.76676400	-2.04286100	0.61584500
H	-2.99429200	-1.44669500	1.50609100
H	-3.68734000	-2.18361100	0.04292600
C	1.99594600	-1.38398100	4.07655900
H	2.76298700	-0.97345300	4.74634000
H	1.18409600	-1.77919300	4.70256700
C	2.60685700	-2.46421400	3.21827800
H	3.05191800	-3.24208600	3.84925500
H	3.39772400	-2.03350800	2.60033600
O	1.65925200	-3.02074500	2.32249600
Na	0.36895000	-1.20466400	1.23415100

3-K$^+$-a

H (353 K, 1 atm) = -1937.638731 a.u.

H	-4.65975100	3.51717300	2.35326900
C	-3.62237200	3.22550900	2.46526000
C	-0.94768700	2.46724300	2.75947000
C	-3.20923500	1.97613000	2.04810700
C	-2.70920700	4.09842300	3.04420900
C	-1.38684400	3.71763100	3.19051200
C	-1.87677300	1.58058400	2.19047700
H	-3.91730400	1.27675700	1.62093500
H	-3.02858100	5.07750100	3.38127100
H	-0.66912200	4.40195000	3.62857800
C	0.48356400	2.11991700	2.92208900
C	3.17516400	1.46861100	3.32847300
C	1.32258100	1.85324500	1.82634500
C	1.02171400	2.06858100	4.20681200
C	2.35071600	1.74289900	4.41331800
C	2.66480100	1.52937000	2.04743500
H	0.37458900	2.27380400	5.05193500
H	2.74597200	1.70515000	5.42160000
H	3.30700300	1.35372600	1.19282000
H	4.21921100	1.22489100	3.48475300
C	-1.45928600	0.23641200	1.79883800
H	-0.52686400	-0.15981500	2.20430000
C	0.80443000	1.96787600	0.46526100
H	-0.13694800	2.49045800	0.29723700
N	-2.17258500	-0.46733900	1.01481000
N	1.44006500	1.46798700	-0.52083600
O	-1.59578800	-1.70708200	0.76519900
O	0.75007900	1.64555000	-1.71824500

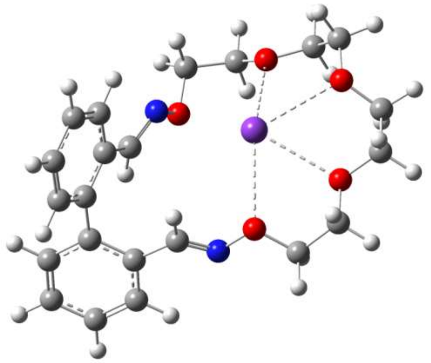

C	1.66211100	1.78193300	-2.80580300
H	2.67239200	1.56876500	-2.44967100
H	1.62503800	2.81667800	-3.16058600
C	1.29880100	0.84558400	-3.92341000
H	1.90207500	1.10389700	-4.80407600
H	0.23985000	0.95573900	-4.19478000
O	1.57223400	-0.47923800	-3.51331500
C	0.12502900	-4.04928900	-3.34157700
H	0.46933100	-4.91449100	-3.92300300
H	-0.57399700	-3.47428300	-3.96305200
C	-0.58587100	-4.53836800	-2.11267600
H	0.11500500	-5.07965000	-1.46226300
H	-1.37886700	-5.23529100	-2.41491700
O	-1.13778300	-3.43129900	-1.43136000
C	-1.91932200	-3.79908100	-0.31293300
H	-1.29286400	-4.27677400	0.45318600
H	-2.69599300	-4.51694500	-0.60785100
C	-2.59211200	-2.57658500	0.23747400
H	-3.14686900	-2.05539900	-0.54763000
H	-3.28932100	-2.86403800	1.03097300
C	1.46622800	-1.43187100	-4.55004400
H	2.07251800	-1.12727900	-5.41407800
H	0.42385100	-1.51639700	-4.88884900
C	1.98729300	-2.75046600	-4.03296300
H	2.02548800	-3.47859900	-4.85193900
H	3.00411100	-2.60939000	-3.65876300
O	1.22708000	-3.25535500	-2.95011900
K	0.61713100	-1.28618500	-1.02121900

3-K$^+$-b

H (353 K, 1 atm) = -1937.636103 a.u.

H	-2.49780900	-1.09566100	-4.96432900
C	-1.95893800	-0.31229400	-4.44495100
C	-0.57986200	1.72393700	-3.10898400
C	-2.10131500	-0.16365000	-3.07988900
C	-1.13916000	0.55944300	-5.15227900
C	-0.46177600	1.56778100	-4.48809800
C	-1.42019300	0.84878300	-2.39824800
H	-2.76552500	-0.81362200	-2.52276900
H	-1.02949900	0.45337700	-6.22513900
H	0.18526100	2.24202500	-5.03750700
C	0.17188100	2.81063500	-2.43562200
C	1.49986100	4.92091200	-1.17144700
C	1.17921900	2.54999500	-1.49069900
C	-0.13483500	4.13351300	-2.74564500
C	0.51847800	5.18151600	-2.12015600
C	1.83077100	3.61597600	-0.86550700
H	-0.91147400	4.33432900	-3.47489500
H	0.26117200	6.20362400	-2.37172000
H	2.61553400	3.40044800	-0.15074900
H	2.01600900	5.73856800	-0.68279700
C	-1.61050000	1.04605500	-0.96619700
H	-1.28642000	1.98716900	-0.52181800
C	1.57896200	1.17237200	-1.22203400
H	1.27144400	0.39872600	-1.92767400
N	-2.13973800	0.15548200	-0.22156200
N	2.29888800	0.86309200	-0.21795300
O	-2.14624500	0.58496200	1.10661400
O	2.57063900	-0.50286500	-0.20836000

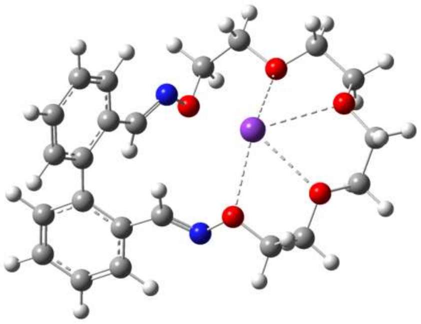

C	3.68561800	-0.77142800	0.63511100
H	4.08411500	-1.72198900	0.27486200
H	4.44160300	0.00561800	0.50366800
C	3.31074800	-0.89766400	2.08855500
H	4.22302600	-1.08423000	2.67048500
H	2.85609000	0.03163600	2.45756600
O	2.41298000	-1.98307400	2.21213500
C	-0.89341800	-3.39882500	3.87692200
H	-0.98680100	-4.34125400	4.43175000
H	-0.66909500	-2.59691000	4.59284200
C	-2.19472800	-3.10234800	3.18900200
H	-2.39300900	-3.86050800	2.41807400
H	-3.00760900	-3.14671300	3.92576100
O	-2.11966700	-1.81395100	2.61733800
C	-3.30923200	-1.40753600	1.97192000
H	-3.41722100	-1.91738900	1.00597500
H	-4.18683900	-1.65836000	2.58263700
C	-3.28230400	0.08973200	1.79898600
H	-3.22145100	0.57388400	2.77511300
H	-4.20033200	0.41622100	1.29933500
C	2.22247200	-2.45581400	3.52873400
H	3.18959300	-2.67136800	4.00267800
H	1.70691300	-1.70258100	4.14224700
C	1.41837500	-3.72929400	3.44111700
H	1.35291700	-4.19676800	4.42968000
H	1.92795200	-4.42576200	2.77030800
O	0.12615900	-3.50438400	2.90307800
K	0.04741100	-1.54834000	0.85051900

3-K^+-rac

H (353 K, 1 atm) = -1937.58631 a.u.

H	-4.22893900	-0.05113900	3.50164300
C	-3.15531400	-0.04670600	3.64370600
C	-0.33462500	0.03746100	4.11784200
C	-2.58583100	-0.70458100	4.71273600
C	-2.30579500	0.53672500	2.72385000
C	-0.94710100	0.56864100	2.96862500
C	-1.20313400	-0.72766600	4.93941800
H	-3.21024400	-1.27937000	5.38431800
H	-2.69081000	0.97801000	1.81224300
H	-0.34217800	1.03164900	2.20625000
C	1.10020700	0.43718700	4.36425900
C	3.75156600	1.51333000	4.58489600
C	2.00023000	0.00592400	5.37119200
C	1.59557800	1.47742600	3.55522200
C	2.87202500	1.99601200	3.63655900
C	3.29255800	0.54261400	5.44764200
H	0.94686900	1.95549600	2.84108400
H	3.16264000	2.79244000	2.96175800
H	3.94563300	0.16801000	6.22489400
H	4.76013900	1.89810800	4.67215800
C	-0.83519500	-1.79119200	5.88722000
H	-0.18253200	-2.58375700	5.51782900
C	1.76126400	-0.96864700	6.44979200
H	1.51286100	-2.00218300	6.22757100
N	-1.45664000	-1.93412600	6.98403700
N	2.01054300	-0.60041000	7.63834600
O	-1.19072500	-3.16419200	7.58592600
O	1.88956000	-1.62425000	8.56701400

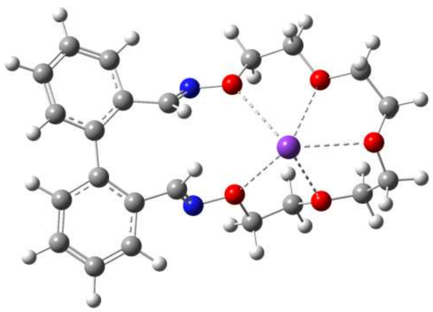

C	2.49396200	-1.18788400	9.78215800
H	3.58276400	-1.19498500	9.66736300
H	2.16886900	-0.16697600	9.99653800
C	2.08011000	-2.09364500	10.90435500
H	2.53983700	-1.71560000	11.82662800
H	0.98906900	-2.07458200	11.03779100
O	2.51031000	-3.41565000	10.64063200
C	0.36309000	-6.49840200	11.09233100
H	0.46222000	-7.52423300	11.46906000
H	0.15393700	-5.83932800	11.94270700
C	-0.78958800	-6.41967500	10.13070900
H	-0.63336000	-7.09225800	9.27523000
H	-1.70234200	-6.74249700	10.64773000
O	-0.90923400	-5.08146800	9.69219100
C	-2.13299000	-4.79610300	9.04531700
H	-2.26953000	-5.44662900	8.17029500
H	-2.97291100	-4.96855400	9.73100100
C	-2.14608700	-3.35524400	8.62616300
H	-1.91161600	-2.70130800	9.47282100
H	-3.13992800	-3.09762800	8.25247000
C	2.41222200	-4.26345100	11.76716300
H	3.19028300	-4.01474700	12.50121300
H	1.43796900	-4.12656100	12.25555500
C	2.58369400	-5.69307500	11.31433300
H	2.63737600	-6.35447100	12.18705700
H	3.52144300	-5.79377100	10.76257800
O	1.55084900	-6.10171700	10.43320500
K	1.32620600	-4.29894700	8.31147800

3-K^{+}-c

H (353 K, 1 atm) = -1937.638731 a.u.

H	-4.65975100	3.51717300	-2.35326900
C	-3.62237200	3.22550900	-2.46526000
C	-0.94768700	2.46724300	-2.75947000
C	-3.20923500	1.97613000	-2.04810700
C	-2.70920700	4.09842300	-3.04420900
C	-1.38684400	3.71763100	-3.19051200
C	-1.87677300	1.58058400	-2.19047700
H	-3.91730400	1.27675700	-1.62093500
H	-3.02858100	5.07750100	-3.38127100
H	-0.66912200	4.40195000	-3.62857800
C	0.48356400	2.11991700	-2.92208900
C	3.17516400	1.46861100	-3.32847300
C	1.32258100	1.85324500	-1.82634500
C	1.02171400	2.06858100	-4.20681200
C	2.35071600	1.74289900	-4.41331800
C	2.66480100	1.52937000	-2.04743500
H	0.37458900	2.27380400	-5.05193500
H	2.74597200	1.70515000	-5.42160000
H	3.30700300	1.35372600	-1.19282000
H	4.21921100	1.22489100	-3.48475300
C	-1.45928600	0.23641200	-1.79883800
H	-0.52686400	-0.15981500	-2.20430000
C	0.80443000	1.96787600	-0.46526100
H	-0.13694800	2.49045800	-0.29723700
N	-2.17258500	-0.46733900	-1.01481000
N	1.44006500	1.46798700	0.52083600
O	-1.59578800	-1.70708200	-0.76519900
O	0.75007900	1.64555000	1.71824500

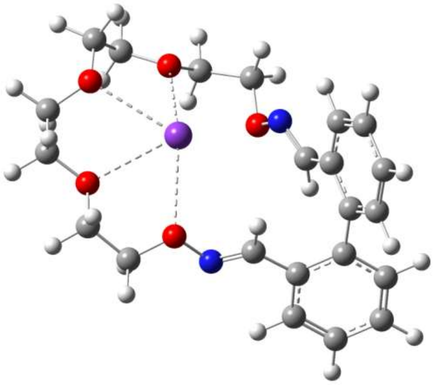

C	1.66211100	1.78193300	2.80580300
H	2.67239200	1.56876500	2.44967100
H	1.62503800	2.81667800	3.16058600
C	1.29880100	0.84558400	3.92341000
H	1.90207500	1.10389700	4.80407600
H	0.23985000	0.95573900	4.19478000
O	1.57223400	-0.47923800	3.51331500
C	0.12502900	-4.04928900	3.34157700
H	0.46933100	-4.91449100	3.92300300
H	-0.57399700	-3.47428300	3.96305200
C	-0.58587100	-4.53836800	2.11267600
H	0.11500500	-5.07965000	1.46226300
H	-1.37886700	-5.23529100	2.41491700
O	-1.13778300	-3.43129900	1.43136000
C	-1.91932200	-3.79908100	0.31293300
H	-1.29286400	-4.27677400	-0.45318600
H	-2.69599300	-4.51694500	0.60785100
C	-2.59211200	-2.57658500	-0.23747400
H	-3.14686900	-2.05539900	0.54763000
H	-3.28932100	-2.86403800	-1.03097300
C	1.46622800	-1.43187100	4.55004400
H	2.07251800	-1.12727900	5.41407800
H	0.42385100	-1.51639700	4.88884900
C	1.98729300	-2.75046600	4.03296300
H	2.02548800	-3.47859900	4.85193900
H	3.00411100	-2.60939000	3.65876300
O	1.22708000	-3.25535500	2.95011900
K	0.61713100	-1.28618500	1.02121900

3-$Et_2NH_2^+$-a

H (353 K, 1 atm) = -1551.856728 a.u.

H	-2.31489200	-0.83621700	-5.15649200
C	-1.82771900	-0.13683300	-4.48745200
C	-0.59723300	1.69756800	-2.76992800
C	-2.36631200	0.11448400	-3.24126200
C	-0.66927600	0.51964600	-4.88435800
C	-0.06622000	1.42685800	-4.03043700
C	-1.76448500	1.02406900	-2.36629400
H	-3.27135600	-0.38796600	-2.92199700
H	-0.24390900	0.33532400	-5.86376500
H	0.82968600	1.95219700	-4.34270200
C	0.08832200	2.70488700	-1.91349500
C	1.33469600	4.69456100	-0.38712100
C	1.30793200	2.44251000	-1.26186100
C	-0.48631500	3.96683900	-1.77376200
C	0.12493300	4.95398600	-1.02020800
C	1.91693000	3.44926500	-0.50632500
H	-1.42259900	4.17277000	-2.28042600
H	-0.33673200	5.93071200	-0.93577200
H	2.85948400	3.23438400	-0.01739900
H	1.82356400	5.46791300	0.19315800
C	-2.29945300	1.20917900	-1.01742000
H	-2.01516200	2.09483800	-0.44995700
C	1.88792300	1.10327600	-1.31151200
H	1.50377500	0.39681300	-2.04773800
N	-3.02476500	0.31048400	-0.47902900
N	2.77513600	0.72548500	-0.47693700
O	-3.32487500	0.62108700	0.82553800
O	3.05788600	-0.62500900	-0.64731900

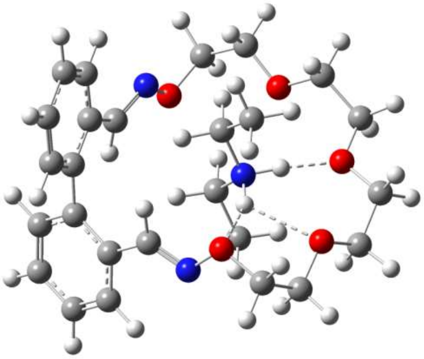

C	4.18834400	-1.01995900	0.12169500
H	4.42627100	-2.01679400	-0.25350000
H	5.02935000	-0.35215000	-0.08325100
C	3.93711800	-1.07175800	1.60783900
H	4.86758300	-1.38884300	2.09930900
H	3.68291300	-0.07355500	1.98769900
O	2.89354600	-1.98498200	1.88166100
C	-0.86390700	-3.20773500	3.46843200
H	-0.66831100	-4.26868400	3.66492100
H	-1.15198000	-2.72784500	4.41087300
C	-1.99464400	-3.13451800	2.47634000
H	-1.68177100	-3.58619000	1.52200600
H	-2.81649600	-3.74946700	2.87126700
O	-2.42823700	-1.81014100	2.27850800
C	-3.58707000	-1.75821800	1.46841000
H	-3.36638100	-2.11798500	0.45436300
H	-4.36663400	-2.41224500	1.88532400
C	-4.16347200	-0.36509100	1.40796100
H	-4.35168900	0.01038100	2.41503900
H	-5.11344800	-0.41266000	0.86497200
C	2.65583600	-2.14034600	3.26275600
H	3.54896600	-2.54185100	3.76184500
H	2.42638800	-1.16976600	3.72326500
C	1.52090800	-3.10090900	3.50076300
H	1.42120000	-3.24038000	4.58382400
H	1.74157300	-4.07425000	3.04516400
O	0.31056100	-2.58864800	2.96539600
N	0.39968200	-0.97896200	0.75768800
H	0.34941900	-1.64281700	1.55465200
H	1.39738000	-0.90882500	0.53793800
C	-0.12839600	0.33036400	1.21624500
H	-1.16388300	0.16727200	1.50450900
H	-0.10373000	1.00450800	0.36376700

C	0.68024600	0.89148400	2.35877700
H	0.65336900	0.23085000	3.22660900
H	1.71677900	1.06745300	2.06376500
C	-0.29042500	-1.57241600	-0.41178200
H	-1.33696900	-1.69152000	-0.13949500
H	-0.25349100	-0.84658300	-1.22367000
C	0.35682400	-2.87840300	-0.80823500
H	0.33243100	-3.60292600	0.00875200
H	1.39712800	-2.72828500	-1.10200200
H	-0.17776400	-3.30621100	-1.65701500
H	0.25369300	1.85036000	2.65552900

3-$Et_2NH_2^+$-TS-rac

H (353 K, 1 atm) = -1551.781752 a.u.

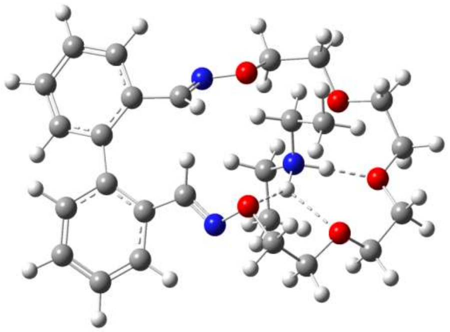

H	-3.60576700	2.83636000	-4.19951900
C	-2.70284200	3.05165900	-3.64172700
C	-0.23301900	3.51907900	-2.23543500
C	-2.17595200	2.12523200	-2.77024300
C	-2.07503700	4.27731100	-3.71530500
C	-0.90097900	4.48462300	-3.02095800
C	-1.00271700	2.33703900	-2.02774600
H	-2.68987200	1.18493400	-2.62612600
H	-2.49078000	5.08702700	-4.30319200
H	-0.51139700	5.48675100	-3.06868400
C	1.10642300	3.99266500	-1.69820400
C	3.11407900	5.69085000	-0.55819400
C	1.94418900	3.54024000	-0.63058900
C	1.59453200	5.16590100	-2.31829000
C	2.55235300	5.99961600	-1.78597100
C	2.84505700	4.44775100	-0.04243300
H	1.20572500	5.44573000	-3.28342500

H	2.84905500	6.88745300	-2.33156900
H	3.41531700	4.09433700	0.80588400
H	3.82093000	6.35165600	-0.07203900
C	-0.83952000	1.27643700	-1.01864800
H	0.06856400	0.69092100	-0.91949200
C	2.21325200	2.14556300	-0.25165700
H	1.83836400	1.33802300	-0.87573400
N	-1.88331800	0.98030200	-0.34313100
N	3.06030800	1.89305600	0.67368600
O	-1.72032500	-0.13616700	0.44468900
O	3.36679200	0.54048500	0.78570900
C	4.54725900	0.36934500	1.57540800
H	4.88970100	-0.63909200	1.33591300
H	5.30505400	1.08592000	1.25126400
C	4.33526200	0.50407600	3.06733800
H	5.31642500	0.44392200	3.55798300
H	3.90593100	1.48800200	3.29954300
O	3.48471800	-0.53030800	3.52664700
C	-0.24799000	-1.90716200	5.07819400
H	0.02916700	-2.82233400	5.61314800
H	-0.76588200	-1.24036700	5.77623300
C	-1.16950900	-2.30142700	3.94516500
H	-0.65763300	-3.01033000	3.27706900
H	-2.02934900	-2.82548000	4.38655300
O	-1.59311700	-1.17125600	3.21741900
C	-2.57180600	-1.47173900	2.23648400
H	-2.25868800	-2.33680800	1.63406100
H	-3.51856100	-1.73691700	2.72498000
C	-2.82456600	-0.29035900	1.32918900
H	-2.96900000	0.62229600	1.91630600
H	-3.73550300	-0.47275700	0.75065700
C	3.15107900	-0.42998200	4.89520900
H	4.04329300	-0.57675700	5.51922300

H	2.74931600	0.56830000	5.11693700
C	2.13373700	-1.47988700	5.27945300
H	1.97444100	-1.41193300	6.36186500
H	2.51134900	-2.48286700	5.04563100
O	0.91420100	-1.25213800	4.59051600
N	1.05481500	-0.14388000	2.16119700
H	0.97903000	-0.59035200	3.09390700
H	2.03378300	0.12706100	2.06341700
C	0.22773000	1.09023000	2.19190400
H	-0.77437400	0.79896800	2.47664100
H	0.20191200	1.51824700	1.19566500
C	0.79866200	2.07550100	3.18482900
H	0.82610700	1.65392300	4.19176300
H	1.80119100	2.40008100	2.89847700
C	0.84662700	-1.20160400	1.14144100
H	-0.17952900	-1.53474500	1.20400600
H	0.99116800	-0.75484300	0.16037400
C	1.81676600	-2.34179800	1.35499000
H	1.70215500	-2.78475500	2.34585300
H	2.85195200	-2.02113300	1.24753600
H	1.62163900	-3.11699100	0.61272400
H	0.16209400	2.96070000	3.21321800

3-$Et_2NH_2^+$-b

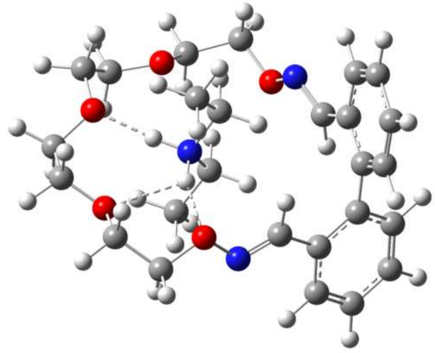

H (353 K, 1 atm) = -1551.856291 a.u.

H	-4.12776900	3.78152300	-0.66099200
C	-3.19706500	3.40736100	-1.07086900
C	-0.81425600	2.44134700	-2.16834800
C	-3.07323400	2.06953700	-1.38926900
C	-2.13228700	4.27231800	-1.29155100
C	-0.95611700	3.78812900	-1.83771000

C	-1.88718900	1.56517800	-1.93252900
H	-3.89839100	1.38722900	-1.22555200
H	-2.22354700	5.32525200	-1.05241100
H	-0.12762700	4.46186300	-2.02681600
C	0.46533300	1.98907000	-2.77869000
C	2.83963100	1.20572800	-4.03477500
C	1.63375300	1.78894100	-2.02384100
C	0.51157100	1.78068800	-4.15585700
C	1.68308600	1.39317300	-4.78249000
C	2.81216100	1.39965000	-2.66904900
H	-0.38904100	1.94005100	-4.73825700
H	1.69740000	1.24712900	-5.85616000
H	3.70708100	1.25584600	-2.07603100
H	3.76259000	0.91398100	-4.52144700
C	-1.74425900	0.12898900	-2.17867100
H	-0.96273000	-0.22691000	-2.84930600
C	1.59847900	1.91927500	-0.56828300
H	0.73794300	2.40178400	-0.10401600
N	-2.48953400	-0.69277800	-1.55227400
N	2.52273300	1.41655500	0.14992300
O	-2.17799500	-1.99889700	-1.82803800
O	2.23063000	1.51280900	1.50392900
C	3.35730100	1.11530800	2.28382000
H	3.95561000	0.40838500	1.70524500
H	3.96877900	1.99272800	2.51555200
C	2.86766900	0.47816300	3.55437100
H	3.73056100	0.30617100	4.21211300
H	2.17430400	1.15021500	4.07982600
O	2.22810300	-0.74064200	3.24085700
C	-1.13331900	-3.38590400	3.22826600
H	-0.92036500	-4.15403600	3.97854000
H	-2.09732800	-2.93125800	3.47184800
C	-1.23457100	-4.03901700	1.87134700

H	-0.24149900	-4.34451900	1.50812200
H	-1.83636800	-4.95241800	1.98493100
O	-1.85525300	-3.16313800	0.95757300
C	-2.25909200	-3.82190200	-0.22812300
H	-1.38644000	-4.21958800	-0.76636900
H	-2.90767700	-4.67252400	0.02510000
C	-3.03486400	-2.89432700	-1.12768300
H	-3.76215700	-2.31775400	-0.55354100
H	-3.56571900	-3.49395500	-1.87322100
C	1.77476100	-1.43930700	4.37737000
H	2.62298600	-1.71941600	5.01765900
H	1.09441800	-0.81148300	4.96977100
C	1.05776700	-2.69491200	3.95803100
H	0.84488900	-3.28447600	4.85706200
H	1.69814900	-3.29346900	3.29761900
O	-0.15761700	-2.35413300	3.30986800
N	0.17560800	-0.57646900	1.18869000
H	0.10256800	-1.29962100	1.92783500
H	0.95265600	0.02955800	1.47889200
C	-1.08155000	0.21128000	1.21042200
H	-1.89438100	-0.47810100	0.98659900
H	-1.03346700	0.93741400	0.40058800
C	-1.25656300	0.88908200	2.54921200
H	-1.30638100	0.16026600	3.36070600
H	-0.43732600	1.58328100	2.75174600
C	0.51449600	-1.22801900	-0.10072100
H	-0.31762500	-1.87724700	-0.35538100
H	0.58072300	-0.44615500	-0.85469700
C	1.81502100	-1.98775500	0.00028200
H	1.77157800	-2.75997000	0.77143000
H	2.64971200	-1.32171600	0.22429900
H	2.01995000	-2.47519700	-0.95362400
H	-2.18548100	1.46018000	2.54686500

3-Et$_2$NH$_2$$^+$-c

H (353 K, 1 atm) = -1551.856728 a.u.

H	-2.31489200	-0.83621700	5.15649200
C	-1.82771900	-0.13683300	4.48745200
C	-0.59723300	1.69756800	2.76992800
C	-2.36631200	0.11448400	3.24126200
C	-0.66927600	0.51964600	4.88435800
C	-0.06622000	1.42685800	4.03043700
C	-1.76448500	1.02406900	2.36629400
H	-3.27135600	-0.38796600	2.92199700
H	-0.24390900	0.33532400	5.86376500
H	0.82968600	1.95219700	4.34270200
C	0.08832200	2.70488700	1.91349500
C	1.33469600	4.69456100	0.38712100
C	1.30793200	2.44251000	1.26186100
C	-0.48631500	3.96683900	1.77376200
C	0.12493300	4.95398600	1.02020800
C	1.91693000	3.44926500	0.50632500
H	-1.42259900	4.17277000	2.28042600
H	-0.33673200	5.93071200	0.93577200
H	2.85948400	3.23438400	0.01739900
H	1.82356400	5.46791300	-0.19315800
C	-2.29945300	1.20917900	1.01742000
H	-2.01516200	2.09483800	0.44995700
C	1.88792300	1.10327600	1.31151200
H	1.50377500	0.39681300	2.04773800
N	-3.02476500	0.31048400	0.47902900
N	2.77513600	0.72548500	0.47693700
O	-3.32487500	0.62108700	-0.82553800

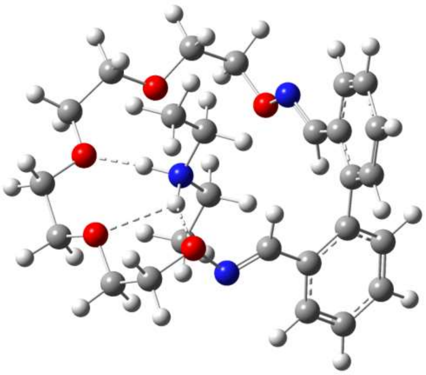

O	3.05788600	-0.62500900	0.64731900
C	4.18834400	-1.01995900	-0.12169500
H	4.42627100	-2.01679400	0.25350000
H	5.02935000	-0.35215000	0.08325100
C	3.93711800	-1.07175800	-1.60783900
H	4.86758300	-1.38884300	-2.09930900
H	3.68291300	-0.07355500	-1.98769900
O	2.89354600	-1.98498200	-1.88166100
C	-0.86390700	-3.20773500	-3.46843200
H	-0.66831100	-4.26868400	-3.66492100
H	-1.15198000	-2.72784500	-4.41087300
C	-1.99464400	-3.13451800	-2.47634000
H	-1.68177100	-3.58619000	-1.52200600
H	-2.81649600	-3.74946700	-2.87126700
O	-2.42823700	-1.81014100	-2.27850800
C	-3.58707000	-1.75821800	-1.46841000
H	-3.36638100	-2.11798500	-0.45436300
H	-4.36663400	-2.41224500	-1.88532400
C	-4.16347200	-0.36509100	-1.40796100
H	-4.35168900	0.01038100	-2.41503900
H	-5.11344800	-0.41266000	-0.86497200
C	2.65583600	-2.14034600	-3.26275600
H	3.54896600	-2.54185100	-3.76184500
H	2.42638800	-1.16976600	-3.72326500
C	1.52090800	-3.10090900	-3.50076300
H	1.42120000	-3.24038000	-4.58382400
H	1.74157300	-4.07425000	-3.04516400
O	0.31056100	-2.58864800	-2.96539600
N	0.39968200	-0.97896200	-0.75768800
H	0.34941900	-1.64281700	-1.55465200
H	1.39738000	-0.90882500	-0.53793800
C	-0.12839600	0.33036400	-1.21624500
H	-1.16388300	0.16727200	-1.50450900

H	-0.10373000	1.00450800	-0.36376700
C	0.68024600	0.89148400	-2.35877700
H	0.65336900	0.23085000	-3.22660900
H	1.71677900	1.06745300	-2.06376500
C	-0.29042500	-1.57241600	0.41178200
H	-1.33696900	-1.69152000	0.13949500
H	-0.25349100	-0.84658300	1.22367000
C	0.35682400	-2.87840300	0.80823500
H	0.33243100	-3.60292600	-0.00875200
H	1.39712800	-2.72828500	1.10200200
H	-0.17776400	-3.30621100	1.65701500
H	0.25369300	1.85036000	-2.65552900

9-crown-3

H (353 K, 1 atm) = -460.947802 a.u.

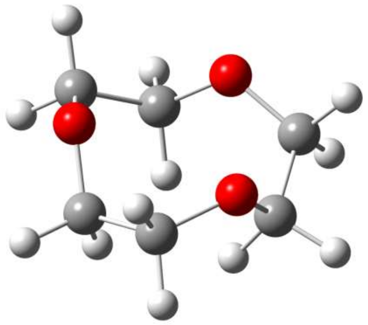

O	1.04662400	-4.49980100	5.84963100
O	1.05864100	-4.55759300	2.98570800
C	-0.06629800	-5.30029000	6.14631900
H	-0.28170600	-5.25671200	7.22371000
H	-0.96712900	-4.93783100	5.63819500
C	0.21183100	-6.75106200	5.78932800
H	1.03900700	-7.11234100	6.40430500
H	-0.68119300	-7.34668600	6.04056900
C	0.42484100	-5.68732800	2.44447600
H	1.22386900	-6.25520400	1.96280500
H	-0.30632500	-5.40789900	1.66813500
C	-0.27150900	-6.58313500	3.45549000
H	-0.63803900	-7.47030700	2.91915700
H	-1.15647100	-6.08905200	3.87237100

O	0.62186700	-6.97118100	4.46478600
C	0.24429900	-3.64796700	3.67620700
H	0.20233600	-2.69444000	3.13022000
H	-0.78856400	-4.00777000	3.74543600
C	0.81433200	-3.36508500	5.05629200
H	1.79368600	-2.89505000	4.94285800
H	0.15101900	-2.64553100	5.56388600

9-crown-3-Li⁺

H (353 K, 1 atm) = -468.3237 a.u.

O	1.07986100	-4.54202800	5.75490000
O	1.08541500	-4.62726600	3.06440800
C	-0.05537700	-5.30067500	6.17772300
H	-0.18182300	-5.20007000	7.25924000
H	-0.96066000	-4.91554100	5.70110200
C	0.18270400	-6.75647300	5.81502600
H	0.95801700	-7.18893100	6.45119500
H	-0.72935600	-7.34409400	5.95504900
C	0.39506000	-5.69975600	2.42242000
H	1.16079100	-6.24035500	1.86182300
H	-0.34045600	-5.32431700	1.70493900
C	-0.26542900	-6.61263200	3.44123800
H	-0.56311900	-7.55122300	2.96561500
H	-1.16470200	-6.15410400	3.86091000
O	0.67783700	-6.87996200	4.48141600
Li	2.08414900	-5.54772700	4.44180300
C	0.25946400	-3.62370800	3.65929100
H	0.28847000	-2.71605100	3.05012600
H	-0.77801300	-3.96633900	3.69446300
C	0.79202800	-3.33296300	5.05194000
H	1.74328900	-2.79926500	4.99566900

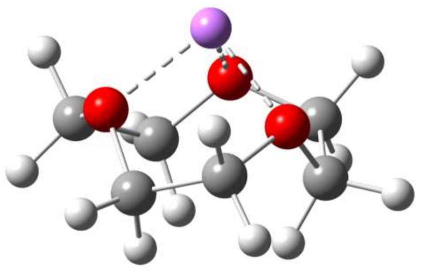

H	0.09111600	-2.70651200	5.61139100

9-crown-3-Na^{+}

H (353 K, 1 atm) = -623.008863 a.u.

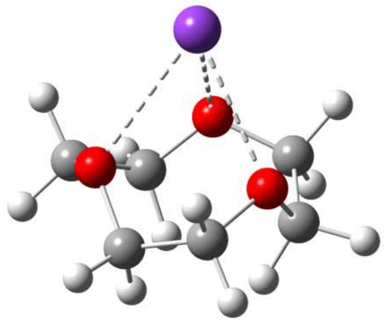

O	1.05632500	-4.51886700	5.79145700
O	1.06328900	-4.60054100	3.02955400
C	-0.07527600	-5.29478600	6.16797000
H	-0.23485700	-5.21270800	7.24786900
H	-0.97505100	-4.91229400	5.67894100
C	0.16461000	-6.75108000	5.80716800
H	0.93917200	-7.17700600	6.45040500
H	-0.75023800	-7.33036500	5.97382900
C	0.37730200	-5.69089900	2.42816100
H	1.13928400	-6.24041800	1.86911100
H	-0.36693800	-5.34103800	1.70430100
C	-0.28474700	-6.60191200	3.44804600
H	-0.60920000	-7.52231000	2.95207800
H	-1.17616100	-6.13293500	3.87285000
O	0.64410000	-6.91611800	4.47901800
C	0.23757700	-3.62945200	3.66163100
H	0.23964700	-2.70597400	3.07378700
H	-0.79609500	-3.98264600	3.70394000
C	0.77175700	-3.33744700	5.05374400
H	1.72149600	-2.79991200	4.98888300
H	0.06984700	-2.69191400	5.59307000
Na	2.60342300	-5.63937000	4.44587500

9-crown-3-K^+

H (353 K, 1 atm) = -1060.584967 a.u.

O	1.03068700	-4.50870600	5.80325300
O	1.03808900	-4.58805200	3.01852600
C	-0.09808000	-5.28932400	6.16243800
H	-0.27135300	-5.21459100	7.24160300
H	-0.99719700	-4.91000400	5.66912200
C	0.14568500	-6.74478700	5.80276900
H	0.92377700	-7.16326400	6.44719600
H	-0.76751400	-7.32431100	5.98439100
C	0.35827700	-5.68497500	2.43244700
H	1.12151300	-6.23978800	1.87973800
H	-0.38649600	-5.34836100	1.70116600
C	-0.30714800	-6.59369200	3.45155700
H	-0.64241100	-7.50677800	2.94749900
H	-1.19660800	-6.11996900	3.87588600
O	0.61524700	-6.92401600	4.47751700
C	0.21331400	-3.63061700	3.66313600
H	0.20363200	-2.70078100	3.08390400
H	-0.81954000	-3.98646500	3.71029400
C	0.75131300	-3.33893500	5.05335100
H	1.70259600	-2.80448100	4.98092300
H	0.05326200	-2.68114500	5.58514900
K	3.08821900	-5.72695100	4.44982100

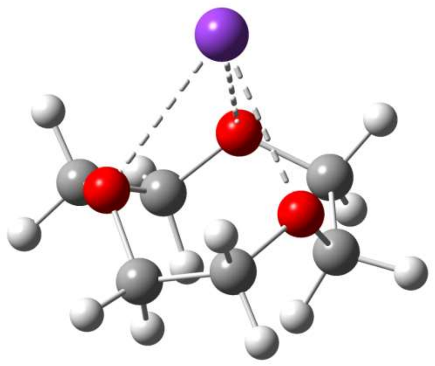

12-crown-4

H (353 K, 1 atm) = -614.607723 a.u.

O	-0.33848200	-3.39536100	7.01649600
O	2.06361400	-1.74165900	8.21222100
C	2.58841200	-3.04683000	8.31730500
H	2.04041400	-3.75474500	7.68514300
H	3.61109000	-2.96753300	7.93686600
C	2.64979700	-3.59606800	9.73149000
H	3.46366400	-4.32999200	9.79080200
H	2.88454200	-2.78026400	10.42601900
O	1.44761100	-4.24485600	10.09317700
C	0.76431000	-3.74629400	11.21000900
H	1.20305000	-4.11202600	12.15211800
H	0.79949500	-2.64814200	11.23136100
C	-0.67698200	-4.19403700	11.11467700
H	-0.73115300	-5.28913500	11.15739400
H	-1.22911700	-3.79929900	11.97405900
O	-1.28931100	-3.70917700	9.94247700
C	-1.33366300	-4.64056200	8.88068700
H	-0.40994900	-5.22551000	8.83884300
H	-2.17931800	-5.32976300	9.03234900
C	-1.51997200	-3.93807300	7.55701700
H	-2.29542600	-3.16666800	7.65726600
H	-1.88140200	-4.68212800	6.83792700
C	0.66844400	-1.57780600	8.35263200
H	0.52585600	-0.50044600	8.48485400
H	0.27748500	-2.08131800	9.23979500
C	-0.14461400	-2.00409500	7.14610900
H	-1.11633400	-1.49110500	7.18533600

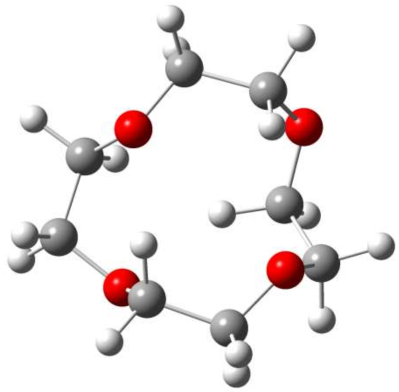

H	0.37343000	-1.67297200	6.23999800

12-crown-4-Li$^+$

H (353 K, 1 atm) = -622.007174 a.u.

O	-0.72368500	-3.53259200	7.27951500
O	1.83960600	-2.86027000	7.77517700
C	2.86916700	-2.59876800	8.72548600
H	3.68782000	-3.27250200	8.46641500
H	3.23062900	-1.56953700	8.64251500
C	2.38758700	-2.87190800	10.13226100
H	3.22651700	-2.79620800	10.83263300
H	1.62336200	-2.14372300	10.43126800
O	1.82563900	-4.18141600	10.15188800
C	1.03495900	-4.47431100	11.30412700
H	1.53931700	-5.22154000	11.92156500
H	0.89975700	-3.56569700	11.89699000
C	-0.31562700	-4.97892400	10.83777000
H	-0.24167000	-5.98689200	10.40491100
H	-1.01947100	-5.01941600	11.67570400
O	-0.72503200	-4.04977000	9.85055500
C	-1.94292200	-4.27086100	9.16071700
H	-1.99256700	-5.30228600	8.78696500
H	-2.80775100	-4.09211900	9.80778100
C	-1.91673100	-3.28474800	8.01875300
H	-1.90767800	-2.26610700	8.42172700
H	-2.79376700	-3.40503300	7.37599300
Li	0.70060600	-4.22730200	8.48914400
C	0.93845000	-1.79958200	7.47547700
H	1.45398500	-1.00067800	6.93187900
H	0.51587800	-1.37909100	8.39746000
C	-0.14671800	-2.40966100	6.61429900

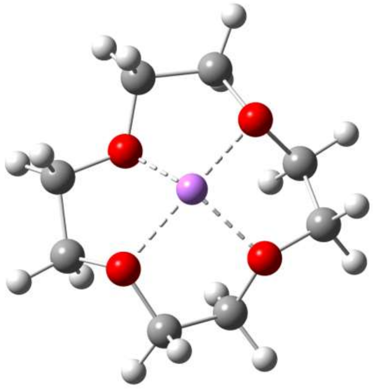

H	-0.90641900	-1.66518600	6.36257800
H	0.28544900	-2.78843500	5.68617200

12-crown-4-Na^{+}

H (353 K, 1 atm) = -776.686464 a.u.

O	-0.78167400	-3.57350100	7.15010400
O	1.90840400	-2.87025400	7.69235300
C	2.84271300	-2.58776400	8.72660200
H	3.71374900	-3.21568700	8.52551700
H	3.16591700	-1.54189500	8.68153700
C	2.29360000	-2.88093100	10.10220800
H	3.05348700	-2.62573000	10.85047600
H	1.40411700	-2.27188600	10.29650900
O	1.95465500	-4.26116800	10.19846500
C	1.10578000	-4.56115000	11.30184400
H	1.54942000	-5.37326600	11.88320200
H	1.01998200	-3.68737600	11.95279300
C	-0.26907500	-4.96709800	10.80432500
H	-0.24586200	-5.97668200	10.35860400
H	-0.97722100	-5.00430900	11.64104500
O	-0.64004700	-4.01323500	9.83560700
C	-1.87081900	-4.20936700	9.17215000
H	-1.96376700	-5.24920600	8.82245300
H	-2.72145500	-4.00268000	9.83221300
C	-1.87451700	-3.25611900	8.00450200
H	-1.77738200	-2.23374300	8.38404000
H	-2.81569300	-3.33363000	7.45063700
C	0.92578700	-1.87073600	7.46425600
H	1.38244500	-0.99650100	6.98487200
H	0.47633400	-1.54846600	8.41127200

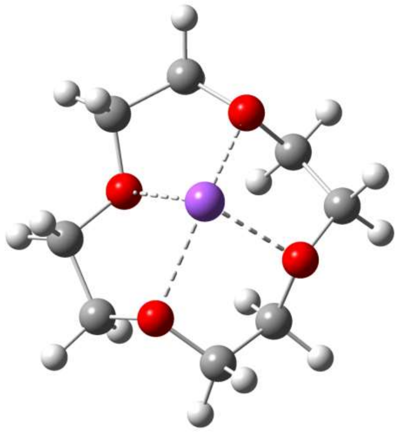

C	-0.13178200	-2.45537300	6.55572100
H	-0.85653600	-1.68295300	6.28015400
H	0.33112500	-2.82162300	5.63640400
Na	0.92900000	-4.86481100	8.12959200

12-crown-4-K^+

H (353 K, 1 atm) = -1214.25798 a.u.

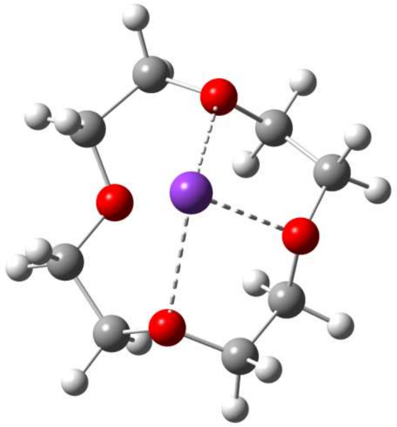

O	-0.82296200	-3.55438400	7.11215400
O	1.90298800	-2.86909100	7.69256100
C	2.82762100	-2.58707700	8.73137100
H	3.70308600	-3.21318600	8.54023200
H	3.15401100	-1.54119700	8.68434000
C	2.27843300	-2.86983600	10.10774000
H	3.02385100	-2.56589900	10.85374600
H	1.36848800	-2.28622100	10.27921700
O	1.98988300	-4.25481200	10.24355500
C	1.11075100	-4.53829000	11.32185300
H	1.52250000	-5.36533500	11.90668600
H	1.03443200	-3.67019600	11.98257700
C	-0.26469300	-4.91290700	10.80529600
H	-0.24738100	-5.91619500	10.34259100
H	-0.97441800	-4.96908500	11.64152000
O	-0.63627700	-3.94581900	9.85806900
C	-1.84829500	-4.17508200	9.18068300
H	-1.91207500	-5.22076100	8.83809400
H	-2.71411500	-3.99253000	9.83013700
C	-1.88445000	-3.23852100	8.00155500
H	-1.78799000	-2.21087000	8.36656400
H	-2.84359400	-3.32803700	7.47995600
C	0.90477500	-1.88229200	7.50007400
H	1.34793100	-0.97928100	7.06056800

H	0.44478300	-1.60738800	8.45631400
C	-0.14480600	-2.43411900	6.56466300
H	-0.85165600	-1.64158400	6.29589900
H	0.32748000	-2.77863100	5.64096200
K	1.12238300	-5.38851600	7.81047800

15-crown-5

H (353 K, 1 atm) = -768.267015 a.u.

O	-1.02938000	-2.44341200	8.14039400
O	1.84761000	-1.88619600	8.37214500
C	3.00237800	-1.86682500	9.16872900
H	3.79435400	-2.32249600	8.56909600
H	3.30091000	-0.83142100	9.39846500
C	2.87937500	-2.63373300	10.46593700
H	3.77190500	-2.42542800	11.08055400
H	2.00544400	-2.28969700	11.04036500
O	2.77043400	-4.00042600	10.19016900
C	-0.13518700	-6.32517900	11.16644300
H	-0.32197700	-7.35246800	11.51629100
H	0.03765400	-5.69784400	12.05081200
C	-1.35412000	-5.80877900	10.45234900
H	-1.43937900	-6.30484100	9.47449400
H	-2.25237300	-6.05972400	11.04028300
O	-1.23392800	-4.42172200	10.29371600
C	-2.28858900	-3.85110800	9.56782100
H	-2.47330200	-4.41214200	8.64036600
H	-3.21729200	-3.86127900	10.16117500
C	-1.93226100	-2.43317500	9.21363300
H	-1.50291200	-1.95062700	10.10282200
H	-2.84509700	-1.88047500	8.93948100

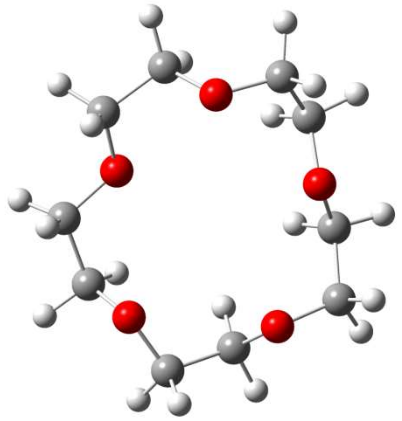

C	2.54699700	-4.77475800	11.33434200
H	3.44260500	-4.79035100	11.97852800
H	1.72466400	-4.34782300	11.92882500
C	2.21091500	-6.18915000	10.92053800
H	2.27214300	-6.84654400	11.80127100
H	2.95415900	-6.52863200	10.19477700
O	0.96115600	-6.30053200	10.29086400
C	-0.37916000	-1.21829400	7.92099400
H	-0.05583500	-1.21900900	6.87687100
H	-1.07162400	-0.37455900	8.06299100
C	0.83445600	-1.01820400	8.79952800
H	1.16778100	0.03064300	8.72142900
H	0.56510900	-1.19841000	9.84975800

15-crown-5-Li^+

H (353 K, 1 atm) = -775.687977 a.u.

O	-0.57362100	-2.79703200	8.22781900
O	1.21856000	-1.67289600	9.91798200
C	2.62864700	-1.65510200	9.78161000
H	2.91963500	-1.82248100	8.73654400
H	3.03808900	-0.69280900	10.10889300
C	3.15854200	-2.76396500	10.64384300
H	4.24699600	-2.83961000	10.54154900
H	2.91558200	-2.57669300	11.69762100
O	2.52698700	-3.95343700	10.20730000
C	-0.14020700	-5.96778400	11.32753900
H	-0.17441900	-7.00054500	11.69260700
H	0.21758100	-5.32530300	12.14342500
C	-1.50656100	-5.53218500	10.87574800
H	-1.86801600	-6.21989400	10.10311300
H	-2.21536300	-5.53329000	11.71034300

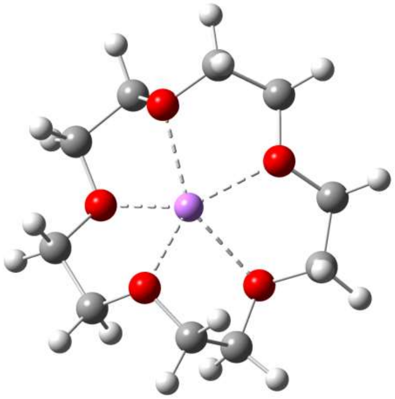

O	-1.37315800	-4.22829400	10.33016900
C	-2.32521200	-3.92223200	9.32452000
H	-2.33581300	-4.72123600	8.57294800
H	-3.33077700	-3.82544900	9.74917200
C	-1.90466300	-2.62567800	8.69569500
H	-1.95513900	-1.82189600	9.43845400
H	-2.57197800	-2.37520800	7.86371800
C	2.83648600	-5.09814000	10.97990400
H	3.90930200	-5.32081900	10.94056900
H	2.56536500	-4.92153900	12.02939800
C	2.04766400	-6.23654100	10.37813600
H	2.12895100	-7.13488600	10.99911900
H	2.43240300	-6.46872000	9.38350900
O	0.69494500	-5.86091400	10.18807100
C	0.06286200	-1.59351500	7.82141900
H	0.91437300	-1.90148600	7.21056000
H	-0.60195400	-0.99025800	7.19316500
C	0.52489900	-0.80789700	9.03256300
H	1.15070700	0.03599700	8.72478600
H	-0.32015200	-0.40090600	9.59154500
Li	0.52489200	-3.75900900	9.69118300

15-crown-5-Na$^+$

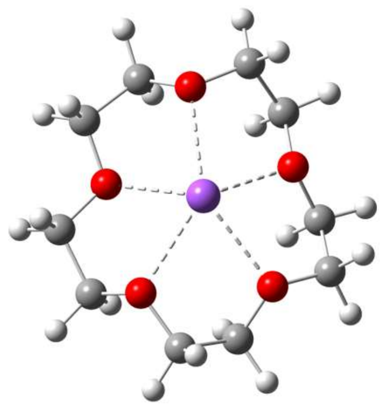

H (353 K, 1 atm) = -930.372908 a.u.

O	-0.92921600	-2.52901100	8.18900400
O	1.78241800	-1.97624700	8.55281300
C	2.99130900	-1.83869000	9.28302300
H	3.78417700	-2.23403200	8.64401400
H	3.20986800	-0.78531000	9.48685100
C	2.95327000	-2.62080600	10.57389800

H	3.92431100	-2.53929200	11.07876200
H	2.18352600	-2.23238600	11.25643700
O	2.66965900	-3.96524000	10.24041900
C	-0.13570900	-6.26822400	11.14703200
H	-0.23173400	-7.31179800	11.46960300
H	0.03694400	-5.64575800	12.03328500
C	-1.40270100	-5.82249300	10.47250000
H	-1.60642300	-6.43947300	9.58672900
H	-2.24407400	-5.93198700	11.16783100
O	-1.23118600	-4.46925400	10.09886700
C	-2.36496700	-3.87736900	9.49581900
H	-2.66126600	-4.44314200	8.60227800
H	-3.21267100	-3.86246600	10.19190200
C	-1.99482100	-2.46998600	9.12047800
H	-1.69402500	-1.91808400	10.02040000
H	-2.86154500	-1.96512000	8.67741400
C	2.66951300	-4.85848800	11.33609600
H	3.67484900	-4.94448200	11.76764600
H	1.99612000	-4.48908100	12.12186100
C	2.22119400	-6.20534000	10.81920200
H	2.24186700	-6.94547600	11.62650700
H	2.90306900	-6.54144600	10.03498700
O	0.93351000	-6.13467600	10.22642000
Na	0.76601200	-4.14119400	8.82687800
C	-0.35669600	-1.26943300	7.87020100
H	-0.01900600	-1.33311300	6.83339300
H	-1.10367700	-0.47159700	7.93810300
C	0.82159600	-0.95708100	8.76033500
H	1.23605900	0.02161600	8.48814300
H	0.51687700	-0.91569800	9.81540700

15-crown-5-K^+

H (353 K, 1 atm) = -1367.940279 a.u.

O	-0.82296200	-3.55438400	7.11215400
O	1.90298800	-2.86909100	7.69256100
C	2.82762100	-2.58707700	8.73137100
H	3.70308600	-3.21318600	8.54023200
H	3.15401100	-1.54119700	8.68434000
C	2.27843300	-2.86983600	10.10774000
H	3.02385100	-2.56589900	10.85374600
H	1.36848800	-2.28622100	10.27921700
O	1.98988300	-4.25481200	10.24355500
C	1.11075100	-4.53829000	11.32185300
H	1.52250000	-5.36533500	11.90668600
H	1.03443200	-3.67019600	11.98257700
C	-0.26469300	-4.91290700	10.80529600
H	-0.24738100	-5.91619500	10.34259100
H	-0.97441800	-4.96908500	11.64152000
O	-0.63627700	-3.94581900	9.85806900
C	-1.84829500	-4.17508200	9.18068300
H	-1.91207500	-5.22076100	8.83809400
H	-2.71411500	-3.99253000	9.83013700
C	-1.88445000	-3.23852100	8.00155500
H	-1.78799000	-2.21087000	8.36656400
H	-2.84359400	-3.32803700	7.47995600
C	0.90477500	-1.88229200	7.50007400
H	1.34793100	-0.97928100	7.06056800
H	0.44478300	-1.60738800	8.45631400
C	-0.14480600	-2.43411900	6.56466300
H	-0.85165600	-1.64158400	6.29589900

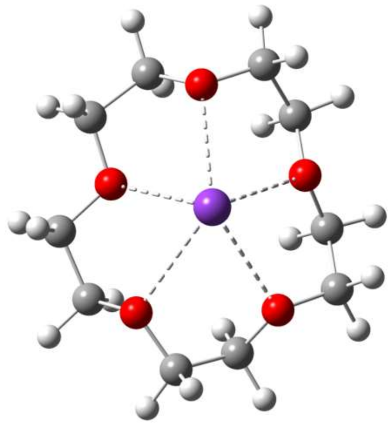

H	0.32748000	-2.77863100	5.64096200
K	1.12238300	-5.38851600	7.81047800
